# Recent Advances in Electrochemical Detection of Antibiotics on Graphene-Based Sensors and Biosensors, Impact and Sustainable Development Challenges: A Systematic Review and Meta-Analysis

**DOI:** 10.3390/bios16050234

**Published:** 2026-04-23

**Authors:** Muhammad Saqib, Mrinal Vashisth, Elena I. Korotkova, Amrit L. Hui, Stephen O. Aremu, Souvik Das, Aniruddha Deb, Nirmal K. Hazra, Rachita Saha, Subrata Saha, Pradip Kumar Kar

**Affiliations:** 1 Chemical Engineering Division, School of Earth Sciences and Engineering, National Research Tomsk Polytechnic University, 30 Lenin Avenue, 634050 Tomsk, Russia; 2 UNESCO Laboratory of Environmental Electrochemistry, Department of Analytical Chemistry, Faculty of Science, Charles University, Hlavova 8/2030, CZ 128 43 Prague 2, Czech Republic; 3 Higher Engineering School of Agrobiotech, National Research Tomsk State University, 36 Lenin Avenue, 634050 Tomsk, Russia; 4 National Research Tomsk State University, 36 Lenin Avenue, 634050 Tomsk, Russia; 5 Faculty of General Medicine, Siberian State Medical University, 634050 Tomsk, Russia; 6 Department of Industrial Design, National Institute of Technology Rourkela, Sundargarh 769008, India; 7 Department of Chemical Engineering, Birla Institute of Technology Mesra, Ranchi 835215, India; 8 Department of Chemistry, Coastal Environmental Studies Research Centre, Egra Sarada Shashi Bhusan College, Vidyasagar University, Egra 721429, India; 9 Parasitology Laboratory, Department of Zoology, Cooch Behar Panchanan Barma University, Vivekananda Street, Cooch Behar 736101, India

**Keywords:** antibiotic, systematic review, graphene oxide, reduced graphene oxide, electrochemical method, point-of-care diagnostics, antimicrobial resistance monitoring, environmental biosensor, sustainable development goals, sensors

## Abstract

The increasing use of antibiotics around the globe has contributed to an increase in antimicrobial resistance and become a major risk to both public health and sustainable development. Reliable and fast detection of antibiotic residues in clinical, agricultural, and environmental matrices is required to monitor antimicrobial resistance effectively. The conventional analytical techniques are sensitive, but they are also expensive, complex and lacking in portability. Voltammetry is a recently emerging electrochemical detection technique that is low-cost and rapid. To the best of our knowledge, for the first time, a meta-analysis was conducted on graphene-based electrochemical sensors and biosensors for antibiotic detection over the last decade. This systematic review critically examines the analytical properties of sensors and biosensors, the physicochemical properties of antibiotics, adsorption characteristics, and the use of nanoparticles to improve the selectivity and sensitivity of devices. This review critically examines the cost-effectiveness, scalability, and practicality of point-of-use devices using graphene-based sensors and biosensors. This systematic review also discusses the potential risks to human health from antibiotic contamination and the role of monitoring in contributing to achieving the UN’s Sustainable Development Goals. This systematic review identifies a gap between developing sensors in laboratories versus their deployment as field-deployable devices; it highlights challenges associated with stability, matrix effects and the complexity of manufacturing devices. Finally, it provides recommendations for future research that may help to address this gap to promote the transition of innovative devices from academic to practical applications.

## 1. Introduction

Antibiotics and antibacterial agents have been among the most transformative discoveries in medical science, playing indispensable roles in safeguarding human health, ensuring animal welfare, and sustaining agricultural productivity [1,2,3]. Their extensive use in clinical medicine has drastically reduced mortality and morbidity from infectious diseases, while in veterinary practice and the food industry, they are employed not only to treat infections but also as prophylactic agents and growth promoters [4,5]. However, global consumption of antibiotics has escalated dramatically over the past decades, with estimates suggesting that more than 92,700 tonnes were used in 2015, and rising by over 60 percent by 2016 [6]. This exponential increase reflects not only the demands of a growing human population but also the intensification of industrial livestock farming, yet the uncontrolled and often indiscriminate use of these drugs has contributed to one of the most pressing global health challenges: antimicrobial resistance (AMR) [7]. The United Nations and the World Health Organization (WHO) have recognized AMR as a grave threat to global health, security, and development, introducing action plans that call for rational antibiotic use, enhanced protocols and action plans to regulate antibiotic use, enhanced surveillance, and investment in novel diagnostic and monitoring technologies [8]. Central to any effective surveillance and control strategy is the reliable detection of antibiotics in clinical, agricultural, and environmental settings [4,5].

Conventional methods for antibiotic detection, such as high-performance liquid chromatography (HPLC), gas chromatography–mass spectrometry (GC-MS), gel electrophoresis, and thin-layer chromatography, are well-established and capable of delivering highly sensitive and precise results [9]. However, these techniques are often hampered by the need for costly instrumentation, skilled operators, laborious sample preparation, and the inability to provide rapid or field-based analyses [10]. However, electrochemical techniques have gained prominence as alternatives due to their inherent advantages of low cost, portability, rapid response, and suitability for real-time monitoring [11,12,13,14,15,16,17,18]. The performance of electrochemical sensors is highly dependent on the transducer materials employed, and in recent years, graphene and its derivatives have emerged as exceptional candidates [19,20,21,22,23,24]. With their remarkable electronic conductivity, large surface-to-volume ratio, mechanical robustness, and surface tunability, graphene-based materials enable efficient electron transfer and functionalization, making them superior to many traditional nanomaterials in the development of sensitive, selective, and robust sensing devices [25,26,27,28]. Graphene-based electrochemical methods can potentially be miniaturized, integrated into portable systems, and adapted for the in situ monitoring of pollutants in the environment, whereas the other methods are less likely to be, due to the potential issues of reproducibility, long-term stability, and interference from complex matrices [29]. There have been many studies on the development of sensors for the detection of antibiotics by using different types of sensor materials [30,31,32]. Metal-based sensors, such as those employing silver or gold nanoparticles [33,34], demonstrate high catalytic activity and sensitivity, while metal oxides like zinc oxide and titanium dioxide provide chemical stability and high surface reactivity [35]. Conductive polymers, including polyaniline and polypyrrole, offer biocompatibility and tunable electrical properties that enhance selectivity, and carbon nanotubes, owing to their exceptional conductivity and high surface area, have been successfully applied in numerous electrochemical sensing platforms [35,36].

Although graphene-based sensors and biosensors have shown considerable promise analytically, a significant discrepancy exists between sensor development in the lab and the deployment of sensors in the field. While many previous reviews [37,38,39,40] focused on the sensitivity of graphene-based sensors and the synthesis of materials, they also neglected to address several practical issues that exist when scaling up to deploy sensors in the field; specifically, cost, scalability, and stability in complex matrices. In order to begin to close this gap, this study addresses these gaps by: (i) conducting a qualitative meta-analysis of 94 studies across 12 antibiotic classes; (ii) applying a WHO-aligned Affordable, Sensitive, Specific, User-friendly, Rapid and robust, Equipment-free, and Deliverable/Point-of-care (ASSURED/POC) readiness framework; (iii) evaluating fabrication economics and scalability pathways; and (iv) contextualizing sensor deployment within SDG targets for health, water, and equity. This holistic, policy-integrated approach distinguishes our work from prior technology-centric surveys.

**Figure 1:**
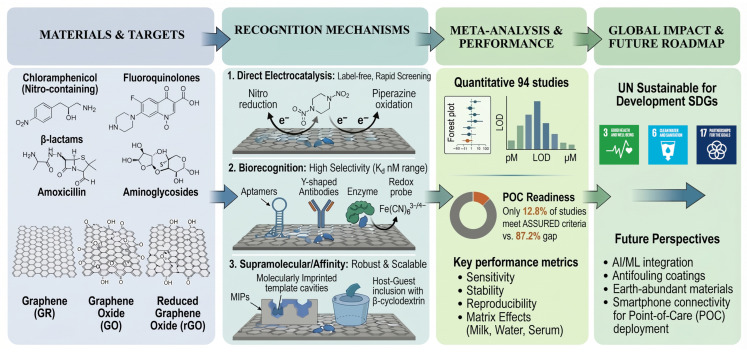
Comprehensive overview and roadmap for graphene-based electrochemical sensors and biosensors in antibiotic detection.

To our knowledge, this is the first descriptive meta-analysis specifically evaluating graphene (GR), graphene oxide (GO), and reduced graphene oxide (RGO)-based electrochemical platforms’ performance metrics for antibiotic detection. Additionally, a meta-analysis for antibiotic groups with nanomaterial types in a random-effects model was also performed for all examined studies. Unlike prior reviews, which predominantly emphasize synthesis routes or isolated sensitivity metrics, this work introduces a standardized multi-criteria assessment framework integrating analytical performance, POC readiness, economic viability, and real-sample matrix effects. Furthermore, it uniquely bridges sensor development with global health policy by mapping antibiotic monitoring to UN Sustainable Development Goals (SDGs), offering a translational roadmap from laboratory proof-of-concept to field-deployable, scalable devices (Figure 1).

This review is organized as follows: Section 2 describes the materials and methods regarding data collection and the performed meta-analyses. Section 3 focuses on the molecular and nanoscale interactions governing antibiotic recognition on graphene-based platforms, including adsorption mechanisms, nanoscale characterization, and nanoparticle integration for enhanced signal transduction. Section 4 critically reviews electrochemical applications of graphene-based sensors and biosensors for antibiotic detection. Section 5 critically reviews graphene oxide-based sensors and biosensors, and Section 6 critically reviews reduced graphene oxide-based sensors and biosensors. Section 7 provides, to the best of our knowledge, a comprehensive meta-analysis of the sensors and biosensors reviewed in Section 4, Section 5 and Section 6, focusing on their key performance metrics, limit of detection (LOD) and linear dynamic range (LDR), comparative analytical performance grouped by antibiotic, and advantages/disadvantages. Additionally, Section 8 provides a comparative analysis of the examined sensors and biosensors regarding transducer architecture, practical deployability, point-of-care readiness, and economic viability. Section 9 discusses the human health implications of antibiotic contamination and resistance. Section 10 discusses the role of antibiotics and monitoring technologies in the context of the UN Sustainable Development Goals. Finally, Section 11 presents a summary and future prospects for bridging the gap between laboratory innovation and field-deployable devices.

## 2. Materials and Methods

This systematic literature review was conducted in accordance with the Preferred Reporting Items for Systematic reviews and Meta-Analysis (PRISMA) 2020 [41]. The PRISMA flow diagram is mentioned in Figure S1 in the Supplementary Materials. While the quantitative meta-analysis was performed according to Cochrane handbook guidelines [42]. The methodology, including the search strategy and information sources, eligibility criteria and selection process, assessment of risk of bias, data collection process, and specifics regarding the meta-analysis implementation, is detailed in Section S1 in the Supplementary Materials, and the data fields are summarized in Table S2 in the Supplementary Materials.

## 3. Molecular and Nanoscale Interactions Governing Antibiotic Recognition on Graphene-Based Platforms

Graphene and its derivatives are primarily used for detecting antibiotics through an intricate relationship between molecular structure, surface chemistries, and nanomaterial design. This section combines knowledge about the physical/chemical characteristics of antibiotics with their behavior toward adsorption onto graphene surfaces, the ways in which advanced analytical tools can be used to understand the nature of the binding process, and how nanoparticles may be strategically employed as components of the sensor to enhance both sensitivity and specificity.

### 3.1. Adsorption Mechanisms on Graphene-Based Platforms

Many studies that have focused on the use of GO to detect antibiotics, including aminoglycosides (kanamycin [43]), have observed strong binding affinities between GO and the antibiotic and have attributed this binding to electrostatic interactions between the cationic form of the aminoglycoside (at low pH values) and the anionic form of GO. Additionally, studies examining the use of GO to detect penicillin and cephalosporin-type antibiotics have noted that the β-lactam ring carbonyl functional groups present on these molecules bind to the GO surface through hydrogen bonding between the carbonyl functional groups and the hydroxyl and carboxyl groups present on the GO surface [44,45]. Finally, studies utilizing GO that have attached antibodies or aptamers to GO surfaces using crosslinking agents such as EDC/NHS or silane chemistry [43,46] have demonstrated that the formation of stable covalent bonds between GO and the antibody/aptamer occurs.

The structure of the grapheme materials plays an important role in their ability to detect antibiotics. RGO is formed through chemically, thermally or electrochemically reducing graphene oxide and forms some of the original sp^2^ hybridized carbon network structure of graphite, with less oxygen and increased hydrophobic properties and electrical conductivity; this enhances π–π stacking of the aromatic domains of RGO with flat planar antibiotic structures. π–π stacking plays a vital role in detecting fluorinated quinolones (ciprofloxacin, levofloxacin, gatifloxacin [47,48,49]), tetracyclines (TC [50]) and nitrofurans/nitroimidazoles (nitrofurantoin [51]), which have extended conjugated ring structures. The π–π interaction in most RGO-based sensors serves as the first preconcentration method before being oxidized or reduced through electrocatalysis.

GR, whether chemical vapor deposition (CVD)-grown or exfoliated, has the greatest proportion of sp^2^ hybridized carbon and fewest functional groups; thus, it is best suited for non-covalent π–π interactions; however, its highly hydrophobic nature makes it poor for the adsorption of polar or charged antibiotics, except when functionalized [52,53]. Most importantly, the adsorption efficiency of the antibiotic will depend upon the pH of the solution. Specifically, aminoglycoside antibiotics (pK_a_~7–9) are polycations below pK_a_, adsorbed well on GO and poorly on RGO because of repulsion from remaining negative sites on RGO. Sulfonamides are anionic at neutral pH and are adsorbed better on cationic surfactant-modified RGO than unmodified RGO [54]. Fluoroquinolones are zwitterionic near physiological pH and form the best π–π stacks on RGO under mildly acidic conditions (pH 3–5). At these pH values, the piperazinyl ring of the fluoroquinolone is protonated, decreasing solubility and increasing surface adsorption [47]. Further, the adsorption of antibiotics onto graphene derivatives is also influenced by ionic strength and sample matrix (milk, urine, wastewater). High ionic strengths can screen electrostatic forces, and proteins and lipids can foul GO surfaces in biological samples [55]. Therefore, selecting the right graphene derivative based upon the target antibiotic’s structure and ionization profile is crucial to obtaining high-capacity adsorption, signal amplification, and selectivity in electrochemical biosensing.

### 3.2. Nanoscale Visualization and Characterization of Antibiotic Graphene Interfaces

Advanced analytical techniques have been used to study the physical and chemical interactions that occur when graphene-based nanomaterials interact with antibiotics in 94 studies examined in this systematic review. Scanning electron microscopy (SEM) imaging consistently revealed wrinkled porous structures in composites (β-CD/GO/MOF) that smoothed upon antibiotic adsorption, confirming preconcentration capabilities [56,57]. SEM imaging of the Au-Ag-ANCC/r-GO nanohybrid revealed the successful incorporation of the ANCCs into the r-GO matrix, creating a porous interconnected nanostructure that increased the electroactive surface area and the catalytic activity of the hybrid toward simultaneously detecting VAN and CEF residue levels [58]. In the FeVO/p-rGO sensor for furaltadone, Transmission Electron Microscopy (TEM) confirmed the 3D porous network of p-rGO with uniformly embedded FeVO nanoparticles; post-adsorption, electron-dense antibiotic clusters were observed on nanosheet surfaces, supporting adsorption-driven accumulation [59]. TEM verified uniform nanoparticle anchoring (MnO_2_ on GO) and crystallographic integration, enhancing charge transfer [60]. Atomic Force Microscopy (AFM) quantified surface roughness increases (25.59 pm^2^) correlating with sensitivity [60]. X-ray Photoelectron Spectroscopy (XPS) and Fourier-Transform Infrared Spectroscopy (FTIR) confirmed chemical states (Mo–S bonds in MoS_2_, Mn-O vibrations) and successful hybrid formation [60,61]. Collectively, morphology correlated with reproducibility, while electronic doping predicted signal enhancement.

### 3.3. Nanoparticle Integration for Enhanced Signal Transduction

In the 94 studies reviewed, metal, metal-oxide, bimetallic and metal organic framework nanoparticles (MOF NPs) increased the sensitivity of sensors by increasing electrical conduction, catalysis and binding affinity to detect trace amounts of structurally redox-silent antibiotics or those that are difficult to detect due to their low concentration.

#### 3.3.1. Gold Nanoparticles

Gold nanoparticles (AuNPs) were used in 28 research studies. In aptasensors for chloramphenicol [62], streptomycin [63], and ciprofloxacin [64], AuNPs have been used as stable surfaces for the binding of oriented aptamers that can lower electron transfer resistance. For example, the PEI-rGO/AuNCs CAP aptasensor [65] used Au nanocubes to increase the sensitivity of the signal produced by the sensor and improve the capture rate of target analytes and achieved an LOD at the femtomolar level (2.08 pM).

#### 3.3.2. Silver Nanoparticles

Silver nanoparticles (AgNPs) and silver-based nanocomposites (for example, Ag_2_Se [66]; Ag-Au nanocoral clusters [58]) provided dual functionality: catalytic enhancements and inherent antibacterial characteristics that were able to suppress biofouling. In addition, the Ag_2_S/rGO sensor for gatifloxacin [48] utilized Ag^+^ redox centers in order to catalytically oxidize the piperazine ring via a two-electron/two-proton oxidation process, resulting in a ten times greater signal amplification than that produced by RGO alone.

#### 3.3.3. Magnetic Iron Oxide Nanoparticles

Iron oxide nanoparticles with magnetic property (Fe_3_O_4_, γ-Fe_2_O_3_) have been studied in fifteen papers, especially for the detection of chloramphenicol (CAP) [67] and metronidazole [57]. They can reduce nitro groups as well as be used to enable magnetic solid phase extraction (MSPE). The MIO@NG/MSPE sensor [67] developed an external magnet to concentrate chloramphenicol from milk and separate it from protein/fat components, thus increasing the sensitivity of the method (LOD 0.01 µM) and the tolerance to the sample matrix.

#### 3.3.4. Metal Oxides

Metal oxides such as ZnO, TiO_2_ and MnO_2_ provided higher isoelectric points and surface hydroxyl groups, which further enhanced electrostatic interactions with charged forms of antibiotics. In the case of sulfamethoxazole, a GO/ZnO NRs sensor [68], ZnO nanorods were used as a spacer to preclude GO layering while enabling photo-induced charge separation to enhance the oxidation process. Similarly, MnO_2_/rGO composites improved the oxidation of ofloxacin by promoting a proton-coupled electron transfer [69]. A NiO/rGO platform was used to selectively detect linezolid by virtue of Ni^2+^/Ni^3+^ redox-mediated processes [70].

#### 3.3.5. Metal–Organic Frameworks

A number of hybrid modifiers based on MOFs were used in 12 studies. For example, Zr-based MOFs (for example, NH_2_–UiO-66/rGO) can detect copper indirectly by the reaction of the copper ions with the framework to displace a Cu^2+^ ion that is bound to a nitrogen atom [71]; likewise, Fe/Al-MOF/rGO can detect chloramphenicol (also known as chloroacetaminophen) by coordination bonding [72]. Co_2_CuS_4_-MOF/rGO/Au hybrids contain both porous MOF structures that allow for analytes to pass through and gold nanoparticle structures that enable plasmonic catalytic activity, which allows them to detect two types of analytes simultaneously in serum [73].

#### 3.3.6. Rare Earth Nanoparticles

Several rare earth-based nanoparticles (e.g., GdVO_4_ [74], SmMoSe_2_ [75], HV [76], Eu_2_O_3_ [77]) have been developed that include redox-active f-orbital sites and O-vacancy sites, which facilitate multi-electron transfer. The Eu_2_O_3_/rGO for chloramphenicol [77] demonstrated significant sensitivity (6.91 µA·µM^−1^·cm^−2^) as a result of the Eu^3+^/Eu^2+^ cycling process; however, these are typically expensive and difficult to synthesize.

## 4. Graphene-Based Sensors and Biosensors for Electrochemical Detection of Antibiotics

A bibliographic analysis reveals a coverage of 12 distinct antibiotic groups or 33 antibiotics. The antibiotics are separated only if they are detected individually by an electrochemical sensing study. The 2D structures derived from simplified molecular input line entry system (SMILES) notations from the respective PubChem CIDs of these antibiotics are presented in Figure S2 in the Supplementary Materials, along with their respective groupings. The sub-groups are presented in rounded rectangles, while distinct groups are presented in pointed rectangles. The antibiotics are selected based on either being represented in CARD’s antibiotic resistance ontology (ARO, 4.0.1) or chemical entities of biological interest (CHEBI, 246.0) for SMILES notations. The physicochemical properties of major antibiotic classes for electrochemical sensing on graphene-based platforms are mentioned in Table S1 in the Supplementary Materials.

### 4.1. Amoxicillin

A graphene-based sensor decorated with gold nanoparticles and modified with laccases enzyme (CVD Gr/AuNP/Lac) was developed by Osikoya et al. for the detection of Amoxicillin (AMX) in phosphate buffer solution (PBS) (10 pH) matrix using chronoamperometry (CA) [53]. AMOT recognition relies on Lac enzyme-mediated electrocatalysis, where phenolic oxidation at the enzyme’s copper-active site reduces Amoxicillin trihydrate (AMOT), generating a current proportional to its concentration. Specificity arises from strong AMOT adsorption onto the AuNP/GR surface. The sensor achieves a wide linear range (0.425–292 µM) and low LOD (0.425 µM). However, limitations include a complex, costly fabrication process, unverified performance in real samples, and the use of hazardous ammonium persulfate (APS).

### 4.2. Ampicillin

A graphene–alkyne-derived ampicillin aptasensor (GA-NH-YN) on a screen-printed carbon electrode substrate (SPCE) was developed by Flauzino et al. [78] for the detection of ampicillin (AMP) in tap water, milk and saliva using square wave voltammetry (SWV). Recognition relies on a DNA aptamer specific to AMP, immobilized via a copper-catalyzed azide–alkyne cycloaddition (CuAAC, “click chemistry”) between an azide-modified aptamer and an alkyne-functionalized graphene. The binding of AMP induces a conformational change that positions a methylene blue (MB) redox tag closer to the electrode, generating a “signal-on” response proportional to the AMP concentration. The sensor exhibits an exceptional linear dynamic range (0.01–10,000 µM) and an ultralow LOD (0.00136 µM), along with good stability. Despite promising smartphone integration and performance across complex matrices, critical limitations hinder practical deployment.

Cho et al. fabricated a Ca^2+^-doped MgAl_2_O_3_-G-SiO_2_ (MACGS) mesoporous sensor on a Ni foam substrate for electrochemical detection of azithromycin (AMN) [79] through cyclic voltammetry (CV). The mesoporous SiO_2_ increases the specific surface area and hydrophilic surface, which provides a high adsorption capacity for AMN, while GR and MAC improve the conductivity and catalytic and electrochemical properties of the composite. In another work [80], AMN has been measured by a graphene ribbon and ionic liquid nanocomposite consisting of CoFe_2_O_4_@NiO and 1-hexyl-3 methylimidazolium hexafluorophosphate (HMIM PF6) as an ionic liquid binder on a carbon paste electrode (CPE) substrate. The resulting CoFe_2_O_4_@NiO/HMIM PF6 was used for the determination of AMN in urine and pharmaceutical formulations using the SWV technique.

### 4.3. Chloramphenicol

**Figure 2:**
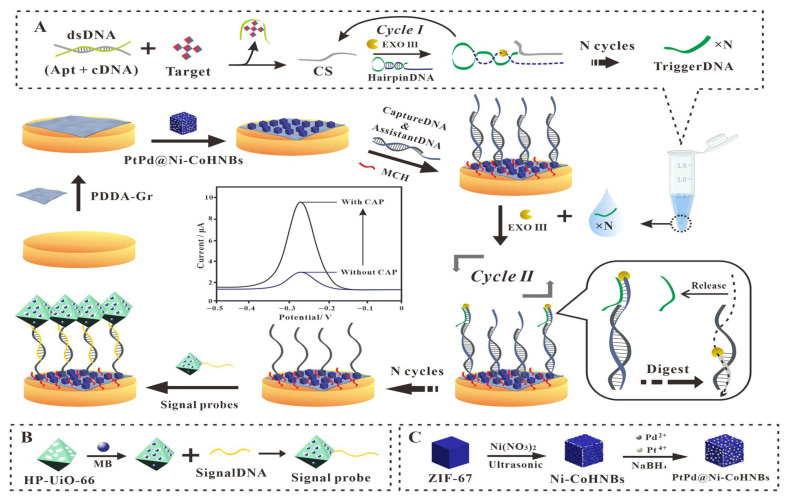
Signal amplification mechanism for ultralow chloramphenicol detection. (**A**) Exonuclease III-assisted cyclic amplification cycle showing how target binding triggers probe release and regeneration; (**B**) signal probe preparation process with thiol-modified DNA attachment to PtPd@Ni-Co hollow nanoboxes; (**C**) synthesis process of PtPd@Ni-CoHNBs, showing bimetallic nanoparticle formation on nickel–cobalt hollow structures (reproduced with permission from Ref. [81], copyright 2021 ACS).

A range of graphene-based electrochemical sensors has been developed for the detection of chloramphenicol (CAP) in food and biological samples, each demonstrating strong analytical performance, often with impressively low LODs, sometimes reaching the femtomolar range. These sensors employ diverse nanomaterials, including Zr-MOFs, MXenes, magnetic iron oxide, gold nanoparticles, and laser-induced graphene, combined with strategies like enzymatic signal amplification or direct electrocatalytic reduction. For instance, an aptasensor based on PtPd@Ni-Co hollow nanoboxes and PDDA-functionalized graphene achieved an ultralow LOD of 0.985 fM in honey (Figure 2) using Exonuclease III-assisted signal amplification [81]. As illustrated in Figure 2A, the cyclic amplification mechanism allows single-target molecules to trigger multiple signal events, explaining the femtomolar sensitivity. However, note the fabrication complexity (Figure 2C), which limits field deployment, as discussed in Section 8.4.

A portable magnetic iron oxide–nitrogen-doped graphene (MIO@NG) sensor offered ease of use for milk and eye drops but suffered from poor calibration reliability at low concentrations and required strict pH and temperature control [67]. Another system using Au nanoparticles on carbon nitride–graphene (Au/C_3_N_4_/GN) detected CAP in milk with an LOD of 0.027 µM but relied on toxic reagents (KMnO_4_, H_2_SO_4_) and a 550 °C calcination step, limiting scalability [82]. A lignocellulosic carbon/GO–MnO_2_ hybrid enabled direct CAP reduction with an LOD of 1.2 nM, yet retained only 84.6% of its signal after 20 cycles, raising concerns about long-term stability [60]. A flexible laser-induced graphene (LEG) sensor allowed on-site, label-free detection in milk and pork but exhibited a relatively high LOD (20 µM) and still required sample preparation [83]. Most recently, a Ti_3_C_2_T_x_ MXene electrode decorated with arginine/serine-functionalized graphene quantum dots and AuNPs achieved an exceptional LOD of 0.0012 µM, but demanded precise pH control and involved a multi-step synthesis [84].

### 4.4. Ciprofloxacin

Graphene-based electrochemical sensors for the determination of ciprofloxacin (CIP) have been synthesized and evaluated in various sample types, including environmental and biological, based on the oxidation of the piperazine ring of CIP [85,86]. The first approach utilized the combination of green-synthesized Fe_3_O_4_ nanoparticles and graphene nanosheets into a carbon paste electrode (GR NSs/Fe_3_O_4_/CPE). The GR NSs/Fe_3_O_4_/CPE was able to detect CIP in tap water, river water, and antibiotic plant effluent with a detection limit of 1.8 nM and a linear dynamic range of 0.01–20 µM by differential pulse voltammetry (DPV) [86]. However, the electrochemical properties of this sensor are very sensitive to pH and the quality of the electrolytes prepared for each measurement, which can lead to inconsistencies in results when evaluating the sensor in complicated real-world matrices. Another graphene-based electrochemical sensor utilized sodium dodecyl sulfate (SDS) with graphene on a carbon paste electrode (SDS/Gr/CPE) to improve electron transfer during CIP oxidation in urine and pharmaceutical tablets [85]. However, this sensor is limited by the requirement to add fresh SDS to the electrode and re-pack the electrode for each measurement, which is impractical for field applications or continuous measurements. A third design utilizing electrochemically exfoliated graphene on glassy carbon (EGr/GCE) has also been described for the determination of CIP in wastewater and pharmaceutical products using both linear sweep voltammetry (LSV) and CA [52].

### 4.5. Florfenicol

A graphene–copper phthalocyanine nanocomposite-modified glassy carbon electrode (GR/CuPc NCs/GCE) was developed for the simultaneous detection of CAP and florfenicol (FF) in milk and eye drops using DPV [87]. The electrochemical mechanism of this sensor is based upon the electrocatalytic reduction of nitro groups in CAP and methyl sulphonyl groups in FF. In the process, the CuPc reduces the overpotential required for the reduction of the nitro and methyl sulphonyl groups, while the graphene provides an enhancement of the electrical conduction, which leads to the enhancement of the sensitivity of the sensor. The main drawbacks to the application of the described method are the need for very rigid control of the pH of the sample solution, the requirement of anaerobic conditions through the use of nitrogen gas degassing during DPV measurements, and the relatively small linear dynamic range of the sensor, which would limit the ability to detect varying levels of FF and CAP in diverse real-world samples.

### 4.6. Kanamycin

Two electrochemical biosensors were fabricated to detect kanamycin (KAN). Both used a combination of graphene or carbon nanomaterials as a substrate for nucleic acid recognition elements. The first one was based on an ssDNA probe that was attached to a graphene/CdS/chitosan nanocomposite (ssDNA/GR-CdS-CHIT/GCE). This biosensor functioned through a “signal off” method where the presence of KAN caused the ssDNA probe to bind to KAN, causing the guanine bases within the ssDNA to become masked and reducing the amount of current produced by the oxidation of the guanine bases [88]. The other biosensor used an aptamer on mesoporous carbon screen-printed electrodes (OMC/Aptamer/SPCE) to measure multiple aminoglycosides (KAN, neomycin, gentamicin) in milk [43]. The aptamer in the biosensor prevented the target from interacting with the redox probe, thus preventing the redox probe from accessing the target and increasing the charge transfer resistance associated with the target. The LOD for this biosensor was low (2.47 nM), and the biosensor was portable. The ssDNA-based biosensor was susceptible to non-specific binding [88], and the aptamer-based biosensor could not distinguish between structurally similar aminoglycosides [43].

### 4.7. Metronidazole

The research team of Zokhtareh et al. created a glassy carbon electrode that was modified with iron oxide/graphene nanocomposites as an electrochemical sensor for detecting metronidazole (MNZ) in environmental water samples via DPV [89] and found that the sensitivity of the sensor allowed the detection of MNZ at very low concentrations. The electrochemical reduction of the nitro group in MNZ takes place through a four-electron/four-proton process, and the presence of graphene increases the electrical conductivity of the electrode while the presence of the Fe_3_O_4_ nanoparticles provides catalytically active sites.

### 4.8. Nitrofurantoin

The LuVO_4_/graphene-modified glassy carbon electrode (LuVO_4_/GRS/GCE) has been developed as an amperometric detector of nitrofurantoin (NFT) in environmental water samples through the catalytic electroreduction of the nitro group of NFT (4e^−^/4H^+^) [90]. The LuVO_4_/graphene-modified glassy carbon electrode demonstrated very good analytical characteristics: high sensitivity of detection with an LOD of 1 nM and a wide dynamic linear range from 0.008 to 256 µM with nearly quantitative recovery of NFT. However, despite these excellent analytical properties, the LuVO_4_/graphene-modified glassy carbon electrode has significant practical limitations: the LuVO_4_/graphene-modified glassy carbon electrode relies upon lutetium, a rare earth element whose cost and availability are major concerns for the development of a commercially viable environmental sensing system.

### 4.9. Meropenem

An example of an electrochemical sensor platform that can be used to detect linezolid (LZD) and meropenem (MPM) was developed by the research group of [91]. This includes two layers of casted CNT-HQ mixture with nanoparticles of an Fe-Ni alloy on the GC/Gr/CNT-HQ/Fe-Ni electrodes for the simultaneous detection of LZD and MPM in human blood and serum samples utilizing both DPV and CV techniques. The major drawback to the practical use of this sensor is its narrow linear dynamic range; it is strictly dependent upon pH, and most notably, the production of the sensor requires a multi-step process of fabricating a layered nanocomposite by casting. Although the sensor does exhibit good stability and functionally operates within biologically relevant matrices, these are constraints, especially the pH dependence and the complexity of modifying the electrodes.

### 4.10. Ornidazole

Venkatesh et al.’s work (2021) used manganese molybdate and graphene nanosheets on a glassy carbon electrode (MnMoO_4_/GNS/GCE) sensor to detect ornidazole (ORN) in river water and human urine with an amperometric method [92]. The platform that was developed is portable, needs sample preparation, and the composite made from nanostructure has a complex formation, which can be unstable when the conditions are not precisely controlled. Furthermore, although there was high sensitivity (94.6% of the original signal) for only about 2500 s of operation time, it still would not provide reliable results for on-site and/or continuous monitoring.

### 4.11. Sulfamethazine

The “gate effect” sulfamethazine (SMZ) sensor based on multiwalled carbon nanotubes/graphene quantum dots/molecularly imprinted polymer (on a GCE substrate) for detecting SMZ in aquaculture seawater uses “signal off” as a method for sensing the presence of SMZ. In this method, when SMZ binds to the molecularly imprinted cavities, it blocks the redox probe from accessing the electrode surface, causing a measurable current drop [93]. Although the concept of the sensor is elegant, there are several issues that affect the sensor’s ability to accurately sense SMZ. First, the molecularly imprinted polymer (MIP) is synthesized through a very time-consuming process involving the use of templates that must be carefully removed to produce a sensitive sensor. This process is easily contaminated with unremoved template material, which will negatively impact both the sensitivity and the reproducibility of the sensor. Further, the sensor has not been validated using environmental samples such as those found in seawater and has been demonstrated to be stable for no longer than five days.

### 4.12. Sulfamethoxazole

The paper-based rGNR/parchment sensor is capable of providing both portable and simultaneous detection of sulfamethoxazole (SMX) and trimethoprim in tap water by controlling electrochemical oxidation through adsorption. This is due to the edge-rich structure of reduced graphene nanoribbons that provide distinct DPV peaks and an extremely low limit of quantification (LOQ) for SMX at 0.04 μM [94]. The multiplexing and portability capabilities of this sensor are significant advantages; however, there are also several limitations associated with this sensor, including a relatively narrow linear range (1–12 μM for SMX). The recovery levels of the sensor can be highly variable (as low as 54.9%), and the sensor has no reported stability. These limitations raise serious concerns regarding possible matrix effects and loss of analytes during sampling. Additionally, the mechanical fragility of the parchment substrate used as the base of the sensor may cause mechanical failure in various environmental conditions. Table 1 summarizes the analytical characteristics of the graphene-based sensor platform.

## 5. Graphene Oxide-Based Sensors and Biosensors for Electrochemical Detection of Antibiotics

While pristine graphene offers excellent conductivity, its hydrophobic nature limits interaction with polar antibiotics. GO, with its oxygen-rich surface chemistry, addresses this limitation, as discussed in this section.

### 5.1. Benzylpenicillin

Nourbaksh-Amiri and Najafpour-Darzi developed a GO/MnFe_2_O_4_-modified carbon paste electrode for the measurement of benzylpenicillin (Pen-G) in water through the application of DPV [45]. However, the calibration plot obtained using this sensor showed two linear ranges, which implies a nonlinear relationship between the concentration of Pen-G and the peak current measured by the sensor. This could be a significant limitation to the practical use of this sensor for real-time measurements of Pen-G levels in drinking water. Aptasensors are sensors that utilize nucleic acid sequences known as aptamers that selectively bind to specific molecules or ions. A GO-based aptasensor has been reported to measure multiple types of penicillins in milk using an aptamer with a very broad specificity toward penicillins (P-11-1) [78]. Although the sensitivity of this sensor is significantly higher than most previously reported biosensors for the detection of penicillins, it relies upon the use of gold nanoparticles (AuNPs), which are expensive and require a multi-step fabrication process.

### 5.2. Cefixime

A graphene oxide/gold nanowire (GO/GNW) nanocomposite and an electropolymerized molecularly imprinted polyaniline (EMIP) film for DPV-based detection of cefixime (CFM) in both human serum and urine was developed [44]. This sensor utilizes the “lock-and-key” molecular imprinting mechanism as follows: the electropolymerization is performed using CFM as a template; then, the CFM is removed, and a cavity that is specific to the shape of the CFM remains in the film. However, the process of developing this sensor is time-consuming, expensive due to the use of nanomaterials, and has a high risk of defects, primarily from either incomplete removal of the template or a non-uniform application of the polymer film, resulting in low reproducibility and low scalability.

### 5.3. Cephalexin

Kolhe et al. have created a very sensitive immunosensor for the antibiotic cephalexin (CFX), using antibodies against CFX conjugated with BSA-antibodies and then attaching them to GQDs placed on an FTO electrode [47]. The reduction in DPV current is a function of the amount of CFX present. The DPV current is caused by the steric hindrance of the antibodies when they bind to the antigens, which prevents the redox probes from passing through. As a result of the high electrical conduction of GQDs, the LOD was 0.53 fM, and the linear range of this sensor was 1 pM–1 μM. However, because of the complexity and cost associated with the fabrication process, as well as the fact that it uses materials dependent upon animals (i.e., antibodies), the potential application of the sensor is limited. Furthermore, the low durability of the sensor will also limit its scalability and reusability.

### 5.4. Chloramphenicol

**Figure 3:**
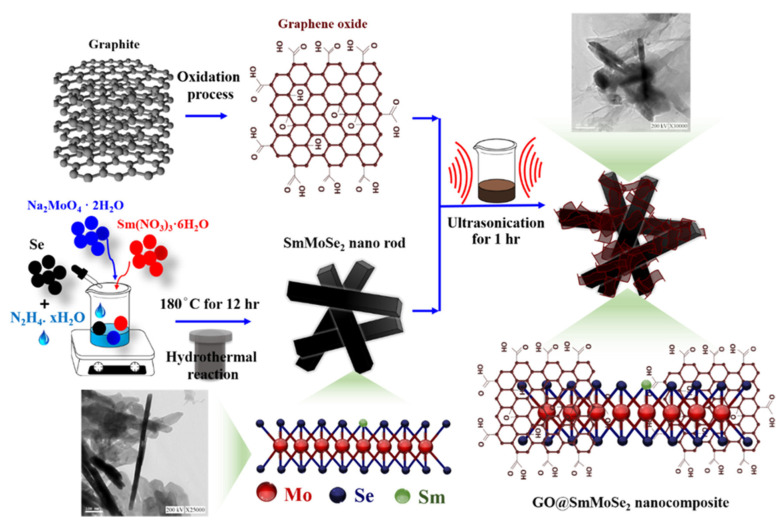
Synthesis and sensing mechanism of GO@SmMoSe_2_ nanocomposite for chloramphenicol detection. The diagram shows: (i) hydrothermal synthesis of SmMoSe_2_ nanoparticles; (ii) GO nanosheet functionalization; (iii) nanocomposite assembly on electrode surface; (iv) electrochemical detection mechanism showing Sm^3+^/Sm^2+^ redox cycling for signal amplification (reproduced with permission from Ref. [75], copyright 2018 ACS).

Numerous graphene oxide (GO)-based electrochemical sensors have been developed for CAP detection across food, clinical, and environmental matrices, leveraging the irreversible four-electron reduction of CAP’s nitro (–NO_2_) group to hydroxylamine. Platforms include GO/MWCNT, GO/ZnO, and GO/SrTiO_3_ on glassy carbon electrodes (GCE) for CAP analysis in milk, honey, serum, and water using CV, DPV, or LSV [96,97,98]; a MoS_2_–ionic liquid/GO nanocomposite for urine and milk with an LOD of 0.047 µM [99]; and GCE sensors modified with SmMoSe_2_ (Figure 3), GdSnO, or Pd nanoparticles, which also exploit a secondary redox couple for CAP fingerprinting [55,75,100]. As shown in Figure 3, the Sm^3+^/Sm^2+^ redox cycling provides signal amplification beyond standard carbon-based sensors, achieving 20.6 µA·µM^−1^·cm^−2^ sensitivity. However, the rare earth material cost limits economic viability, as per Section 8.6. Additionally, GO–MoS_2_ hybrids on screen-printed electrodes achieved LODs of ~0.06–0.07 µM in meat samples but required careful synthesis control and sample pretreatment [101]. These nanocomposites enhance surface area, electron transfer, and electrocatalysis, yielding LODs from 0.007 to 6.08 µM and linear ranges up to 522 µM, with GO/MWCNT offering the lowest LOD and the only reported portable implementation [96] and GO/GdSnO delivering the broadest LDR [55].

### 5.5. Cloxacillin

The research group of Golkarieh et al. developed an electrochemical sensor based on gold nanorods and graphene oxide (AuNR/GO) deposited on a SPCE for the detection of cloxacillin (CLOX) in aqueous solutions using DPV [102]. The working mechanism of the sensor is through electrocatalytic oxidation, in which GO is used as a high-surface-area scaffold that facilitates the pre-concentration of the analyte, while AuNRs are utilized as electron transfer mediators that amplify the oxidation current generated due to CLOX concentrations. Furthermore, sensors have shown a limited life span of approximately 12 days, and the researchers have not provided a method for the regeneration of the sensor, resulting in a lack of reusability of the sensor and increased costs associated with the extended use of the sensor.

### 5.6. Furazolidone

A graphene oxide–cerium-doped tungsten oxide (CeW/GO) nanocomposite was developed as an electrode for the DPV-based detection of furazolidone (FUZ) in human urine [103]. The sensor used the porosity of the coral-like structure of CeW/GO to efficiently adsorb FUZ through hydrogen bonds; also, the addition of cerium enhanced the electrocatalytic reduction of the nitro group of FUZ, leading to a signal dependent on the concentration of FUZ, and provided an LOD of 0.054 μM and a linear range of 1–260 μM. However, the practicality of this sensor has several drawbacks, including long response time, necessary sample pre-treatment, and, most importantly, variable calibration at lower concentrations, which reduces both the reproducibility and reliability of results.

### 5.7. Levofloxacin

The CNNS/GO-modified GCE sensor was developed by the research group in [104] to detect both acetaminophen and levofloxacin (LEV) simultaneously using DPV in river water. The nanocomposite increases the surface area and increases the rate of electron transfer, which produces a clear, sharp LEV oxidation peak at +0.94 V. The sensor is able to detect as low as 0.079 µM of LEV and has a very small linear range (0.5–15 µM). The ability of the sensor to detect multiple substances allows it to be multiplexed; however, the use of this sensor is limited in many ways, including long response time (480 s), non-portable nature, and the absence of validation and long-term stability in complex environmental samples.

### 5.8. Sulfadiazine

The development of a GdVO_4_@GO-modified glassy carbon sensor was fabricated for the amperometric determination of sulfadiazine (SDZ) in both human serum and water [74] via the electro-catalytically oxidized aromatic amine groups on SDZ (2e^−^/2H^+^). Due to the enhanced conductivity of the nanocomposite, the prevention of GO restacking, and lowered oxidation potential, the LOD of this sensor was found to be as low as 0.00319 µM with a broad linearity of 0.02–945.5 µM. However, the high cost of rare earth elements (GdVO_4_), susceptibility to biofouling in complex matrices, and calibration non-uniformity are constraints.

### 5.9. Sulfamethazine

A Cu-Ag core-shell-decorated GO/GCE was made for the detection of SMZ in cow’s milk using SWV [105]. The nanocomposite enables an irreversible oxidation of SMZ’s aromatic amine group by diffusion control, where the Cu-Ag nanoparticles function as “electron highways” to enhance both conductivity and catalytic activity, while GO provides very high adsorption capacity. The Cu-Ag/GO/GCE has a low LOD and a relatively broad dynamic linear range and recovery (~100.3%). Nevertheless, the sensor is prone to fouling when in contact with a complex milk matrix and uses costly noble metals, which require a multi-step synthesis process.

### 5.10. Sulfamethoxazole

Two GO-based electrochemical detection systems for SMX were reported: a system developed by researchers [54,68] employing GO/ZnO nanorods on a GC electrode, and a portable system developed by other researchers [54] utilizing ascorbic acid-reduced GO with cetyltrimethylammonium bromide (CTAB) on an SPE. The GO/ZnO sensor was based on π–π stacking, H-bonding, and photo-enhanced charge transfer to produce a very low LOD (0.281 nM). Additionally, the formation of uniform composites presented challenges during fabrication. The AA-RGO/CTAB/SPE demonstrated acceptable portability and relatively good sensitivity through the combination of electrostatic pre-concentration (via CTAB) and π-π interactions; however, this sensor’s performance was highly pH-dependent, CTAB is desorbed in dynamic environments, and the authors did not report on key metrics such as reproducibility, repeatability or long-term stability.

### 5.11. Sulfathiazole

An ultra-sensitive electrochemical nanosensor based on a holmium vanadate–graphene oxide nanocomposite (HV@GO/GCE) was developed for the electrochemical determination of sulfathiazole (STZ) in river water and urine using CV and DPV [76]. The working principle of this nanosensor utilizes the redox centers of HV nanoparticles for highly efficient 2e^−^/2H^+^ electrochemical oxidation of STZ, while GO improves the surface area, inhibits the aggregation of HV nanoparticles and promotes electron transfer, leading to a very low LOD and a wide linear response range. However, the application of this nanosensor under real conditions is limited due to the very high price and rare availability of holmium, as well as its sensitivity to fouling by organic and urinary components, relatively short operating life, and non-uniform calibration.

### 5.12. Tetracycline

There are three sensors for tetracycline (TC) that were developed using GO which each have high sensitivity but many practical limitations. The first sensor was a “signal off” sensor based on MIP/GO/Al_2_O_3_/Au developed by Zhao & Zhao. This sensor had the lowest LOD, but it had a very narrow range of linearity. The template was also not completely removed during the fabrication process and this sensor is susceptible to fouling by both milk and feed matrices [106]. Another sensor (AuNPs/GO/TC-MIP/GCE) allowed TC to be oxidized directly, and this sensor had an LOD of 0.00157 µM. However, this sensor had many limitations, including the stability of the MIP layer, variability in the electropolymerization of the MIP layer between batches, and long-term reliability; however, this sensor did provide recoveries in the middle of the range [107]. The most innovative sensor was a microfluidic solar-powered sensor (CA-CNTs/CuS-GO), which used sunlight to evaporate water and perform preconcentration and detected TC at concentrations as low as 0.000159 µM [108]. However, this sensor’s performance depends on the amount of ambient light and humidity, and the chip is easily fouled by organic matter.

### 5.13. Trimethoprim

A GO–ZnO quantum dot-modified GCE (GO–ZnO QDs/GCE) was prepared for the simultaneous detection of trimethoprim (TMP) and SMX by using an SWV method in pharmaceutical tablets, utilizing ZnO’s nanozyme catalytic activity and GO’s conductive properties at an LOD of 0.0658 μM for TMP [109]. Although this sensor allows for the dual analyte detection of TMP and SMX with high selectivity in simple matrices, it is limited in validation and untested for interference and reliability in complex biological samples. Furthermore, ZnO QDs are dissolved under acidic or basic conditions, which is a limitation for the long-term stability of the sensor. Table 2 summarizes the analytical characteristics of the graphene oxide-based sensor platform.

## 6. Reduced Graphene Oxide-Based Sensors and Biosensors for Electrochemical Detection of Antibiotics

### 6.1. Amoxicillin

Reduced graphene oxide (RGO)-based electrochemical sensors show promise for detecting AMX, leveraging rGO’s conductivity combined with catalytic nanomaterials such as RuO_2_ nanoparticles (rGO/RuO_2_ NPs) or NiAl-layered double hydroxides (NiAl-LDH/rGO) to enhance sensitivity and selectivity [112,113]. Both platforms enable the simultaneous detection of AMX with other analytes (tetracycline or ethinylestradiol) in complex matrices like tap water or urine using the DPV technique, achieving low limits of detection and wide linear ranges. However, the NiAl-LDH/rGO has a main challenge: the degradation of tetracycline that occurs when this system operates at a certain pH level, especially due to increased protonation effects caused by Nafion filtration [112]. Second, there is no information about the long-term stability and reproducibility of this system. On the other hand, although the rGO/RuO_2_ NP sensors have demonstrated one-month stability with 82% retention of the signal intensity [113] they were able to detect, these sensors involve the expensive ruthenium and require a longer detection time.

### 6.2. Azithromycin

Santhan et al. developed an rGO-based electrochemical sensor (Ag_2_Se/β-CD/rGO/GCE) for AMN detection, utilizing host–guest complexation between β-cyclodextrin (β-CD) and AMN to achieve high selectivity, while Ag_2_Se nanoparticles lower the oxidation overpotential and rGO offers a conductive, high-surface-area scaffold for efficient electron transfer [66]. The sensor demonstrates excellent performance, a wide linear detection range (0.023–971.7 µM), and an ultralow LOD (0.0045 µM) via the irreversible electro-oxidation of AMN involving demethylation of the macrolide ring. However, despite its sensitivity and rapid fabrication, the requirement for sample pre-treatment limits its suitability for on-site or continuous environmental monitoring.

### 6.3. Benzylpenicillin

Sahoo et al. developed a chemically reduced graphene oxide (crGO)-based electrochemical sensor functionalized with a 35-mer amine-modified DNA aptamer (ssDNA-A2) for Pen-G detection in milk, meat, and eggs using DPV [114]. The sensor operates via a “signal-on” mechanism: Pen-G binding induces aptamer conformational change, lifting it from the electrode surface and enabling enhanced electron transfer with the redox probe, yielding a current proportional to Pen-G concentration. It achieves an exceptional LOD of 1.24 pM and a broad linear range (1 pM–1 µM). However, its practical utility is limited by costly and time-intensive fabrication, poor recovery in complex matrices like eggs, mandatory sample pre-treatment, lack of portability, and absence of real-time capability.

### 6.4. Ceftriaxone

Ceftriaxone (CEF) has been detected using RGO-based sensors in surface water, chicken, fish and milk samples [58,115]. Uddin et al. developed a MoS_2_–RGO nanocomposite sensor for the electrochemical detection of CEF in surface water using DPV, leveraging MoS_2_’s electrocatalytic sites and RGO’s high surface area to facilitate CEF oxidation via a proton-coupled electron transfer (PCET) mechanism involving the primary amine and subsequent sulfur oxidation in the aminothiazole ring [115]. This yields a distinct, pH-dependent peak potential (−28.66 mV·pH^−1^) at ~0.792 V (pH 2), enabling selective detection without sample pre-treatment—a notable practical advantage. However, the sensor’s LOD (0.28 µM) and linear range (1–100 µM) are less competitive compared to state-of-the-art platforms, and the lack of comprehensive interference studies in complex real-world matrices (milk, fish, chicken) limits its reliability and broader applicability.

### 6.5. Chloramphenicol

Numerous rGO-based sensors for CAP detection employ diverse strategies (e.g., 3D rGO architectures, metal/metal-oxide nanocomposites, polydopamine-gold hybrids, MOF-derived architectures, and aptasensors); however, they share critical limitations [62,65,72,73,116,117]. While these platforms leverage rGO’s conductivity and high surface area enhanced by dopants (Cl-RGO) [118], metals (Au, Sn, Fe_3_O_4_) [73,116,119] or porous MOFs (Fe/Al-MOF/rGO, ZIF-8-derived carbon) [72,120] to catalyze the nitro-group reduction of CAP and achieve low LODs (as low as 0.00132 µM for Eu_2_O_3_/rGO [77] or 2.08 pM for an aptasensor [65]), they share critical limitations. Most require extensive sample pre-treatment, lack real-time or portable functionality, and exhibit restricted linear ranges or slow response times, hindering field-deployable environmental monitoring. Additionally, many rely on costly nanomaterials (AuNPs in rGO@PDA@AuNPs [62] or rGO/Au/Co_2_CuS_4_ [73]). Scalability is further compromised by precious-metal dependence and limited long-term robustness, as seen in the PEI-rGO/AuNCs aptasensor, which shows only 90-day stability [65].

### 6.6. Ciprofloxacin

rGO-based electrochemical sensors for CIP detection demonstrate high sensitivity, often achieving low LODs (0.7 nM to 0.078 µM) through strategies such as π–π stacking, host–guest inclusion (β-CD), aptamer binding, or metal/MOF-enhanced catalysis, but consistently face critical limitations that hinder real-world deployment. Another common problem associated with these platforms is their narrow linear ranges of detection (0.5–20 µm for m-RGO [47] and 0.02–1.0 µm for NH2–UiO-66/RGO [71]), which limit the quantitative analysis at environmentally relevant concentrations. Most of these platforms require multi-step, lengthy synthesis processes using harmful chemicals (hydrazine [121]) or expensive nanomaterials [122,123] to create them on an industrial scale. Moreover, the electrode fouling, the lack of specificity of the developed systems when tested against structurally similar antibiotics (fluoroquinolones) (enrofloxacin [124]), and the lack of validation studies in complex matrices all contribute to the low reliability of the developed platforms. While several of the sensor systems avoid the need for pre-concentration (Pt-RGO [123]), most of the other sensor systems require a sample treatment process and long detection times (EIS assay: 40 min [64]).

### 6.7. Furaltadone

In [59], a sensor utilizing an iron vanadate and porous reduced graphene oxide nanocomposite on a rotating ring disk electrode (FeVO/p-rGO NCs/RRDE) was fabricated for the detection of furaltadone (FLD) in industrial wastewater and lake water using amperometry (i-t Amp). Its operation is based on the electrocatalytic reduction of the nitro group in furaltadone to a hydroxylamine group. The 3D porous structure of the p-rGO offers a high surface area and efficient electron transfer pathways, while the mixed valence states (Fe^2+^/Fe^3+^ and V^4+^/V^5+^) in the FeVO nanoparticles provide rich catalytic sites for this reduction, simultaneously enhancing the sensitivity. The sensor demonstrates a wide LDR performance of 0.5–1319 µM and an LOD of 0.138 µM. Despite this achievement, the sensor’s design with an RRDE makes it non-portable and unsuitable for field use. The overall analysis time is slow, even though the accumulation is fast, as the protocol requires additional sample preparation, such as filtering and centrifugation. Additionally, the non-uniform calibration could lead to reproducibility issues, leading to batch-to-batch variation. Finally, the operational stability of 3500 s may be insufficient for long-term monitoring applications.

### 6.8. Furazolidone

An rGO–MXene nanocomposite modified screen-printed carbon electrode (rGO-MXene-PDDA/SPCE) was developed for the electrochemical detection of FUZ in tap and river water using DPV, leveraging the synergistic conductivity of rGO and MXene and a PDDA-mediated “brick-and-mortar” architecture that prevents restacking and maximizes active surface area for efficient nitrofuran group reduction [125]. The sensor achieves an excellent LOD of 0.275 nM and benefits from the inherent portability of the SPCE platform. However, it still requires sample dilution with buffer, limiting true on-site applicability, and exhibits a moderate linear range (0.2–78.2 µM). The main limitations of this sensor are the time to analyze samples and long-term stability.

### 6.9. Gatifloxacin

The Ag_2_S/rGO/GCE sensor has been developed to detect gatifloxacin (GAT) in fish and meat products through electrochemical oxidation at low concentrations and in wide ranges. This is due to the synergy developed when the Ag_2_S particles are combined with the rGO material to create a conductive support structure [48]. Although the Ag_2_S/rGO/GCE sensor has good sensitivity and a wide dynamic range, there are several limitations that limit its applicability. The Ag_2_S/rGO/GCE is not portable because it uses an inexpensive and readily available glassy carbon electrode. In addition, prior to measuring the amount of GAT in the samples, a multi-step process is required, involving the extraction of the samples into a mixture of formic acid and acetonitrile, followed by centrifugation, spiking, and the adjustment of the pH to 4.0 using an acetate buffer solution. These steps introduce complexity and possible inconsistencies in the measurements obtained in actual food product matrices.

### 6.10. Kasugamycin

A fMWCNTs/rGO/poly(SER)/GCE sensor was developed for kasugamycin (KAS) detection in vegetables via a signal-on electrocatalytic reduction mechanism, where poly-serine enables selective preconcentration through hydrogen bonding and the fMWCNTs/rGO composite enhances electron transfer, yielding an LOD of 0.96 µM and a linear range of 7.22–255 µM [126]. Despite good reproducibility and recovery in spiked samples, the sensor’s utility for food safety is limited by its non-portable GCE format, mandatory sample preparation, and a relatively high LOD that may be insufficient for trace-level monitoring.

### 6.11. Levofloxacin

An rGO-modified 3D-printed carbon black/PLA electrode (rGO/3D-CB/PLA) was developed for LEV detection in pharmaceuticals, synthetic urine, and tap water, leveraging rGO’s ability to lower the oxidation overpotential of LEV’s piperazinyl group by 120 mV and enhance signal via improved conductivity and surface area [49]. While the sensor benefits from a low-cost, portable, and disposable 3D-printed platform, its analytical performance is constrained by a relatively high LOD (2.17 µM) and narrow linear range (10–50 µM), limiting sensitivity for trace-level analysis, in addition to the challenges of analysis kinetics and the time required for sample preparation.

### 6.12. Linezolid

The NiO/rGO/PtE electrochemical sensor demonstrated low limits of detection for LZD in both urine samples and pharmaceutical tablet samples, with an exceptional LOD of 3.1 nM and a broad LDR. The combined effects of the NiO nanoparticles and the improved movement of electrons through the rGO enable the sensor to detect LZD at very low levels [70]. Although the NiO/rGO/PtE sensor has demonstrated rapid response time and superior analytical performance, the clinical or field application of this sensor is limited by the non-portable design, expensive platinum electrodes and nanocomposite material used in its construction, and the requirement for sample treatment prior to analysis.

### 6.13. Metronidazole

There were several rGO-based electrochemical sensors fabricated to detect MNZ that are highly sensitive but have limitations in practice [57,127,128]. The FeMn2O4-rGO/GCE sensor has limited portability, a long analysis time, and no sufficient validation for use in complex biological matrices. While the LOD was 12.9 nM, the linear range was 0.01–512.2 μM, and it utilized nitro group reduction [57]. On the other hand, a screen-printed C60/rGO/Nafion sensor had better portability and a faster response than the FeMn2O4-rGO/GCE sensor, but required pre-treatment of samples and had an elevated LOD of 0.21 μM, which reduces the utility of this device for point-of-care testing [127]. In addition to the screen-printed C60/rGO/Nafion sensor, the tannic acid-stabilized Au-Ag Alloy/rGO/poly(glycine) GCE sensor showed superior sensitivity in the presence of food and meat samples [127]; however, its complicated synthesis process and requirement for expensive noble metals limit the ability to scale up this technology or deploy it in the field.

### 6.14. Nitrofurantoin

**Figure 4:**
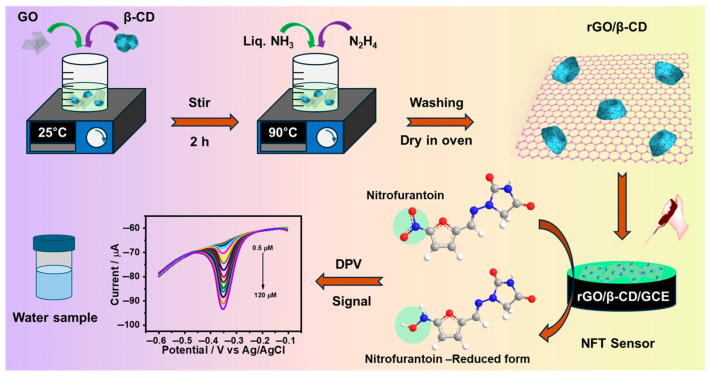
Host–guest chemistry mechanism for nitrofurantoin preconcentration on rGO/β-CD nanocomposite. The schematic displays: (i) β-cyclodextrin functionalization of rGO surface via esterification; (ii) host–guest inclusion complex formation between the β-CD cavity and nitrofurantoin’s aromatic ring; (iii) electrochemical reduction of the nitro group (4e^−^/4H^+^) with signal amplification; (iv) selectivity mechanism showing steric exclusion of larger interferents (reproduced with permission from [51], copyright 2025 MDPI).

An rGO/β-CD/GCE sensor has been fabricated for the detection of NFT in wastewater using the host–guest chemistry of β-cyclodextrin for analyte pre-concentration [51]. Figure 4 illustrates the inclusion complex mechanism where nitrofurantoin’s planar aromatic structure fits within β-CD’s hydrophobic cavity, enabling 10-fold preconcentration before electrochemical detection. This represents a cost-effective alternative to biorecognition elements, as discussed in Section 8.1.

### 6.15. Ofloxacin

The two rGO-based sensors for ofloxacin (OFX) are able to provide acceptable analytical performance; however, they each have significant practical problems [69,129]. The Zn_2_SnO_4_/rGO/Nafion/GCE sensor has an excellent LOD (0.0828 µM) based upon the electrostatic concentration of the analyte and 2e^−^/2H^+^ oxidation; it is limited by its relatively narrow linear range (0.0999–6.62 µM). Also, this sensor is not portable; analysis times are long, which reduces the applicability of this sensor for environmental use [129]. On the other hand, the AgNPs/MnO_2_/ErGO/GCE sensor provides simultaneous analysis in both water and pharmaceutical samples over a wider LDR (0.5–170 µM); however, the LOD (30 µM) is much higher than the LOD for the Zn_2_SnO_4_/rGO/Nafion/GCE sensor. Additionally, the response time of the AgNPs/MnO_2_/ErGO/GCE sensor is slow (500 s), the sensor is degraded after 7 days, and the fabrication process is complicated [69].

### 6.16. Rifampin

The MoSe_2_/rGO/β-CD/GCE sensor was developed to detect rifampin (RIF; also known as Rifampicin) in a variety of biological and environmental matrices at very low levels due to quasi-reversible 2e^−^/2H^+^ electrocatalytic processes, as well as through the effects of a preconcentration step involving host–guest complexation. A low LOD (2.8 nM) and a wide linear range (0.019–374.5 µM) were obtained with this sensor [130]. Although it provides good analytical performance, the sensor has limitations that make it unsuitable for POC.

### 6.17. Streptomycin

The RGO/AuNPs/aptamer-based sensor was developed on a PGE for the determination of streptomycin (STR) at low concentrations with an exceptionally low LOD (0.8 pM) [63]. It has several major disadvantages that limit its usability in practice: It cannot be used as a portable system due to its fixed electrode configuration and large size. The electrode can become fouled during the analysis of complex sample types such as milk. Due to the requirement of a multi-step process, including the precise immobilization of the aptamers onto the surface of the gold NPs and the high costs associated with AuNPs, the development process is also expensive and time-consuming.

### 6.18. Tetracycline

The number of rGO-based sensors for the detection of TC has grown significantly over the last few years, with high sensitivity (LODs ranging from 0.00046 μM to 30 pmol/L). However, all of these sensors have limited use in real-world applications [50,131,132]. The approaches used to improve selectivity (for example, using an adsorptive stripping sensor ERGO/SPE [50] or to amplify signals (such as those based on molecularly imprinted polymers LIG/AuNPs/MIP [131] or on aptamers (AuNPs/RGO/Apt/PGE [132] Apt/Fe/Zn-NaMMT/MWCNTs/GCE [111]) are affected by similar difficulties: low specificity when there are many analytes present in the same sample (cross-reactivity among tetracycline analogues [112]); susceptibility to fouling by biological material and interferences due to the matrix of milk or meat; and the necessity of extensive treatment of the sample before analysis, which results in loss of the analyte. In addition, the vast majority of the sensors reported so far show limited stabilities; the fabrication of most of the reported sensors is relatively complicated and expensive (for instance, AuNPs and laser-induced graphene); and the sensor configuration is usually non-portable or confined to laboratory settings.

### 6.19. Tobramycin

In the rGO-based methods for detection of tobramycin (TOB) in milk ([133,134]), both strategies provide low LOD values; however, both methods have limitations due to operational complexity. The AuNWs/PDA-rGO/AuE biosensor method used a CHA + Exo III dual enzymatic-DNA amplification cascade along with thionine-labeled probes to produce ultralow LOD values, yet requires precise handling of three different types of synthetic DNA probes, enzymes and nanomaterials. This complex procedure requires expensive materials, is time-consuming and prone to errors, and has only 9 days of stability and therefore cannot be used for practical or POC purposes [133]. On the other hand, the rGO/PIM/MIP/GCE method utilizes a two-phase pH-driven extraction process and an MIP-based detection process that offers good selectivity but at the expense of very long extraction times. Additionally, this method would require a multi-chamber set-up and manual handling of solutions, which hinders rapid or field-deployable analysis [134].

### 6.20. Vancomycin

Adane et al. developed an Au–Ag alloy nanocoral-cluster-decorated rGO sensor modified with poly(L-histidine) on a glassy carbon electrode for the simultaneous detection of CEF and vancomycin (VAN) in food samples, achieving exceptional sensitivity with pM-level LODs and a broad linear range (1 pM–290 µM) via irreversible oxidation signals in SWV [58]. The coral-like nanostructure provides a high electroactive surface area, while rGO ensures efficient electron transfer. Despite its analytical performance, the sensor’s practical deployment is hindered by a complex, multi-step fabrication process and the use of costly bimetallic nanomaterials.

Table 3 summarizes the analytical characteristics of the reduced graphene oxide-based sensor platform.

## 7. Meta-Analysis of Graphene-Based Electrochemical Sensors and Biosensors for Antibiotic Detection

This section synthesizes the performance metrics (LOD, LDR), reproducibility, and real-world applicability of the 94 studies reviewed in Section 4, Section 5 and Section 6.

### 7.1. Descriptive Meta-Analysis and Quality Assessment of Examined Studies

**Figure 5:**
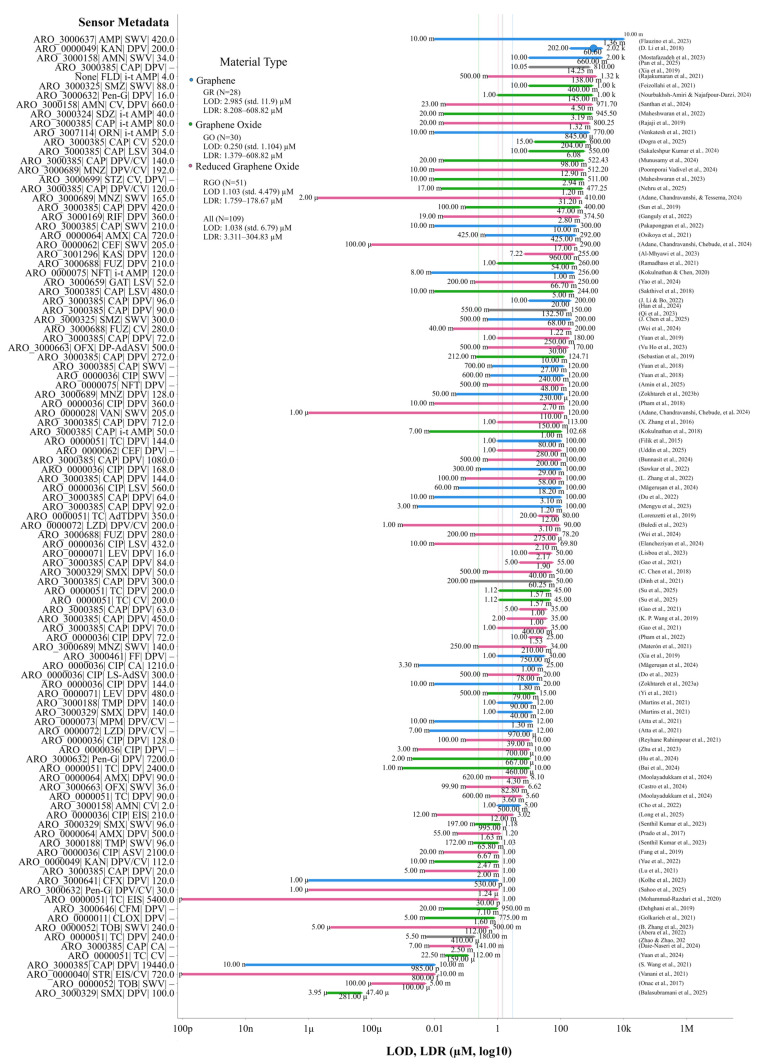
Descriptive meta-analysis forest plot comparing LOD and LDR performance across 94 studies. Key elements: (left y-axis) author names and publication years; (right y-axis) sensor and biosensor metadata including ARO antibiotic ID, electrochemical method, and accumulation time; (x-axis) LOD values in µM on log_10_ scale (10^−12^ = 1 pM, 10^6^ = 1 M); (vertical lines) the three colored lines represent the mean LOD for each material type (GR = blue, GO = green, RGO = red), and the dark grey line = the overall average [43,44,45,46,47,48,49,50,51,52,53,54,55,56,57,58,59,60,61,62,63,64,65,66,67,68,69,70,71,72,73,74,75,76,77,78,79,80,81,82,83,84,85,86,87,88,89,90,91,92,93,94,95,96,97,98,99,100,101,102,103,104,105,106,107,108,109,110,111,112,113,114,115,116,117,118,119,120,121,122,123,124,125,126,127,128,129,130,131,132,133,134,135,136].

**Figure 6:**
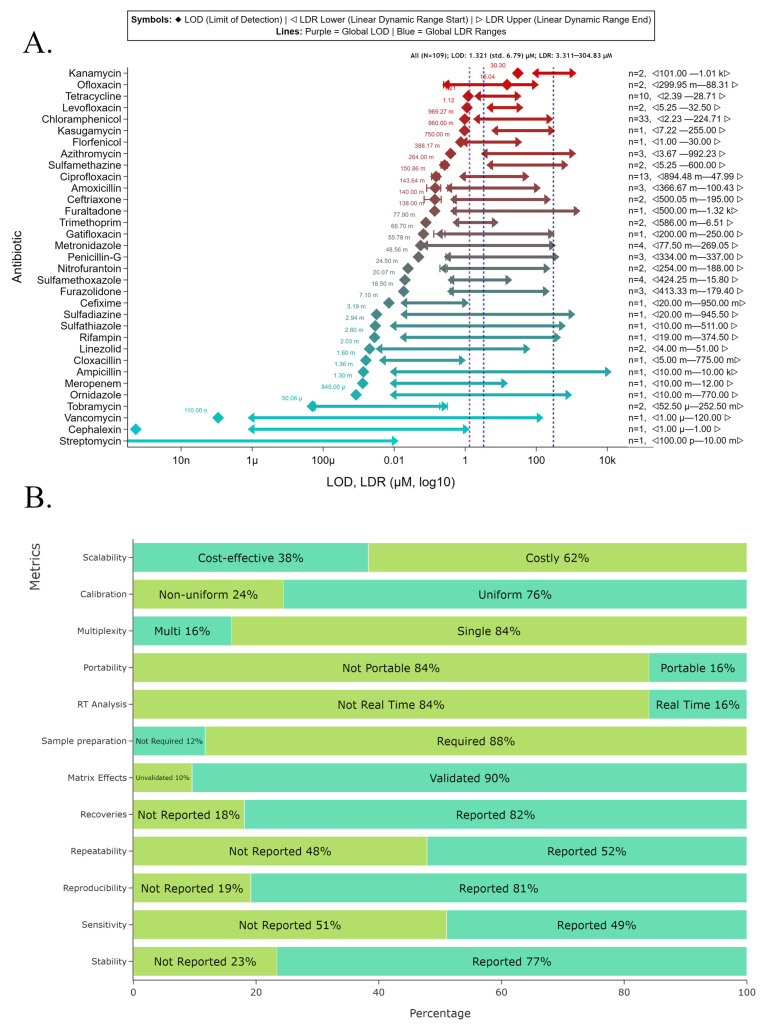
Antibiotic-grouped meta-analysis and quality reporting assessment. (**A**) Forest plot grouped by antibiotic class, showing: (blue lines) average LDR lower and upper bounds with 10% error margins; (purple line) average LOD across antibiotics; (right y-axis) antibiotic names arranged by analytical performance; (left y-axis) number of entries per grouping and ranges. (**B**) Quality reporting metrics, showing: (green bars) 74–82% reporting stability, reproducibility, and recoveries and only 16% reporting portability and real-time capability; (orange bars) ~50% reporting repeatability and sensitivity. Green bars represent positive trends while orange bars represent negative trends.

The metrics of LOD and LDR were compared across 94 studies (Figure 5). The forest plot for these metrics is presented in Figure 5 in µM units on a log_10_ scale. In Figure 5, 1 on the x-axis corresponds to 1 µM value, with values represented in engineering notation dipping below or above this scale to represent the orders of magnitude the LDR values span. Therefore, a notation of 10^−12^ is equivalent to 1 µ, and 10^6^ would correspond to 1 M on the reading scale. The y-axis presented on the right side (Figure 5) represents the sensor metadata with the ARO ID associated with the given antibiotic, 3–4 letter abbreviation, electrochemical method and calculated accumulation time from voltammograms associated with corresponding sensor entries. In the case of the absence of accumulation time, a “–” symbol is denoted. On the left-hand y-axis, the references are presented as author names and years of publication. The antibiotic FLD as of yet lacks a corresponding ARO ID, as is mentioned in Figure S2 in the Supplementary Materials. These IDs have been fuzzy-matched to their corresponding antibiotic names using thefuzz (0.22.1) library with a threshold of 85. Additionally, four vertical lines are presented in Figure 5: the three colored lines represent the mean LOD values corresponding to each material type, and the dark grey line represents the overall average. Additionally, when sensor metadata is shared by two entries, their references are combined on the right y-axis. In instances where combined entries have different material types, the color is rendered as grey; otherwise, they retain their corresponding colors, as described in the Figure 5 legend. In contrast, Figure 6A groups the sensors and biosensors’ performance using antibiotics and the grouped sensors are presented with LOD and LDR metrics with 10% error margins (for number of entries > 2) and performance hierarchy in Figure 6A. This plot presents a pair of blue lines representing the average LDR lower and upper bounds, and the purple line presents the average LOD across the antibiotics. The y-axis on the right side of Figure 6A displays antibiotic names, and the left side of the y-axis presents the number of entries in the grouping with averaged lower and upper LDRs. The analytical quality reporting metrics are presented in more than 74–82% reporting for stability, reproducibility and recoveries. However, repeatability and sensitivity measures are presented, on average, in only half of the studies (Figure 6B). It is indicated that the requirement for sample preparation, together with real-time analysis capability and portability, is demonstrated by less than 3% of the studies examined.

Sensors and biosensors were grouped in ascending order of analytical performance, and representatives were selected from the low, middle, and high-performing categories (Figure 5 and Figure 6). The low-performing group demonstrates significantly higher LOD values (average 12.5 µM) with limited detection ranges (average LDR 6.3–91 µM) and generally poor analytical performance. These include sensing materials such as GO/ZnO NRs, ERGO, AgNPs/MnO_2_/ErGO, MACGS and LEG [50,68,69,79,83]. The middle-performing group shows moderate analytical capabilities with an average LOD of 0.155 µM and substantially improved detection ranges (average LDR 5.244–365.62 µM). This group includes rGO@PDA@AuNPs, NiAl-LDH/rGO, Cu-Ag/GO, rGO/β-CD and GO/MWCNT [51,62,96,105,112]. The highest-performing sensors exhibit exceptional analytical capabilities with extremely low average LOD values (9.75 × 10^−9^ µM) and broad detection ranges (average LDR from 2.06 × 10^−5^ to 140.402 µM). This group includes state-of-the-art nanomaterials such as GQD/CFX-BSA antibodies, RGO/AuNPs/aptamer, TA-Au-Ag-ANpM/r-GO/poly(glycine), Au-Ag-ANCCs/r-GO/poly(L-histidine) and AuNPs/RGO/aptamer [46,58,63,127,132].

Additionally, the specialized group of portable sensors requiring no sample preparation demonstrates varied analytical performance but excellent utility for field application according to the ASSURED criteria. This group includes rGO, rGNR, and AA-RGO/CTAB sensing materials as representatives [49,54,94]. Moreover, sensors with a stability range of 30–90 days were observed for MACGS, Ti_3_C_2_T_x_@Au-AS-GQD aerogel, Graphene, GO/MnFe_2_O_4_, Cl-RGO and rGO/Au/Co_2_CuS_4_ [45,79,84,118]. Finally, the studies from authors who reported electrode sensitivity values are considered. The highest sensitivity electrode materials demonstrate exceptional response to target analytes, with a value of 96.92 nA·μM^−1^·cm^−2^. The prominent sensing materials include GR/Fe_3_O_4_NPs, FeMn_2_O_4_-rGO, Zn_2_SnO_4_/rGO/NF, N-prGO, rGO/MoS_2_ [57,89,115,121]. These findings are presented in Table 4.

### 7.2. Characteristic Analysis of the Graphene-Based Sensors and Biosensors for Antibiotic Detection

**Figure 7:**
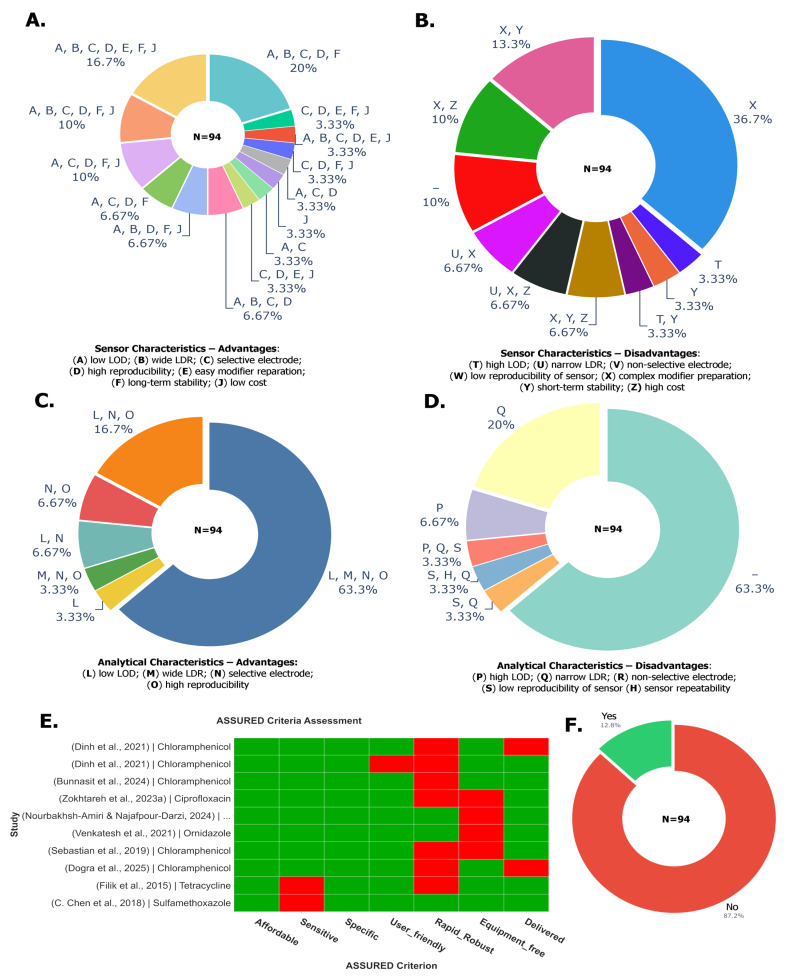
Comprehensive assessment of sensor and biosensor characteristics and POC-readiness across 94 studies. (**A**) Advantages; (**B**) Disadvantages; (**C**) Analytical Advantages; (**D**) Analytical Disadvantages; (**E**) heatmap of ASSURED criteria dimensions [45,54,86,92,95,96,98,101,119]; (**F**) POC-readiness status visualization showing 87.2% not ready vs 12.8% ready.

The coded advantages and disadvantages of the detection method and sensors and biosensors’ characteristics were not split into individual datapoints in order to explore the co-occurrence of shared characteristics. These results are summarized in Figure 7A–D. Here, when a value combination occurs with less than 2% frequency, it is combined into an “Other” category label. Together, sensor characteristic advantages (Figure 7A) related to stability, reproducibility, low LOD and wide LDR performance, easy fabrication, and selectivity of electrode appear positively in 41.3% of the studies. Similarly, for disadvantages (Figure 7B), complex modifier preparation individually (30.3%) and in combination with high cost (14.7%) or short-term stability (16.5%) are outstanding challenges in the field. Regarding the method characteristics (Figure 7C), 69.7% of sensors and biosensors demonstrate potential in being a performing, reproducible and selective alternative for electrochemical determination of corresponding target analytes. However, improvements are needed in terms of narrow LDR performance (16.5%). DPV was the most common electrochemical method (52.3%), followed by its use in conjunction with CV (an additional 7.3%). Finally, costly modifier preparation (35%), non-uniform calibration at lower concentrations (24%) and explicitly indicated matrix effects (11%) are challenges that should be targeted. Additionally, multiplexicity (only 20%) could be essential in terms of utility for environmental monitoring applications.

### 7.3. POC-Readiness Assessment

The POC-readiness assessment was evaluated through code-mapping for positive and negative scores on ASSURED criteria (Table S2 in Supplementary Materials). POC-readiness analyses for studies scoring high on ASSURED criteria are presented in Figure 7E,F. Figure 7E shows the breakdown: only 12 studies met ≥4 ASSURED criteria (Affordable, Sensitive, Specific, User-friendly, Rapid, Equipment-free, Deliverable). Key barriers visible in Figure 7B,D: Complex modifier preparation (30.3%) and high cost (14.7%) are the dominant disadvantages preventing POC deployment. Figure 7F shows a pie chart that visually emphasizes the 12.8% vs 87.2% split; this quantifies the “valley of death” between academic innovation and field-deployable devices discussed in Section 8.5.

### 7.4. Meta-Analysis Across Antibiotic and Sensor Type Groupings

The meta-analysis was performed according to the Cochrane guidelines, specifically modified for sensor and biosensor performance metrics analysis. According to the general criteria of a meta-analysis, at least three studies per group are required for heterogeneity estimation using the percent-total variation statistic (I^2^) and estimating meaningful confidence intervals (CI). In this analysis, the lower and upper LDR were converted to absolute ranges to calculate the dynamic range width in μM units. LOD and response time metrics were analyzed in raw units (μM and seconds, respectively). Performing the initial quality filtering steps (sensitivity, stability, reproducibility, repeatability and recoveries) reduced the number of datapoints to 86 out of 109 from the 94 examined studies. Subsequently, after grouping by antibiotic class and sensor material type, 37 studies remained, which consisted of eight groupings with at least three studies within each group (antibiotic class × nanomaterial type). Meanwhile, for the response time metric, only 33 studies with seven groupings fulfilled the requirements for meta-analysis. The analysis consisted of three unique antibiotic groups across five unique nanomaterial categories. These studies had an average quality score of 4.03 out of 5 after filtering and an average I^2^ of 76.2%. Moreover, nine groups were compared using Bayesian adjustment due to group size ≤ 4. A random-effects model with Hartung–Knapp–Sidik–Jonkman (HKSJ) adjustment was employed owing to I^2^ ≥ 25%. Additionally, Cochran’s Q test was performed to estimate the chance expectation of observed differences. The metrics LOD and response times were log-transformed to prevent negative CI bounds, while pooled means were reported as geometric means during reporting. The CIs were computed using a t-distribution (HKSJ adjustment) to ensure appropriate coverage probability. The prediction intervals (PI) were calculated to describe the expected range of true effects in future studies, which is essential given the high heterogeneity observed. The fixed-effects model analysis was conducted for sensitivity reporting purposes. Prediction intervals were observed to be consistently wider than confidence intervals—1.31× for LDR width, 13.53× for response time and 460× for LOD—further emphasizing the true between-study uncertainty. For groups containing fewer than four groupings, Bayesian credibility intervals were computed. Finally, a GRADE certainty assessment based on risk of bias, inconsistency, imprecision, indirectness and publication bias was performed on each outcome to evaluate confidence in the findings.

**Figure 8:**
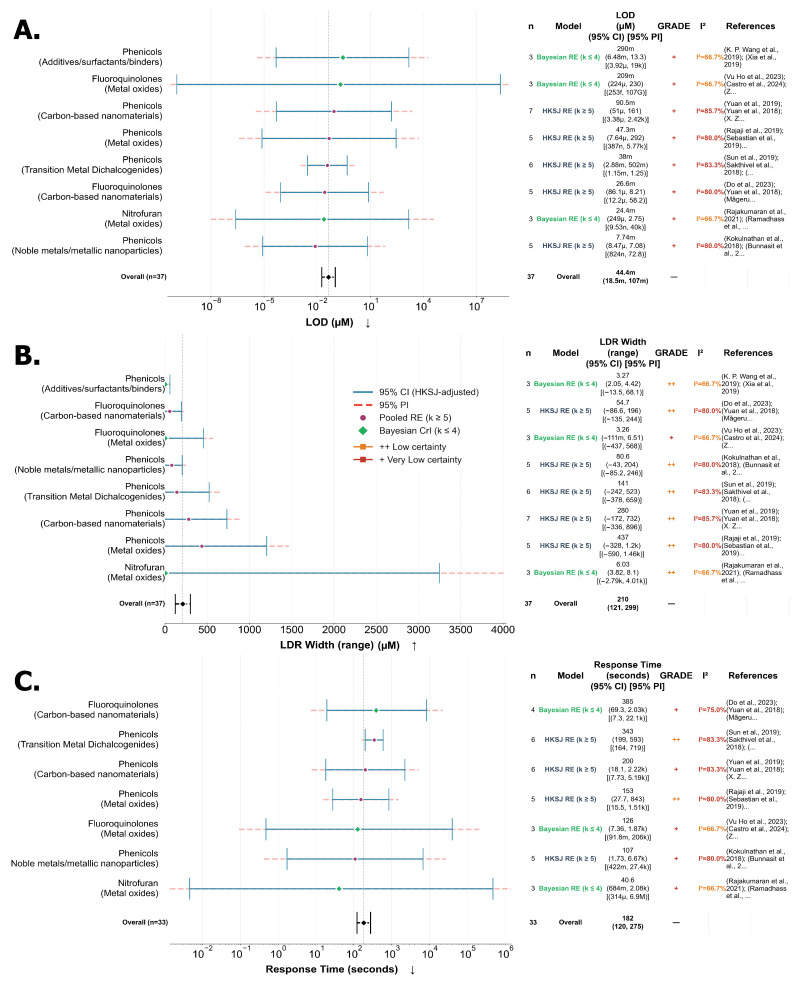
Random-effects meta-analysis with HKSJ adjustment for three performance metrics (n = 37 studies, eight groupings). (**A**) LOD performance: best group = phenicols/noble metals (RE-HKSJ: 0.007 µM, CI: [0–7.082] µM, PI: [0–72.761] µM, I^2^ = 76.1%); second best = fluoroquinolones/carbon-based (0.026 µM, CI: [0.0001–8.297] µM). (**B**) LDR width performance: best group = nitrofurans/metal oxides (611.16 µM range, Bayesian CI: 3.82–8.10 µM). (**C**) Response time performance: best group = nitrofurans/metal oxides (46.54s, CI: [0.0046 s–30 h], Bayesian 95% CrI: 0.6842 s–34.7 min) All grouping studies are extracted from these references [47,52,55,59,60,62,65,67,69,75,77,82,83,84,86,87,90,96,97,98,99,100,101,103,116,117,118,119,120,121,129]; some studies contribute more than one based on differing sensors, antibiotic groups, or electrochemical techniques, these are split accordingly: [101] (n = 4), [52] (n = 2); [82] (n = 2), [87] (n = 2).

The LOD metrics had a pooled mean range (RE-HKSJ: 0.007–0.272 μM, n = 8 groups) with considerable heterogeneity (mean I^2^ = 76.1%). In this analysis, the lower values indicate better performance. The results indicate that phenicols determined using noble metals/metallic nanoparticles were the best-performing group (RE-HKSJ: 0.007 μM, CI: [0–7.082] μM, PI: [0–72.761] μM). Additionally, fluoroquinolones determined using carbon-based nanomaterials (n = 5, RE-HKSJ: 0.026 μM, CI: [0.0001–8.297] μM, PI: [0–58.176] μM) were the second-best performing group (Figure 8A). Notably, three groups used Bayesian random-effects models with credible intervals for stabilized estimation and yielded narrower and more robust estimates in comparison with frequentist methods. Nitrofurans determined using metal oxides were the best-performing group with a 611.16 μM range (n = 3, CI: [−2024.52–3246.85 μM range], PI: [−2791.49–4013.82 μM range]). The Bayesian credible interval for this group was between 3.82 and 8.10 μM (Figure 8B). Finally, the response times (reported in seconds) were log-transformed across eight groups (RE-HKSJ: 46.54–401.63 s) and also demonstrated considerable heterogeneity (mean I^2^ = 76.4%) across seven groupings. Here, lower values indicate better performance. Nitrofurans determined using metal oxides were the best-performing group in this analysis (n = 3, RE-HKSJ: 46.54 s, CI: [0.0046 s–30 h], PI: [0.003 s–80 d]), while Bayesian 95% credibility intervals at 0.6842 s–34.7 min respectively indicated a more realistic approximation of the response time (Figure 8C).

### 7.5. Limitations of Meta-Analysis

The Egger’s test requires ≥ 10 studies per group; therefore, this analysis relied on proactive screening during data collection to minimize the publication bias. The meta-analysis of response time was particularly challenging due to variable methodologies and missing values and may introduce selection bias in this particular analysis. Moreover, while extensive care was taken to normalize LOD and LDR values in μM, minor rounding errors are possible. Overall, these results highlight the applicability of various sensor groups in estimating antibiotics in varied matrices. The choice of nanomaterial and fabrication parameters varies significantly among the formulated groupings, and thus, wider CIs and even wider PIs are observed. For example, in fluroquinolones/metal oxides, such intervals are indicative of extensive heterogeneity. Therefore, future research should target nanomaterials for high-priority antibiotics and emphasize quality reporting variables for informative comparisons. Additionally, for well-established nanomaterials, standardization of fabrication and reporting protocols is critical.

Notably, phenicols/noble metals showed the lowest point estimate; however, the wide prediction intervals suggest that a new incorporation of studies could yield significantly higher LODs. While this result is promising, the consistency across laboratories and fabrication consistency remains challenging in this grouping. The negative lower bounds in LDR width CIs do not rule out zero or negative dynamic ranges; however, Bayesian estimates provide a more realistic estimate of the likely performance. In general, larger sample sizes with finer divisions could identify the true effects. However, it is not applied, as further divisions may altogether filter out many relevant sub-groups from a quantitative meta-analysis. It is noteworthy that phenicols are also the most represented group.

There is no single optimal nanomaterial; rather, the best choice depends on the target antibiotic and application priorities, as highlighted in previous sections. Additionally, biosensors, while represented in our dataset, did not satisfy the grouping criteria at all, even though, LOD-wise, they were some of the best-performing sensors, as indicated by the descriptive meta-analysis. Additional details regarding the individual groups contributing to the groupings are described in Figure S3 in the Supplementary Materials. Furthermore, carbon-based and noble metal-functionalized sensors and transition metal dichalcogenides are adequately explored with considerable heterogeneity. The pooled estimates may represent the average of diverse effects rather than a single true effect.

## 8. Comparative Analysis of Electrochemical Detection of Antibiotics by Graphene-Based Sensors and Biosensors

A systematic comparative evaluation of the 94 reviewed studies reveals critical insights into the performance, design strategies, and practical applicability of graphene (GR), graphene oxide (GO), and reduced graphene oxide (RGO)-based electrochemical biosensors for antibiotic detection. This analysis is structured around six key dimensions: (i) transducer architecture and recognition elements, (ii) analytical performance, (iii) matrix compatibility and real-sample validation, (iv) practical deployability, (v) POC readiness assessment and (vi) cost assessment and economic viability.

### 8.1. Transducer Architecture and Recognition Strategies

**Figure 9:**
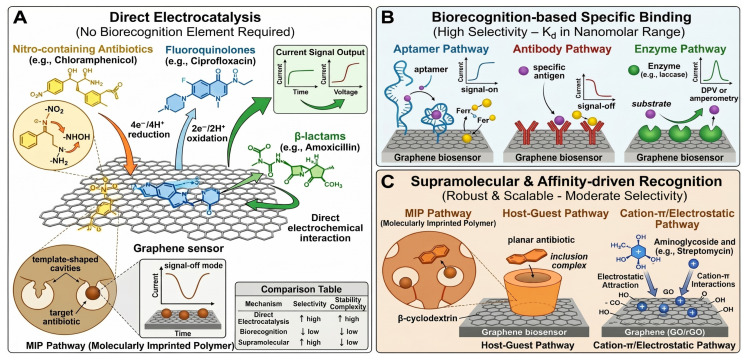
Schematic illustration of the molecular recognition paradigms governing antibiotic detection on graphene-based electrochemical platforms. (**A**) Direct Electrocatalysis: Label-free detection strategies exploiting the intrinsic electroactivity of antibiotics. (**B**) Biorecognition-Based Specific Binding: Mechanisms utilizing biological recognition elements for high selectivity. (**C**) Supramolecular and Affinity-Driven Recognition: Robust and scalable approaches based on non-covalent interactions. An embedded Comparison Table in Panel A summarizes the trade-offs in selectivity, stability, and complexity across these mechanisms.

To critically evaluate the translational potential of graphene-based platforms, it is essential to first categorize the underlying molecular recognition strategies, as these dictate not only analytical performance but also fabrication complexity and economic viability. Figure 9 provides a schematic classification of the three dominant paradigms identified in this review: (A) Direct Electrocatalysis, which leverages the intrinsic redox activity of antibiotics for label-free detection; (B) Biorecognition-Based Specific Binding, utilizing high-affinity biological elements (aptamers, antibodies, enzymes) for superior selectivity; and (C) Supramolecular and Affinity-Driven Recognition, employing robust synthetic hosts (MIPs, cyclodextrins) and electrostatic interactions for scalable deployment.

#### 8.1.1. Direct Electrocatalytic Recognition

Nitro-containing antibiotics, for example, chloramphenicol, metronidazole, nitrofurantoin, and furazolidone, can be reduced at relatively mild cathodic potentials through a well-defined 4e^−^/H^+^ reduction of their NO_2_ groups to NHOH (hydroxylamine). This is amplified significantly in rGO- or GO-based nanocomposites because they provide higher rates of electron transfer as well as greater adsorption of the reaction products. For example, an rGO–Fe_3_O_4_ nanohybrid allows for the low potential detection of CAPs through the stabilization of intermediate species during the reduction process [116]. Fluoroquinolones, such as ciprofloxacin and levofloxacin, are susceptible to irreversible 2e^−^/2H^+^ oxidation of their piperazinyl rings; however, this can be greatly improved using GR or rGO substrates that have been functionalized with metals, metal oxides, or polymers. SDS-modified graphene electrodes, for example, electrostatically enrich CIP molecules near the surface, amplifying the oxidation signal [85]. β-lactams like amoxicillin contain phenolic groups that can be oxidized via enzyme-mediated (laccase) or metal-catalyzed pathways on GR/AuNP platforms [53]. Advantages include simplicity, cost-effectiveness, and rapid response, but drawbacks involve limited selectivity in complex matrices and susceptibility to redox interferents (ascorbic acid, uric acid). Hence, this approach is most effective for purified or semi-purified samples (pharmaceutical formulations) or when combined with pre-separation steps.

#### 8.1.2. Biorecognition-Based Specific Binding

To achieve high selectivity, especially in real-world matrices, many studies integrate biological recognition elements onto graphene platforms:

(i) Aptamers: Single-stranded DNA or RNA oligonucleotides selected via SELEX for high-affinity binding to specific antibiotics. Aptasensors are widely used for ciprofloxacin [64,136], kanamycin [43], penicillin-G [114], tetracycline [132] and streptomycin [63]. Binding often induces conformational changes (folding into G-quadruplex structures) that modulate the electron transfer of redox probes like Fe(CN)_6_^3−/4−^, measurable via EIS or DPV. Signal amplification strategies such as Exo-III-assisted cyclic amplification [81] or catalytic hairpin assembly [133] push LODs into the femtomolar range but increase assay complexity.

(ii) Antibodies: Antigen–antibody binding is used in immunosensors for beta-lactam compounds (cephalexin [46]) and for tetracycline compounds, which have been shown to inhibit redox probe diffusion or create a change in interfacial impedance. Although these are very selective for their target, they can be unstable at higher temperatures, expensive to produce, and have variation in each batch of antibody produced, which limits their use in the field.

(iii) Enzymes: Horseradish peroxidase (HRP) or laccase have been used as catalysts to convert antibiotics into oxidized forms (amoxicillin [53]). Enzyme-based biosensors are very good at detecting specific antibiotics, but they can lose their effectiveness if the temperature or pH changes, so it is important to keep those conditions stable.

Biosensors based on biorecognition offer both high selectivity and affinity (K_d_ values typically in the order of nanomolar) toward the analyte of interest; however, they are generally less durable and less suitable for prolonged exposure to environmental conditions or on-site use due to the instability and fragility of the biological molecules and the requirement for cold chain storage.

#### 8.1.3. Supramolecular and Affinity-Driven Recognition

This new generation of recognition can identify substances without biomolecules and relies on engineered chemical affinities:

(i) Molecularly Imprinted Polymers (MIPs): Cavities that are formed through a synthetic process called electropolymerization of a target antibiotic (tetracycline [106,107] or sulfamethazine [93]) create cavities around a template antibiotic. After the removal of the original template, these cavities will selectively bind the original target, and they will do so in a “signal-off” mode by interfering with access to the probe. Although MIPs provide good stability and reusability, they have drawbacks such as template leakage, heterogeneous binding sites, and slow rebinding kinetics.

(ii) Host–Guest Chemistry: Functionalizing β-cyclodextrin (β-CD) onto rGO/GO produces hydrophobic inclusion complexes with planar or bulky antibiotics (azithromycin [66], ciprofloxacin [122], nitrofurantoin [51]). This method of pre-concentrating analytes increases sensitivity while improving moderately selective binding due to steric compatibility.

(iii) Cation-π and electrostatic attractions: Aminoglycosides (kanamycin and tobramycin) are polycationic at physiological pH and can form strong electrostatic bonds to negatively charged GO [43,134]. Tetracyclines can be captured through cation-π interactions using either Cu^2+^-doped membranes or montmorillonite clays [108,111].

The advantages of these methods include their robustness, low costs, and ability to be scaled up; however, they generally possess less selectivity than biorecognition-based detection systems, especially when detecting compounds that belong to the same antibiotic family but have different structures. Future developments will most likely involve the use of hybrid recognition schemes such as MIP–aptamer conjugate molecules or enzyme–MOF composites to synergistically improve both analytical performance and environmental durability.

### 8.2. Analytical Performance Benchmarking

The performance characteristics were evaluated among different antibiotic classes and graphene derivative materials (Table 1, Table 2 and Table 3). The lower operating detection (LOD) values ranged from femtomic amounts (0.985 fM for CAP using an Exo-III amplified aptasensor [81]) to micromoles (60.6 µM for kanamycin using a ssDNA sensor [88]). The dynamic range of the sensors varied greatly: many of the MIP/aptamer sensors had very limited LDRs (≤2 orders of magnitude), whereas direct electrocatalytic sensors (especially those sensing nitro-containing antibiotics) demonstrated much greater LDRs (≤3–4 orders). A notable feature was that sensors that targeted antibiotics with structures that are similar (fluoroquinolone and tetracycline antibiotics) displayed cross-reactivity unless they included a highly selective layer of biorecognition.

### 8.3. Real-Sample Applicability and Matrix Effects

More than seventy percent of studies have verified that performance occurs on real-world matrices (milk, urine, waterways/wastewater, meat). The food matrices (milk, egg and meat) have been particularly difficult due to the presence of fats/proteins, which can foul sensors; defatting/filtration has therefore often been required [46,134]. Environmental waters (tap/river) are less difficult to measure; however, a pH adjustment and/or interference testing is normally required. Recovery values (85–110%) were typically good; however, those with very low LODs had poor reproducibility in the presence of multiple components in the matrix due to non-specific adsorption.

Repeatability (intra-assay precision on the same electrode) is typically reported as RSD ≤ 5% for DPV/SWV methods but degrades in complex matrices due to surface passivation. Selectivity remains challenging for structurally analogous antibiotics (fluoroquinolones, tetracyclines). Strategies to enhance selectivity include: (i) molecular imprinting, creating shape-complementary cavities; (ii) aptamer SELEX targeting unique functional groups; (iii) potential window optimization to isolate specific redox peaks; and (iv) Differential pulse techniques suppressing capacitive background. Cross-reactivity studies using interferents (ascorbic acid, uric acid, glucose, structurally similar drugs) must be mandatory in future reporting. Selectivity coefficients and interference tolerance thresholds should be standardized to enable fair benchmarking. The real-sample validation performance of graphene-based sensors and biosensors is mentioned in Table 5.

### 8.4. Practical Deployability and Scalability

Deployability was assessed using three criteria: portability (screen-printed or flexible electrodes), fabrication complexity, and stability. GO/rGO-based SPCEs ([95,102,124]) and laser-induced graphene platforms [83,131] scored highest in portability but often required multi-step modification. Stability was a critical limitation: while 30–90-day storage stability was reported for some GR composites [79,80], most aptasensors or enzyme-based systems degraded within 1–2 weeks. Scalability was hindered by (i) the use of costly reagents (Au/Ag NPs, rare earth dopants), (ii) multi-day synthesis (MOF/MIP templating), and (iii) a lack of regeneration protocols. Only a handful of sensors achieved true point-of-care readiness, combining low-cost substrates, minimal sample prep, and smartphone integration [54,96]. In summary, while graphene derivatives enable ultrasensitive antibiotic detection, the optimal platform depends on the application context: rGO-metal hybrids excel in controlled lab settings requiring low LODs, whereas GO-functionalized SPCEs offer better trade-offs for field deployment despite moderate sensitivity. Future efforts should prioritize simplifying fabrication, enhancing antifouling properties, and validating performance under real-world variability.

### 8.5. POC Readiness Assessment of Graphene-Based Sensors and Biosensors

In order to evaluate the potential of graphene-based electrochemical biosensors to have the ability to be used at the POC (and in turn deployed into the “real world”), we evaluated each of the 94 studies that were examined against a standard set of criteria, which are based on the WHO’s ASSURED framework, as well as on recent biosensor validation guidelines [84]. The criteria are (i) ease of use, (ii) portability, (iii) minimal or no need for sample pretreatment, (iv) fast detection time (<10 min), (v) stability under ambient (and/or field) conditions, (vi) low cost, and (vii) deliverability.

Among the 94 studies, only 12 (12.8%) fulfilled four or more of these criteria, qualifying as POC-ready prototypes. Most point-of-care compatible designs also include a paper-based r-GNR sensor for the simultaneous detection of sulfamethoxazole/trimethoprim in tap water [94]. An AA-RGO/CTAB-modified solid phase extraction (SPE) for sulfamethoxazole in lake/urine samples with surfactant-enhanced pre-concentration [54] and a C60/rGO/Nafion SPE for metronidazole in synthetic urine with analysis times under 3 min [128] were developed. Despite these advances, critical gaps persist:

- Sample Pretreatment: Approximately 85% of POC sensors still require defatting, filtration, pH adjustment, or dilution steps that are incompatible with lay user deployment.
- Stability: Only about five studies report stability at ambient temperature for more than thirty days; most were stored in refrigerators or used for short time frames (less than fourteen days).
- Multiplexing vs. Specificity: Although some sensors targeted multiple antibiotics (penicillins [110] and tetracyclines [111]), they lacked compound-specific resolution, limiting their utility for clinical use.
- Scalable Fabrication: Due to the complexity of nanomaterial synthesis (MOFs, core-shell NPs, MIP templating), large-scale, low-cost manufacturing is difficult.

None of the sensors examined fully met all seven POC criteria. The closest options used a mix of r-GO/GO with SPEs and did not rely on enzymes or aptamers for recognition. The development of future POC devices should focus on integrated sample-to-answer microfluidics, antifouling surface engineering and open-source electronics for universal smartphone connectivity. So, even though graphene derivatives are promising materials for monitoring antibiotics at the POC, most research is still just in the early testing stages in labs. Closing the “valley of death” between academic innovation and field-deployable devices will require co-design with end users (for example, farmers, clinicians, and environmental inspectors) and adherence to standardized POC validation protocols.

### 8.6. Cost Assessment and Economic Viability of Graphene-Based Sensors and Biosensors

The economic viability of graphene-based electrochemical biosensors is an important factor that determines their practical utility in resource-constrained environments such as farms, low- and middle-income country clinic sites and decentralized environmental monitoring systems. In order to determine the cost effectiveness of graphene-based electrochemical biosensors, we assessed each of the 94 examined studies against three important economic criteria: (i) material cost (noble metals, rare earth elements or commercially available biorecognition elements), (ii) fabrication process (complex multi-step synthesis, clean room processing or specialized equipment), and (iii) scalability for large-scale manufacturing (compatible with roll-to-roll printing, screen printing or disposable formats).

#### 8.6.1. Material Cost Profile

The examined sensors and biosensors (58/94, or 61.7%) were based on high-cost materials, which included noble metal nanoparticles (Au, Pt, Pd), used in 39 studies ([58,100,110,123]) for enhancing conductivity or immobilizing aptamers.

- Gold alone can account for >60% of electrode material costs in lab-scale sensors.
- Rare earth or transition-metal oxides (Eu_2_O_3_, GdVO_4_, SmMoSe_2_, HoVO_4_): Featured in 11 sensors ([74,75,76]) for catalytic enhancement—materials that are not only expensive but also geopolitically constrained.
- Biological recognition elements: 10 studies employed aptamers or antibodies ([43,63,64,65,78,110,111,121,132,133]), which require costly synthesis, purification, cold-chain storage, and stability testing, adding $50–$500 per batch depending on sequence length and modifications.

Conversely, 36 studies (38.3%) avoided high-cost additives, relying instead on (i) pure or doped carbon nanomaterials (RGO, GO, graphene) combined with earth-abundant catalysts (Fe_3_O_4_, MnO_2_, ZnO, CuO); (ii) polymer-based modifiers (chitosan, polyaniline, Nafion); and (iii) label-free Direct Electrocatalysis strategies that eliminate biological reagents. These low-cost designs generally reported sensor fabrication costs below $5 per unit, compared to $15–$100/unit for noble metal- or aptamer-based platforms.

#### 8.6.2. Fabrication Complexity and Scalability

Only 18 studies (19.1%) utilized fabrication workflows compatible with scalable manufacturing:

- Screen-printed electrodes (SPEs) modified with drop-cast nanocomposites ([67,101,119]). These can be mass-produced at >10,000 units/day using industrial screen printers.
- Laser-induced graphene (LIG) on polyimide ([83,131]): Enables mask-free, single-step patterning without cleanroom facilities.
- Paper- or polymer-based disposable platforms ([94]) are ideal for single-use POC applications.

However, 76 studies (80.9%) involved complex, multi-step protocols, such as: (i) hydrothermal/solvothermal synthesis (>12 h, high T/P); (ii) electropolymerization of MIPs with precise potential control; (iii) click chemistry or covalent bioconjugation requiring inert atmospheres; and (iv) template removal steps (for MIPs or MOFs) that risk structural collapse. These processes are labor-intensive, require trained personnel, and are difficult to automate, posing significant barriers to commercial translation.

#### 8.6.3. Economic Viability for POC Deployment

When cross-referenced with POC readiness criteria (Section 8.5), only 9 of the 12 POC-ready sensors also qualified as low-cost (i.e., <$10/unit, no noble metals, no biorecognition elements). Notable examples include: AA-RGO/CTAB/SPE for sulfamethoxazole [54], which uses ascorbic acid-reduced GO and a common surfactant for an estimated cost of ~$2/unit; Sn/rGO/SPE for chloramphenicol [119], as tin is abundant and non-toxic, with fabrication via simple sonication and drop-casting; and rGNR/parchment paper for SMX/TMP [94]. Highly sensitive and fast sensors (aptasensors using Exo-III with fM LODs [81,133]) are too expensive for daily practice because they rely on enzyme (and other) substrates, DNA typically produced on an individual basis, and gold nanoparticles. Therefore, these sensors cannot be implemented in field settings. The cost–benefit comparison between traditional and graphene-based sensors and biosensors is summarized in Table 6.

Graphene-based derivatives can provide a versatile and very sensitive transducer substrate; however, most of the biosensors studied here cannot be economically deployed at a large scale for POC use. Future efforts should prioritize reagent-free designs, earth-abundant catalysts, and additive manufacturing-compatible processes to bridge the gap between laboratory innovation and field-deployable affordability. Cost must be treated not as a secondary metric but as a primary design constraint, especially in applications targeting antimicrobial stewardship in low-resource environments.

### 8.7. Reproducibility and Long-Term Stability: Current Gaps and Standardization

There are several unexplored issues with regard to reproducibility, as well as long-term operational durability/stability; these issues have been addressed in fewer than 20% of studies. Studies provide RSD values for reproducibility 77% of the time and stability 81% of the time, with most of those being measured under refrigerated conditions. A common set of degradation mechanisms associated with graphene oxide nanoparticles in aqueous solutions includes (i) nanoparticle release from the composite matrix; (ii) oxidation of the basal plane of graphene oxide; (iii) protein/fat adsorption on to the surface of the device, which damages the biorecognition element; and (iv) denaturation of biorecognition elements (aptamers/enzymes) due to exposure to environmental conditions. In order to address this issue and make devices more commercially viable, we suggest a standardization of data collection and reporting practices, including (a) measuring the stability of the device over at least thirty days at room temperature (25 °C); (b) measuring the reproducibility of an array of devices made using similar methods, where RSD < 8%; and (c) establishing explicit storage requirements. The use of inexpensive “earth-abundant” materials such as Fe_3_O_4_, ZnO, and MnO_2_ for modifying surfaces combined with screen-printed architectures has resulted in improved batch-to-batch reproducibility compared to the use of noble metal- or MOF-based platforms.

### 8.8. Pathways to Commercialization and Regulatory Readiness

Commercialization is tied to compliance with either ISO 13485 (medical device) [137] or a framework that aligns with EPA Method validations. The three most common obstacles are as follows: (1) there is no commonly used reference material for all types of complex matrices (raw milk, wastewater); (2) there are very strict regulatory requirements for any biologic component in your sensor (aptamer or antibody); and (3) the cost of integrating microfluidics into your product includes both non-recurring and recurring costs, which make it difficult to achieve commercial success. Using SPCE, LIG, or a substrate compatible with RTRM (roll-to-roll manufacturing) can improve commercial feasibility. To fabricate a sensor that meets end users’ workflow needs and also satisfies the need for PMS (premarket submission) by regulators, partnerships among universities, medical/diagnostic companies and end users in agriculture and clinical environments will be necessary.

## 9. Human Health Implications of Antibiotic Contamination

Antibiotics used in the clinic, on farms, and as pollutants in the environment pose numerous health risks to humans, well beyond their potential to treat bacterial infections. Antibiotics are necessary to treat infections; however, their use without regulation has led to an environmental health problem with a broad array of health-related challenges, including direct toxicity, allergic reactions, disruptions to the human microbiome, and the crisis of antibiotic-resistant bacteria.

### 9.1. Direct Toxicity and Adverse Physiological Effects

Certain antibiotic characteristics provide a toxicological profile that presents a risk of adverse effects to organisms exposed to them at levels below the toxic level. For example, chloramphenicol, which has been associated with idiosyncratic aplastic anemia and bone marrow suppression in humans, is banned from use on food-producing animals in several countries [138,139] and requires sensors with femtomolar sensitivity to detect it at low enough levels to ensure safe consumption [55,101]. In addition to their toxicological potential, certain antibiotics such as fluoroquinolones (ciprofloxacin, levofloxacin) are also linked to juvenile cartilage damage and adult tendonitis; tetracyclines have been shown to produce photosensitivity and teeth discoloration in some individuals [140,141]. Therefore, the sensors reviewed herein, especially those designed to target these high-risk classes of antibiotics within food matrices (Table 1, Table 2 and Table 3), provide critical barriers to prevent toxicological exposure to organisms in the food chain. The presence of antibiotics in environmental water samples illustrates how resistance genes can recirculate into human populations and thus render standard treatments ineffective for many common infections [142,143].

### 9.2. Exposure Pathways and Vulnerable Populations

The primary routes of human exposure include food, water and environmental contamination by antibiotics.

- Food: Animals given antibiotics to enhance growth or to prevent disease will eliminate undigested antibiotics into their meat, milk, and eggs. The high number of studies validating the use of sensors for detecting antibiotics in milk and meat suggests that this is a common source of human exposure [63,78,101].
- Drinking Water: Monitoring antibiotics in wastewater and river water using sensors [52,59] is an important method of assessing human exposure at the community level.
- Clinical: Monitoring antibiotic levels in patient serum and urine in clinical settings is an important method of therapeutic drug monitoring (TDM). TDM is necessary to avoid either underdosing and promoting antibiotic resistance or overdosing and causing toxic effects from the antibiotic [44,91].

Infants, the elderly and individuals who are immunocompromised are most susceptible to the harmful effects of antibiotics. Aminoglycoside antibiotics (kanamycin and streptomycin) have been shown to be particularly toxic and produce ototoxicity and nephrotoxicity in patients with decreased renal function [144]. Thus, the development of POC sensors for detecting antibiotics represents not just a technological advancement but also a public health equity imperative for rapidly providing screening capabilities in low-resource environments that lack the laboratory infrastructure to provide such services.

### 9.3. Microbiome Disruption and Long-Term Consequences

The presence of chronic low levels of antibiotic residue in the environment has been shown to have an impact on the human gut microbiome and cause a disruption in the balance of bacteria within it, as well as contributing to various types of disease including metabolic disease and weakening the body’s ability to fight off opportunistic pathogens such as *Clostridioides difficile* [144,145], which can be life threatening. Establishing safe threshold levels of antibiotic residue is necessary for protecting the integrity of the human gut microbiome from harm, versus simply preventing acute antibiotic toxicity.

### 9.4. The Critical Role of Monitoring in Health Protection

The results obtained from the synthesis of the data used in this review show an improvement in analytical performance (linear range, LOD); however, there are significant gaps in field deployability that continue to impede translation to protective public health outcomes. Proactive and timely health protection through on-site and immediate monitoring is required instead of relying on retrospective laboratory-based analysis. Through the development of graphene-based electrochemical sensors capable of detecting antibiotics in various matrices (farm milk to clinical urine), these sensors can enable the enforcement of regulations, promote stewardship of antimicrobials, and ultimately mitigate the adverse impact on human health due to the presence of antibiotics in the environment and/or the development of antimicrobial-resistant bacteria. The public health imperative for antibiotic monitoring directly aligns with global sustainability frameworks, as explored in the following section on SDGs.

## 10. Antibiotics Monitoring and UN Sustainable Development Goals: A Nexus Approach

In 2015, all member states of the United Nations (UN) adopted 17 goals to be achieved by 2030. These 17 goals are called Sustainable Development Goals (SDGs) for global development, to end poverty, protect nature, ensure peace and prosperity, eliminate global hunger, promote health and well-being for all, provide quality education and promote partnerships between member states and organizations. These goals are an urgent call for action by all countries in a global partnership to address challenges like inequality, poverty, climate change, environmental degradation, peace and justice. Sometimes, even if these challenges are country-specific, their adverse effects have a chain reaction, damaging global sustainable development. These goals were developed by countries over decades of work, and these goals are interlinked as well. The United Nations recognizes that poverty is interlinked with health and education, so ending poverty must go concurrently with strategies to improve health and education. There are multiple factors responsible for each deprivation, and some factors have both positive and negative effects, such as antibiotics. Antibiotics play a complex role in achieving the SDGs, as they are not only medical tools but foundation stones of public health, economic stability and modern societal development. Antibiotics play a dual-faceted role as essential tools for achieving several goals and as a dwindling resource whose loss to antimicrobial resistance (AMR) poses an existential risk to the entire 2030 agenda of the UN.

### 10.1. Role of Antibiotics in SDGs 1 and 8: No Poverty and Decent Work and Economic Growth

AMR has emerged as a global concern due to the rapid growth of AMR infections and the insufficiency of new antimicrobial drugs [146]. The contribution of antibiotics to SDG 1 lies with their functions to prevent pernicious health expenses that exacerbate poverty, especially in low- and middle-income countries where infectious diseases distinctively burden the poor. For the affected households, these sudden high expenses can be disastrous, forcing them to sell their assets for medical expenses or take on debt that triggers a cycle, pushing them further into poverty [147].

In agriculture, antibiotics are predominantly used to treat diseases, while in livestock and animal husbandry, they are used to promote growth by fighting infections. Overuse or unsupervised use of antibiotics in agriculture and livestock leads to AMR. Losing the effectiveness of antibiotics due to AMR results in agricultural and livestock losses, and the alternative treatments are often expensive or ineffective. This negatively impacts economic growth [148]. The role of antibiotics in supporting SDG 8 is through maintaining a healthy workforce and productivity. Effective usage of antibiotics enables the treatment of bacterial infections, reducing illness duration, morbidity, and mortality. This supports workforce health, minimizes absenteeism, and sustains labor productivity; key elements of economic output and decent work [149].

In sectors such as healthcare, agriculture, livestock farming, and food production, responsible antibiotic use prevents disease outbreaks that could disrupt employment and livelihoods, particularly in low- and middle-income countries where these sectors drive jobs and growth [147,150]. Antibiotics support modern economic systems by facilitating safe medical procedures, animal health, and food security, which indirectly help sustainable economic productivity. However, the growing threat of AMR, pushed by overuse, misuse, and misadministration, compromises the achievements of antibiotics. AMR increases treatment failure, lengthens sickness and increases treatment expenditures, resulting in the absence of workers, reduced productivity and efficiency, and loss of income [151]. According to reports, uncontrolled AMR could lower global GDP by 1–3.8% by 2050 and could result in yearly losses of 1–3.4 trillion US$ by 2030. The effects on the countries with lower economic capacity can be much harsher [147]. This affects decent employment possibilities and weakens sustained economic growth, particularly in labor-intensive industries like agriculture, where farmers’ livelihoods are impacted by persistent diseases in cattle [149,150]. Rising AMR from animal usage of antibiotics may negatively impact farmers’ and food producers’ revenue potentials in agriculture and food industries, which contradicts SDG8′s commitment to productive employment [147]. To maintain progress in SDG 8 goals, the management of antibiotics, the prevention of infections, One-Health strategies and the development of alternatives are crucial for maintaining antibiotic efficacy [151].

### 10.2. Role of Antibiotics in SDG 2: Zero Hunger

Antibiotics are widely used in agriculture and livestock to prevent and treat illnesses, improve health and wellbeing, and enhance feed efficiency, which collectively contribute to higher agricultural output and food security. In compliance with the SDG2 goals, including doubling agricultural productivity and small-scale producers’ income by 2030, this use of antibiotics helps to sustain herd sizes, improve farms’ financial stability, and fulfill the rising demand for animal proteins worldwide [147,150]. Increased output in the food-animal industry is expected to result in a two-thirds increase in global antibiotic usage between 2010 and 2030, compromising the goal of SDG 2′s resilient and sustainable farming practices. AMR can transmit through the food chain, soil and environment, endangering food safety, farmers’ livelihoods, and long-term productivity. In the United States, up to 80% of antibiotic usage is in animal feeds, surpassing their use for human medical purposes and leading to AMR [147,149]. Experts advise moving toward sensible antibiotic use in animals used for food production and incorporating it into SDG 2′s definition of sustainable practices, such as enhanced animal husbandry, prohibitions on non-therapeutic uses, and monitoring indicators to reduce risks while retaining benefits. Evidence from countries that have outlawed growth-promoting antibiotics suggests that, if priority is given to disease prevention measures, high output can be sustained without suffering significant financial losses. To avoid AMR from undermining the objectives of SDG2, regulatory frameworks are necessary to balance the role of antibiotics [149,150].

### 10.3. Role of Antibiotics in SDG 3: Good Health and Well-Being

SDG 3 is directly supported by the prevention and treatment of infectious diseases, which is made possible by effective antibiotics. By guaranteeing access to safe, effective, high-quality and reasonably priced essential medications, they enable reductions in maternal and child mortality and aid in the fight against infectious disease epidemics like tuberculosis and HIV, cancer treatments, surgical procedures and universal health coverage (UHC). Many common medical procedures become risky or difficult in the absence of adequate antibiotics, increasing mortality and morbidity. Without effective antibiotics, it would be impossible to meet targets of SDG 3, especially in low- and middle-income nations where the burden of infectious disease is highest [149]. AMR might undermine advancements in maternal health, child survival and the control of infectious diseases. It also increases treatment failures, prolonged illnesses, and increased expenses. It increases health system vulnerabilities and puts UHC at risk [152]. The United Nations has acknowledged the link to AMR and has focused on AMR-specific monitoring under SDG3. A percentage of health facilities have access to several core tracer medicines, including several from the WHO AWaRE classification of antibiotics, which emphasizes sustainable access balanced with governance to prevent further resistance [153]. This assists in tracking resistance and supports early warning and management of health risks (UN Statistics Division, metadata for SDG 3.d.2). Promoting infection control and unprejudiced health outcomes, antibiotics are essential for achieving SDG 3, but their efficacy must be maintained through careful usage, monitoring and innovation to ensure continued advances in global health [154].

### 10.4. Role of Antibiotics in SDG 6: Clean Water and Sanitation

Wastewater from pharmaceuticals and hospital waste contributes to the amount of antibiotics used, introducing antibiotics into water systems, leading to pollution that compromises water quality and endangers aquatic ecosystems and safe drinking water [155,156]. As a result, poor water, sanitation, and hygiene infrastructure worsens infection rates, especially for respiratory and diarrheal infections, which again leads to increased antibiotic use and promotes the chain reaction of AMR spread through contaminated surroundings [155]. SDG 6′s advancements in solving poor water, sanitation, and hygiene infrastructure can decrease pathogen exposure and infection incidences, counteract the AMR cycle and reduce the demand for antibiotics. Integrated water, sanitation, handwashing and nutritional interventions have been demonstrated to reduce child antibiotic use by 10–14% in countries like Bangladesh [157]. To support broader SDG progress, water management should be strengthened beyond traditional water treatment procedures and by treating environmental waters as AMR reservoirs [156].

### 10.5. Role of Antibiotics in SDG 10: Reduced Inequalities

Poor living conditions, limited access to healthcare, and insufficient preventative measures put underprivileged communities at higher risk for infectious diseases [147,155]. While resistance infections disproportionately impact refugees, undocumented people and those without insurance who frequently turn to self-medication, antibiotic consumption is unequal worldwide, with lower per capita use in many low- and middle-income countries despite high disease burdens [150]. SDG 10’s targets to empower social and economic inclusion and reduce the inequalities of these outcomes are directly linked to AMR’s deterioration of economic productivity. Addressing these dynamics requires integrating AMR mitigation into broader efforts to promote equitable resource distribution, inclusive healthcare, and targeted policies for vulnerable groups to prevent AMR from reversing development gains [147,155].

### 10.6. Role of Antibiotics in SDG 13: Climate Action

Climate change is a major driver and amplifier of AMR, whereas AMR mitigation can support climate-resilient strategies. A 1 °C increase in ambient temperature is associated with a 2–4% increase in antibiotic resistance among significant pathogens like *Escherichia coli*, *Klebsiella pneumoniae* and *Staphylococcus aureus*, as well as a 5–10% increase in foodborne disease cases [147,150]. Changed ecosystems, water pollution, population migration and extreme weather events interrupt healthcare systems, promote antibiotic overuse and increase the transmission of resistant pathogens. This leads to the generation of synergies that aggravate AMR expansion worldwide [158]. Lowering infection burdens, reducing antibiotic demand, and addressing common drivers like pollution and biodiversity loss can help to address the targets of SDG 13, like lowering greenhouse gas emissions, promoting sustainable agriculture and improving environmental management, leading to the prevention of AMR. Integrating One Health approaches that combine AMR control with climate adaptation policies offer positive synergies and can reduce AMR burdens with greater efficiency than isolated measures [158,159]. While coordinated efforts to address both threats will strengthen global commitments to SDGs, neglecting the interconnections between AMR and climate will risk SDG 13 progress [147].

### 10.7. Role of Antibiotics in SDG 17: Partnership

Effective partnerships are crucial for surveillance, management, research and development, technology transfer and universal access to antimicrobials, as AMR is inevitably a multisectoral, transboundary issue that requires coordinated action from different sectors [147,158]. SDG 17 is put into practice by the Quadripartite collaboration, consisting of the World Health Organization (WHO), the Food and Agriculture Organization (FAO), the United Nations Environment Program (UNEP) and the World Organization for Animal Health (WOAH). It promotes a One Health approach through the AMR multi-stakeholder partnership platform, shared strategies, and support for national action plans in countries to bring together AMR efforts with the 2030 agenda [147]. The scope of SDG 17 could be expanded further to support global initiatives such as improved surveillance, management, new vaccines and antibiotics, and resource mobilization in countries where limited capacities result in high AMR burdens [147,159]. Public–private partnerships such as Global Antibiotic Research & Development Partnership (GARDP) and CARB-X accelerate R&D and access, whereas government commitments like United Nations resolutions and efforts on inclusive collaborations between government, academia and private sectors can expedite the mitigation of AMR and the progress of SDGs [147,150]. SDG 17 can serve as a vital framework for transforming collaborations into practical instruments to manage AMR.

## 11. Summary and Future Prospective

This meta-analysis provides a critical assessment of electrochemical biosensors based on graphene-based materials for the detection of antibiotics in food products. Graphene-based materials (GR, GO, RGO) demonstrate excellent analytical performance (LODs from µM to sub-fM); however, a major gap remains between laboratory innovation and field deployability. This systematic review highlights that RGO-based biosensors appear to be able to demonstrate the lowest median LODs because of their synergistic conductivity and functionalization, whereas GO-functionalized screen-printed electrodes are superior when considering the trade-off for field application, even though they show relatively low sensitivity.

Furthermore, the applicability of these devices in terms of POC applications is limited because of numerous practical challenges. Only 12.8 percent of the assessed sensors meet all the POC requirements, and only 19.1 percent of the assessed sensors utilize fabrication methods consistent with large-scale production. Another critical challenge facing the development of these devices is the cost, as 61.7 percent of the assessed studies utilize expensive components (noble metal nanoparticles, rare earth elements) for the recognition of biological molecules. Finally, most of the reviewed sensor platforms exhibit limitations related to long-term stability, matrix effects in complex matrices (milk, wastewater, serum), and the necessity for extensive sample preparation prior to analysis.

The quantitative meta-analysis shows that graphene-based sensors have pooled detection limits in the range of 0.007 to 0.272 µM. However, due to large between-study heterogeneity (I^2^ approximately 76%) and large prediction intervals, there is significant variability in sensor performance among labs. As such, GRADE assessment rates the certainty of these estimates as low to very low. This indicates that while current proofs of concept for most benchmarking studies are adequate, they do not provide sufficient assurance regarding reliable on-site use. Notably, phenicol detection using noble metal surfaces and nitrofurans detection using metal oxide surfaces were found to be the best-performing combinations of materials and antibiotics. Therefore, bridging this translational gap will require standardized reporting of both the stability and interfering threshold levels, standardization of fabrication methods, and a targeted strategy aimed at utilizing earth-abundant nanomaterials to allow for scalable and affordable antimicrobial monitoring.

In addition to evaluating the technical aspects of these biosensors, the systematic review emphasizes the importance of antibiotic residue monitoring in the achievement of the United Nations’ Sustainable Development Goals (SDGs), particularly SDG 3 (Good Health and Well-being), SDG 6 (Clean Water and Sanitation), and SDG 1/8/10 (Poverty, Economic Growth, and Reduced Inequality). It has been recognized that effective monitoring of antibiotic residues is necessary to combat AMR and protect the human microbiome, as well as to prevent economic losses resulting from drug-resistant infections. Therefore, the development of graphene-based biosensors should move away from being focused on demonstrating ultrasensitive detection capabilities in controlled environments and toward focusing on developing sensors that are robust, affordable, and accessible to users in resource-poor environments.

Future research should focus on key strategic areas to bridge the gap between proof-of-concept in an academic environment and a useful tool for solving real-world problems:

- The use of expensive precious metals (Au, Pt) and rare earth dopants in current designs for graphene-based biosensor technology needs to be replaced by less expensive materials that are abundant on Earth (Fe, Mn, Zn oxides) or by using direct electrocatalytic reactions that do not require additional chemicals. The cost of materials should be below $5 per device so that biosensors may be used in resource-poor settings and in large-scale networks to monitor environmental pollution.
- Future research should be focused on scalable fabrication techniques such as screen-printing, laser-induced graphene, and roll-to-roll printing. Additionally, integration into portable electronic systems such as smartphone-based electronics and microfluidic sample-to-answer systems is required to remove the need for a large electrochemical workstation and skilled operators.
- Using Artificial Intelligence (AI) and Machine Learning (ML) is a key move to solve the translation problems found in the study, especially those related to accuracy in complicated mixtures and the ability to use tests at the POC. AI-assisted signal processing will be a useful way to enhance accuracy by recognizing specific antibiotic targets in complex samples without needing a lot of preparation. Additionally, the incorporation of graphene-based transducers coupled with smartphone interfaces will enable ML to automatically interpret data collected by the sensors, adjust for sensor drift, and reduce the technical expertise needed for sensor deployment at the POC. Using AI for both predictive material design and real-time data analysis, the next generation of biosensors will be able to transition from laboratory-sensitive devices to intelligent, scalable monitoring systems that support the global fight against antimicrobial resistance and contribute to sustainable development goals.
- Next-generation graphene sensors will be transitioned from standalone devices to nodes in distributed systems of intelligence. Machine learning models (e.g., random forest, CNNs) for voltammogram analysis can deconvolute overlapping redox peaks, correct for matrix-induced baseline drift and predict concentrations without extensive calibration. Combined with low-power microcontrollers (ESP32, Arduino) and Bluetooth/Wi-Fi modules, these sensors enhance real-time data streaming to cloud dashboards. This allows spatial-timely mapping of antibiotic residues in watersheds/livestock farms/clinical settings, which feed into predictive outbreak models for antimicrobial resistance. Edge-AI implementations will also further reduce latency, allowing on-device decision-making capabilities for resource-limited users.
- To establish a level of comparison and reliability in the evaluation of POC devices, future studies must follow standard protocols for validating POC devices (the WHO criteria). These protocols include: (a) testing in real-world matrices with little or no pretreatment; (b) reporting results of the longevity of devices (>30 days at ambient temperature); and (c) interference studies of compounds similar in structure to those being tested.
- Biosensors will have limited life spans if they are fouled with biological material in real-world applications (blood, urine, soil). Therefore, surface engineering techniques such as the use of antifouling coatings and regenerable architecture designs must be developed to extend the life span of sensors and reduce the frequency of replacing them. Reducing the cost of operation of biosensors will increase their utility and availability for use in developing countries.
- Future biosensor design should take into account the needs of sustainable development. Therefore, designers of biosensors should partner with users of the devices (farmers, clinicians, environmental inspectors) to develop devices that meet their needs (water quality monitoring, food safety monitoring, antibiotic resistance monitoring). Increasing access to diagnostic technologies has been identified as an important strategy to limit the inequitable burden of AMR on the most vulnerable populations in the world.

Addressing these challenges will enable the next generation of graphene-based biosensors to go from being high-performance laboratory curiosities to being valuable tools for improving global health security and the sustainability of the environment.

## Figures and Tables

**Table 1:** Electrochemical determination of antibiotics on graphene-based sensors and biosensors in food, the environment and pharmaceuticals.

Antibiotic	Electrode Substrate	Sensing Materials	Detection Time	LDR (µM)	LOD (µM)	Medium (pH)	Analytical Characteristics		Sensor Characteristics		Analytical Performance Metrics	Real Sample	Ref.
							Adv.	Disadv.	Adv.	Disadv.			
Amoxicillin	GCE	CVD Gr/AuNP/Lac	CA (720 s)	0.425–292	0.425	PBS (10)	L, M, N, O	–	A, B, C, D	X, Y, Z	^(1)^ 5 d. 4 °C, ^(2)^ 2040 µA. µM^−1^.cm^2^, ^(4)^ RSD 1.37%	PBS	[53]
Ampicillin	SPCE	GA-NH-YN	SWV (420 s)	0.01–10,000	0.00136	PBS (7.4)	L, M, N, O	–	A, C, D, F, J	X	^(1)^ 4 w. 95% ^(5)^ 88.2–104%	Tap Water, Milk, Saliva	[78]
Azithromycin	Ni foam	MACGS	CV (2 s)	1–5	0.5	PBS (7)	L, N, O	Q	A, C, D, F	U, X, Z	^(1)^ 90 d. 20–100 °C 95.7%	Buffer	[79]
	CPE	GNR-CoFe_2_O_4_@NiO, HMIM PF_6_	SWV (34 s)	10–2000	0.66	PBS (7)	L, M, N, O	–	A, B, C, D, F	X, Z	^(1)^ 30 d., RSD 3.15%, ^(3)^ RSD 3.15%, ^(4)^ RSD 2.5%, ^(5)^ 95.26–99.23%, ^(6)^ 400-fold	Urine, Pharmaceutical Capsule	[80]
Cephalexin	FTO	GQD/CFX-BSA Abs	DPV (120 s)	1 × 10^−6^–1	5.3 × 10^−10^	PBS (7.5)	L, M, N, O	–	A, B, C, D, F	X, Z	^(1)^ 28 d. 4 °C, 5 cyc., ^(4)^ SD = 498 μA	Milk, Egg, Meat, Buffer	[46]
Chloramphenicol	GCE	Ti_3_C_2_T_x_@Au–AS-GQD aerogel	DPV (92 s)	0.003–100	0.0012	PBS (7)	L, M, N, O	–	A, B, D, F, J	X	^(1)^ 49 d. 2.6% RSD, ^(3)^ 2.2% RSD, ^(4)^ 1.5%, ^(5)^ 96.5–99.5%	Milk	[84]
	CE	β-CD@G/Cu-BTC	DPV (64 s)	0.01–100	0.0031	PBS (7)	L, M, N, O	–	A, B, C, D, J	X, Y, Z	^(5)^ 95.4–106.0%	Milk	[56]
	MSPE	MIO@NG	SWV (210 s)	0.01–300	0.01	PBS (7)	L, M, N, O	–	A, B, C, D, F, J	X, Y, Z	^(1)^ 4 w. 97.5%, ^(3)^ 2.26% RSD, ^(5)^ 97.8–106.5% RSD < 4%, ^(6)^ RSD < 6%	Fresh Milk, Milk Powder, Eye Drops	[67]
	PI film	LEG	DPV (96 s)	10–200	20	PBS (7)	M, N, O	P	C, D, E, J	T, Y	^(2)^ 0.2693 μA.μM^−1^, ^(3)^ 1.41–1.88% RSD, ^(5)^ RSD 0.77–0.87%	Pork, Milk	[83]
	AuE	PDDA-Gr/PtPd@Ni-Co HNBs	DPV (5.4 h)	10^−8^–0.01	9.85 × 10^−10^	PBS (–)	L, M, N, O	–	A, B, C, D, F	X, Y, Z	^(1)^ 14 d. 91%, ^(3)^ 3.63% RSD, ^(5)^ 93–99% RSD < 5%	Honey	[81]
Ciprofloxacin	GCE	Graphene (EGr)	^w^ LSV, ^x^ CA (^w^ 560 s; ^x^ 1210 s)	^w^ 0.06–100 ^x^ 0.0033–25	^w^ 0.0182 ^x^ 0.001	PBS (6)	L, M, N, O	–	A, B, C, D, F, J	X	^(1)^ 15 d. 93.1–94.2%, ^(3)^ 3.7–5.1% RSD, ^(5)^ 95.0–101.2%	Wastewater, Pharmaceuticals	[52]
	CP	SDS/Gr	DPV (168 s)	0.3–100	0.029	PBS (4.2)	L, M, N, O	–	A, B, C, D, E, F, J	–	^(1)^ 15 d., ^(3)^ 3.19% RSD, ^(5)^ 93.3–98.7%	Tablet, Urine	[85]
	CPE	GR NSs, Fe_3_O_4_ NPs	DPV (144 s)	0.01–20	0.0018	PBS (3)	L, M, N, O	–	A, B, C, D, E, F, J	–	^(1)^ 93.1% after 1 m., ^(2)^ 0.095 A⋅M^−1^; 47.91 µA µM^−1^ cm^−2^, ^(3)^ 4.31% RSD, ^(4)^ 1.6% RSD, ^(5)^ 96.8–103%	Tap Water, River Water, Antibiotic Plant Effluent	[86]
^k^ Chloramphenicol ^l^ Florfenicol	GCE	GR/CuPc NCs	DPV	^k^ 0.1–20 ^l^ 1.0–30	^k^ 0.027 ^l^ 0.75	PBS (7.4)	L, M, N, O	–	A, B, C, D, F	X	^(1)^ 1 m. 25 °C 90.1%, ^(3)^ RSD ^k^ 6.70%, ^l^ 9.76%, ^(5) k^ 97.8–101.9%, ^l^ 98.2–99.0%	Eye Drops, Milk	[87]
^k^ Chloremphenicol ^m^ Ciprofloxacin	GCE	Au/C_3_N_4_/GN	SWV	^k^ 0.7–120 ^m^ 0.6–120	^k^ 0.027 ^m^ 0.24	PBS (7)	L, M, O	–	A, B, D, F	X, Z	^(1)^ 15 d. 93.1–94.2%, ^(3)^ 3.7-5.1% RSD, ^(5)^ 95.0–101.2%	Milk	[82]
^i^ Linezolid ^j^ Meropenem	GCE	Gr/CNT-HQ/Fe-Ni	DPV, CV	^i^ 0.007–12 ^j^ 0.01–12	^i^ 0.00097 ^j^ 0.0013	PBS (3.0)	L, M, N, O	–	A, B, C, D, F	X	^(1)^ 1 m. 93.5%, ^(2) i^ 2.9 and ^j^ 2.3 μA.μM^−1^, ^(3)^ RSD 0.63–1.0% intra-day; inter-day, RSD < 2.34% (25 cyc.), ^(5)^ 97–103%, ^(6)^ 100-fold	Human Serum	[91]
Kanamycin	GCE	ssDNA, CdS/CHIT	DPV (200 s)	202–2020	60.6	PBS (7)	O	P, Q, R	D, E, J	T, U, V	^(3)^ RSD 2.4%	Aqueous Solution	[88]
Metronidazole	GCE	GR/Fe_3_O_4_NPs	DPV (128 s)	0.05–5; 5–120	0.00023	PBS (6)	L, M, N, O	–	A, B, C, D, F, J	X	^(1)^ 4 w. 91.8%, ^(2)^ 7.34 μA.μM^−1^ cm^−2^, ^(4)^ 3.4% RSD, ^(5)^ 94.3–102.5%, ^(6)^ RSD < 5%	Antibiotic Plant Effluent, Tap/River Water	[89]
Nitrofurantoin	GCE	LuVO_4_/GRS	i-t AMP (120 s)	0.008–256	0.001	PBS (7)	L, M, N, O	–	A, B, C, D, F	X	^(1)^ 3800 s 97.4%; 98.7% 20 cyc., ^(2)^ 1.709 μA μM^−1^ cm^−2^, ^(3)^ 2.25% RSD, ^(5)^ 95–105%	Environmental Water	[90]
Ornidazole	GCE/SPCE	MnMoO_4_/GNS	i-t AMP (5 s)	0.01–770	0.000845	PBS (7)	L, M, N	S	A, B, C, E, F, J	X, Y	^(1)^ 2500 s 94.6%, ^(2)^ 104.62 mA mM^−1^ cm^−2^	River Water, Human Urine	[92]
Sulfamethazine	GCE	MWCNTs/GQDs	SWV (300 s)	0.5–10; 10–200	0.068	ABS (9)	L, M, N, O	–	A, B, C, D	X, Y	^(1)^ 5 d., 10 cyc., ^(5)^ 95.4–104.8% RSD < 4.14%	Aquaculture Seawater	[93]
Tetracyclin	SPCE	Graphene	DPV (144 s)	1–100	0.08	PBS (7)	L, M, N, O	–	A, B, C, D, E, F, J	–	^(1)^ 1 m. 98%, ^(3)^ RSD < 3%, ^(4)^ 2.3% RSD, ^(5)^ 98–102%	Milk, Honey	[95]
^e^ Trimethoprim ^f^ Sulfamethoxazole	Parchment paper	rGNR	DPV (140 s)	^e,f^ 1–12	^e^ 0.09 ^f^ 0.04	PBS (7)	L, N, O	Q	A, C, D, F, J	X	^(3)^ 4.42–4.47% RSDs, ^(4)^ 2.76–3.53% RSDs, ^(5)^ 7.8–102.0% (RSD < 4%)	Tap Water	[94]

^e^—Trimethoprim; ^f^—Sulfamethoxazole; ^i^—Linezolid; ^j^—Meropenem; ^k^—Chloramphenicol; ^l^—Florfenicol; ^m^—Ciprofloxacin; ^w^—LSV; ^x^—CA. Analytical Characteristics—Advantages: (L) low LOD; (M) wide LDR; (N) selective electrode; (O) high reproducibility. Disadvantages: (P) high LOD; (Q) narrow LDR; (R) non-selective electrode; (S) low reproducibility of sensor; (H) sensor repeatability. Sensor Characteristics—Advantages: (A) low LOD; (B) wide LDR; (C) selective electrode; (D) high reproducibility; (E) easy modifier reparation; (F) long-term stability; (J) low cost. Disadvantages: (T) high LOD; (U) narrow LDR; (V) non-selective electrode; (W) low reproducibility of sensor; (X) complex modifier preparation; (Y) short-term stability; (Z) high cost. Analytical Performance Metrics: ^(1)^—Stability; ^(2)^—Sensitivity; ^(3)^—Reproducibility; ^(4)^—Repeatability; ^(5)^—Recoveries; ^(6)^—Selectivity/Specificity. d./w./m./cyc.—day(s)/week(s)/month(s)/cycle(s); RSD—Relative Standard Deviation.

**Table 2:** Electrochemical determination of antibiotics on graphene oxide-based sensors and biosensors in food, the environment and pharmaceuticals.

Antibiotic	Electrode Substrate	Sensing Materials	Detection Time	LDR (µM)	LOD (µM)	Medium (pH)	Analytical Characteristics		Sensor Characteristics		Analytical Performance Metrics	Real Sample	Ref.
							Adv.	Disadv.	Adv.	Disadv.			
Aminoglycosides	SPCE	OMC/Aptamer	^y^ DPV/^z^ CV (112 s)	0.01–1	0.00247	KCl (7.6)	L, N, O	Q	A, C, D, F, J	U, X	^(1)^ 2 w. at 4 °C 94.68% RSD 4.37%, ^(3)^ 2.60% RSD, ^(5)^ 91.44–103.44%,	Milk	[43]
Benzylpenicillin	CPE	GO/MnFe_2_O_4_	DPV (16 s)	1–100; 100–1000	0.145	PBS (7)	L, M, N, O	–	A, B, C, D, E, F, J	–	^(1)^ 1 m. 97.5%, ^(2)^ 15.16 µA·µM^−1^·cm^−2^, ^(3)^ RSD 5.41%, ^(5)^ 96.5–107.6%	Tap Water, Mineral Water, River Water	[45]
Cefixime	GCE	EMIP/GNU/GO	DPV	0.02–0.95	0.0071	PBS (7)	L, M, N, O	–	A, B, C, D, F	X	^(1)^ 87.3% 15 d., ^(2)^ 1.3 nA·nM^−1^·cm^−2^, ^(3)^ RSD < 0.98%, ^(4)^ RSD 2.76%, ^(5)^ 97.8–102.9%	Human Serum, Urine	[44]
Chloramphenicol	GCE	LCSs/GO@MnO_2_	DPV/CV (120 s)	0.017–477.247	0.0012	PBS (7)	L, M, N, O	–	A, B, C, D, E, F, J	–	^(2)^ 105.22 μA, μM^−1^ cm^−2^, ^(3)^ 1.97% RSD (CV); 2.08% RSD (DPV), ^(5)^ 84.59%	Milk	[60]
	GCE	GdSnO/GO	DPV, CV (140 s)	0.02−522.43	0.098	PBS (7)	L, M, N, O	–	A, B, C, D, F	X, Z	^(1)^ 20 d. 95.89%, ^(2)^ 0.41 μA⋅μM^−1^ cm^−2^, ^(3)^ 2.86% RSD, ^(4)^ 2.57% RSD, ^(5)^ 97.85–102.86%	Milk, Honey	[55]
	GCE	SrTiO_3_/GO	LSV (304 s)	10–100; 100–550	6.08	PBS (7)	L, M, N, O	–	A, B, C, D, F	X, Y	^(1)^ 15 d. 82.06%, ^(2)^ 2.771 µA⋅μM^−1^⋅cm^−2^, ^(3)^ 3.42% RSD	Milk, Honey	[97]
	SPE	MoS_2_/SPE (0: 1); (1: 0.1); (1: 1.5); (1: 2)	DPV, CV (300 s)	0.5–50; 0.1–50; 0.1–50; 0.1–50	0.061; 0.070; 0.060; 0.050	KCl (5)	L, N, O	Q	A, D, F, J	U, X, Y	^(1)^ 30 d. 75%, ^(2)^ 0.14–0.62 0.54 mA⋅mM^−1^, ^(3)^ 2.14% RSD, ^(4)^ 1.1–2.27% RSDs, ^(5)^ 85–98% recoveries	Pork, Chicken	[101]
	GCE	GO/MWCNT	CV (520 s)	15–600	0.204	PBS (7)	L, M, N, O	–	A, B, C, D, E, F, J	–	^(1)^ 21 d. 90%, ^(2)^ 1.71 µA⋅µM^−1^ cm^−2^, ^(3)^ RSD < 3%, ^(4)^ 3–5%, ^(6)^ matrix effects	Milk, Tap Water, Eye Drops, Capsules, Human Blood Serum	[96]
	GCE	MoS_2_-IL/GO	DPV (420 s)	0.1–400	0.047	PBS (7)	L, M, N, O, H	–	A, B, C, D, F, J	X	^(1)^ 10 d. 92.4%, ^(2)^ 0.302 µA.µM^−1^, ^(3)^ 2.88% RSD, ^(4)^ 2.60% RSD, ^(5)^ 90.7–100%	Eye Drops, Milk, Urine	[99]
	GCE	GO/ZnO NCs	DPV (272 s)	0.212–124.712	0.01	PBS (7)	L, M, N, O	–	A, B, C, D, E, F, J	–	^(1)^ 24 d. 93.89%, ^(2)^ 7.27 µA.µM^−1^.cm^−2^, ^(3)^ 2.84% RSD, ^(4)^ 3.24% RSD, ^(5)^ 97.53–102.53%	PBS, Honey, Milk, Eye Drops	[98]
	GCE	GO/SmMoSe_2_	LSV (480 s)	0.01–244	0.005	PBS (7)	L, M, N, O	–	A, B, C, D, F	X, Z	^(1)^ 7 d. 94.1%, ^(2)^ 20.6 µA.µM^−1^.cm^−2^, ^(3)^ 3.8% RSD, ^(5)^ 96–102% recoveries, ^(6)^ 100-fold	Spiked Milk	[75]
	GCE	Pd NPs/GO	i-t AMP (50 s)	0.007–102.68	0.001	PBS (7)	L, M, N, O	–	A, B, C, D, E, F	Z	^(1)^ 97.6% (1500 s), ^(2)^ 3.0479 µA.µM^−1^.cm^−2^, ^(3)^ 1.65% RSD, ^(4)^ 3.48% RSD, ^(5)^ 95.0–110.0%	Milk, Urine	[100]
Cloxacillin	SPCE	AuNR/GO	DPV	0.005–0.775	0.0016	PBS (7)	L, M, N, O	–	A, B, C, D, E, F, J	–	^(1)^ 12 d. 25 °C; 94.7%, ^(2)^ 0.5604 µA.nM, ^(3)^ 4.3% RSD, ^(4)^ RSD 3.9–6.1%	Aqueous Solution	[102]
Furazolidone	GCE	CeW/GO	DPV (210 s)	1–54; 54–260	0.054	PBS (7)	L, M, N, O	–	A, B, C, D, E, F, J	X	^(1)^ 2 w. 90.2%, ^(2)^ 2.5 μA.μM^−1^.cm^−2^, ^(3)^ 2.8% RSD, ^(5)^ 98.4–99.6%	Human Urine	[103]
^g^ Levofloxacin ^h^ Acetaminophen	GCE	CNNS/GO	DPV (^g^ 480 s; ^h^ 180 s)	^g^ 0.5–15 ^h^ 0.5–50	^g^ 0.079 ^h^ 0.017	PBS (5)	L, N, O	H, Q, S	A, D, E, J	U, X	^(5)^ 95.6–111.8%	River Water	[104]
Penicillins	GCE	GO-SELEX	DPV (2 h)	0.002–10	0.000667	PBS (7.5)	L, M, N, O	–	A, B, C, D, F, J	X	^(1)^14 d. 93.11%, RSD 2.92%, ^(3)^ RSD 2.13%, ^(5)^ 94.27–105.66%,	Milk	[110]
Sulfadiazine	GCE	GdVO_4_@GO	i-t AMP	0.02–145.5; 145.5–945.5	0.00319	PBS (7)	L, M, N, O	–	A, B, C, D, F	X, Z	^(1)^ 1700 s, ^(2)^1.3009 μA.μM^−1^.cm^−2^, ^(3)^ 4.8% RSD, ^(4)^ 3.5% RSD, ^(5)^ 97.8–102.3%, ^(6)^ 10-fold 5% RSD	Human Serum, River Water, Wastewater	[74]
Sulfamethazine	GCE	Cu-Ag/GO	SWV (88 s)	10–1000	0.46	6	L, M, N, O	–	A, B, C, D	X, Y, Z	^(1)^ 1 w. 95.67%, ^(2)^ 0.029 µA.µM, ^(3)^ 2.79% RSD, ^(5)^ 96.9–103.67%	Cow Milk	[105]
Sulfamethoxazole	GCE	GO/ZnO NRs	DPV (100 s)	3.95 × 10^−6^–4.74 × 10^−5^	2.81 × 10^−4^	PBS (7.02)	L, N	Q	A, C	U, X	^(2)^ 98%	Environmental Groundwater	[68]
Sulfathiazole	GCE	HV@GO	CV DPV	0.01–36– 36–511	0.00294	PBS (7)	L, M, N, O	–	A, B, C, D, F	X	^(1)^ 1500 s, ^(2)^ 1.4147 µA.µM^−1^.cm^−2^, ^(3)^ 2.18% RSD, ^(4)^ 1.95% RSD, ^(5)^ 96.8–102.4%, ^(6)^ 10-fold	River Water, Urine	[76]
Tetracycline	Au	MIP/GO/Al_2_O_3_/Au	DPV (240 s)	0.001–0.06	0.00016	PBS (7)	L, M, O	R	A, B, C, D	X, Y	^(3)^ RSD < 8%, ^(5)^ 97.60–105.40%	Milk, Chicken Feed	[106]
	GCE	AuNPs/GO/TC-MIP	DPV, CV (200 s)	1.125–45	1.57 × 10^−3^	PBS (6)	L, M, N, O	–	A, B, C, D, F, J	X	^(1)^ 4 d. at 50 °C (RSD 3.1%), ^(3)^ RSD < 5%, ^(5)^ 81–102.6%	Milk	[107]
	Microfluidic Chip	CA-CNTs/CuS-GO	CV	0.0225–0.112	1.59 × 10^−4^	H_2_O (6)	L, N, O	Q	A, C, D, F	U, X, Z	^(3)^ 4.87% RSD, ^(4)^ 5.91% RSD, ^(5)^ 97.33–107.48%, ^(6)^ organic ≤ 12.48%, inorganic ≤ 8.68%	Environmental Water (Lake, River, Canal)	[108]
Tetracyclines	GCE	GO-SELEX (Apt/Fe/Zn-NaMMT/MWCNTs)	DPV	0.001–10	0.00046	KCl (7)	L, M, N, O	–	A, B, C, D, F, J	X	^(1)^ 2 w. 92.8%, ^(3)^ 2.38%, ^(4)^ 3.12–6.78%, ^(5)^ 98.26–104.15%	Milk (Fat/Protein Removed)	[111]
^e^ Trimethoprim ^f^ Sulfamethoxazole	GCE	GO-ZnO QDs Nanocomposite	SWV (96 s)	^e^ 0.172–1.03 ^f^ 0.197–1.18	^e^ 6.58 × 10^−2^ ^f^ 9.95 × 10^−7^	BR (7)	L, N	Q	A, C	X, Y	^(5)^ 98–102%	Tablet	[109]

^e^—Trimethoprim; ^f^—Sulfamethoxazole; ^g^—Levofloxacin; ^h^—Acetaminophen; ^y^—DPV; ^z^—CV. Analytical Characteristics—Advantages: (L) low LOD; (M) wide LDR; (N) selective electrode; (O) high reproducibility. Disadvantages: (P) high LOD; (Q) narrow LDR; (R) non-selective electrode; (S) low reproducibility of sensor; (H) sensor repeatability. Sensor Characteristics—Advantages: (A) low LOD; (B) wide LDR; (C) selective electrode; (D) high reproducibility; (E) easy modifier reparation; (F) long-term stability; (J) low cost. Disadvantages: (T) high LOD; (U) narrow LDR; (V) non-selective electrode; (W) low reproducibility of sensor; (X) complex modifier preparation; (Y) short-term stability; (Z) high cost. Analytical Performance Metrics: ^(1)^—Stability; ^(2)^—Sensitivity; ^(3)^—Reproducibility; ^(4)^—Repeatability; ^(5)^—Recoveries; ^(6)^—Selectivity/Specificity. d./w./m./cyc.—day(s)/week(s)/month(s)/cycle(s); RSD—Relative Standard Deviation.

**Table 3:** Electrochemical determination of antibiotics on reduced graphene oxide-based sensors and biosensors in food, the environment and pharmaceuticals.

Antibiotic	Electrode Substrate	Sensing Materials	Detection Time	LDR (µM)	LOD (µM)	Medium (pH)	Analytical Characteristics		Sensor Characteristics		Analytical Performance Metrics	Real Sample	Ref.
							Adv.	Disadv.	Adv.	Disadv.			
Amoxicillin	GCE	rGO/RuO_2_ NPs	DPV (500 s)	0.055–1.20	0.00163	PBS (7)	L, M, N, O	–	A, C, D	X, Y, Z	^(1)^ 1 m. 82%; 53% at 2m., ^(2)^ 2.55 µA.µM, ^(3)^ 4.57% RSD, ^(4)^ 2.94% RSD, ^(5)^ 96.0–101.1%	Urine	[113]
^a^ Amoxicillin, ^b^ Tetracycline	GCE	NiAl-LDH/rGO	DPV (90 s)	^a^ 0.62–8.1 ^b^ 0.6–5.6	^a^ 0.0043 ^b^ 0.0036	PBS (8)	L, N	S, H, Q	A, C, D	X, Y, Z	^(2) a^ 137.6 nA.µM^−1^.cm^−2^, ^b^ 161.4 nA.µM^−1^.cm^−2^	Tap water	[112]
Azithromycin	GCE	Ag_2_Se/β-CD/rGO	CV, DPV (660 s)	0.023–971.7	0.0045	PBS (7)	L, M, N, O	–	A, B, C, D, E, F, J	–	^(1)^ 20 d. stability, ^(2)^ 14.25 µA µM^−1^ cm^−2^, ^(3)^ RSD < 5%, ^(4)^ RSD < 5%, ^(5)^ 95.4–104.8%	Human Serum/Urine, Industrial River Water, Tablet	[66]
Ceftriaxone	SP PET	rGO/MoS_2_	DPV	1–100	0.28	PBS (2)	L, M, N, O	–	A, B, C, D, E, J	Y	^(2)^ 9.645 × 10^4^ µA.M^−1^, ^(3)^inter-day RSD 3.22%, ^(4)^intraday RSD 1.03%, ^(5)^92.2–112.6%	Surface Water	[115]
Chloramphenicol	SPCE	Sn/rGO	DPV (18 min)	0.5−30; 30−100	0.20	PBS (7)	L, M, N, O	–	A, B, C, D, E, J	Y	^(1)^ 1 w. 95.0% and 3 w. 75%, ^(2)^ 0.934–0.278 μA.μM^−1^.cm^−2^, ^(3)^ 0.87% RSD, ^(5)^ 96–104%	Milk, Honey, Eye Drops	[119]
	Au	PEI-rGO/AuNCs	DPV (20 s)	0.005–1	0.002	PBS (7.4)	L, M, N, O	–	A, B, C, D, F	X, Y	^(1)^ 9 d. 90.6%, ^(3)^ 2.36% RSD, ^(5)^ 97.7–109.36%	Chicken	[65]
	GCE	Fe/Al-MOF/rGO	DPV	20–1600	0.0015	PBS (7)	L, M, N, O	Q	A, B, D, F	X	^(1)^14 d., 2.5% RSD, ^(3)^ 1.59% RSD, ^(5)^ 94.3–98.7%, ^(6)^ TC tested	Lake Water	[72]
	GCE	rGO/Au/Co_2_CuS_4_	CA	0.007–0.141	0.0025	PBS & EA (7)	L, M, N, O	–	A, B, C, D, F	X	^(1)^ 33 d. 95%	Milk, Honey, Urine, Serum	[73]
	GCE	rGO/NiCo-BTC MOFs	DPV (90 s)	0.1−100	0.235	PBS (7)	L, M, N, O	–	A, B, C, F, J	X	^(1)^ 7 d. 93%, ^(2)^ 33.12 μA⋅μM^−1^⋅cm^−2^, ^(3)^ 8.5% RSD, ^(4)^ 4.4% RSD, ^(5)^ 97.79–100.07%	Tap Water	[135]
	GCE	Fe_3_O_4_/N-rGO	DPV (90 s)	1–200	0.03	PBS (7)	L, M, N, O	–	A, B, C, D, F, J	X	^(1)^ 20 d. 88.14%, ^(3,4)^ 2.36–3.21% RSDs, ^(5)^ 96.70–103.55%	Fresh Milk	[116]
	GCE	rGO@PDA@AuNPs	DPV (144 s)	0.1–100	0.058	PBS (7)	L, M, N, O	–	A, B, C, D, F	X, Y	^(1)^ 7 d. 94.5%, ^(3)^ 5.1% RSD, ^(4)^ 3.2% RSD, ^(5)^ 96.8–102.5%,	Milk	[62]
	GCE	MoS_2_-rGO; MoS_2_-MWCNTs; MoS_2_-CB	DPV (63 s; 70 s; 84 s)	5–35; 1–35; 5–55	1.0; 0.4; 1.9	PBS (7)	L, O	Q	A, B, D	X, Y, T, U, X	^(2,3)^ 3.581 μA.μM^−1^.cm^−2^, 3.07% RSD; 2.588 μA.μM^−1^.cm^−2^, 2.92% RSD; 1.235 μA.μM^−1^.cm^−2^, 2.21% RSD	PBS	[61]
	SPCE	Eu_2_O_3_/RGO	i-t AMP (80 s)	0.02–800.25	0.00132	PB (7)	L, M, N, O	–	A, B, C, D, E, F, J	–	^(1)^ 2250 s and 10 d. 96.8%, ^(2)^ 6.9055 µA. µM^−1^. cm^−2^, ^(3)^ 3.12% RSD, ^(4)^ 3.45% RSD, ^(5)^ 97.58–99.19%, ^(6)^ 10-fold	Fresh Milk, Honey	[77]
	GCE	Z-800/RGO	DPV (72 s)	1–180	0.25	PBS (7)	L, M, N, O	–	A, B, C, D, F, J	X, Z	^(1)^ 3 w. 90.2%, ^(2)^ 1.176 µA. µM^−1^, ^(3)^ 2.1% RSD, ^(5)^ 98–106%, ^(6)^ 10-fold	Milk, Honey	[120]
	GCE	Cl-RGO	DPV (450 s)	2–35	1	PBS (7.4)	N, O	P	C, D, E, F, J	T	^(1)^ 30 d., ^(2)^ 99.81%, ^(3)^ 1.5% RSD, ^(5)^ 98–105%	Milk, Calf Plasma, Tap Water, Eye Drops	[118]
	GCE	3DRGO	DPV (712 s)	1–113	0.15	PBS (7.4)	L, M, N, O	–	A, B, C, D, E, F, J	–	^(1)^ 30 d., ^(3)^ 0.85% RSD, ^(5)^ 97.3–104.6%	Eye Drops, Milk	[117]
Ciprofloxacin	SPCE	ERGO/PANI/PARS	LSV (432 s)	0.01–69.8	0.0021	BR (5)	L, M, N, O	–	A, B, C, D, J	X, Y	^(2)^ 5.09 μA.μM^−1^.cm^−2^, ^(3)^ 4.54% RSD, ^(4)^ 16.2% RSD, ^(5)^ 95.82–106.40%	Milk	[124]
	GCE	rGO/PEI/TiO_2_/Apt.	DPV	0.003–10.0	0.0007	TE (7)	L, M, N, O	P, Q	A, B, C, D, F, J	X	^(1)^ 10 d. 5 °C 96.6%, ^(3)^ 1.04% (n = 6), ^(4)^ 1.23% (n = 6) RSD, ^(5)^ 96.8–106.3%,	Lake Water	[136]
	GCE	Pt–RGO	DPV (72 s)	10–25	1.53	PBS (4)	N, O	P, Q	C, D, F	T, U, X, Z	^(1)^ 2 w. 92.8%, ^(5)^ 99.6–97.2% RSD (0.3-2.1%)	Tap Water, River Water	[123]
	CPE	N-prGO	DPV (128 s)	0.1–10	0.039	PBS (7.4)	L, N, O	Q	C, D, F, J	X	^(1)^ 2 w. 4 °C 92.7%, ^(2)^ 820 μA.mM^−1^, ^(3)^ 5.6% RSD, ^(5)^ 94.0–106%	Tablet, Eye Drops, Plasma	[121]
	SPCE	CNT@PSS-AuNPs/rGO, Aptamer	EIS (210 s)	0.012– 3.02	0.012	PBS (7.4)	L, M, N, O	–	A, B, C, D, F	X, Z	^(1)^ 7 d. 95%, ^(3)^ 1.4% RSD, ^(4)^ RSD < 1% (100 cyc.), 86–118%	Freshwater	[64]
	Graphite	Multilayered rGO	LS-AdSV (300 s)	0.5–20	0.078	PBS (2)	L, O	H, S	A, D, E, J	W, Y	^(1)^ 65% after 10 scans at 20 µM, ^(3)^ 1.50–5.81% RSD, ^(4)^ 4.93–14.8% RSD, ^(5)^ 94.2–106.3%	Lake Water	[47]
	GCE	NH_2_–UIO-66/RGO	ASV (2100 s)	0.02–1.0	0.00667	PBS (4)	L, M, N, O	–	A, B, C, D, F	X, Z	^(1)^ 7 d. 93.2%, ^(2)^ 10.86 µA. µM^−1^, ^(3)^ RSD 5.75%, ^(4)^ RSD 2.78%, ^(5)^ 96.8–108.6%	Tap Water, Lake Water	[71]
	GCE	Au NP-β-CD-RGO	DPV (360 s)	0.01–120	0.0027	PBS (4)	L, M, N, O	–	A, B, C, D, E, F, J	X	^(1)^ 7 d. 97.8%, ^(2)^ 0.1056 µA. µM^−1^, ^(3,4)^ 2.57% RSD, ^(5)^ 96.1–98.7%	Tap Water, PBS	[122]
Furaltadone	RRDE	FeVO/p-rGO NCs	i-t AMP (4 s)	0.5–84; 94–1319	0.138	PBS (7)	L, M, O	–	A, B, C, D, F, J	X, Y	^(1)^ 3500s stability, ^(2)^ 0.229 μA.μM^−1^.cm^−2^, ^(3)^ 1.75%, ^(4)^ 2.07% RSD, ^(5)^ 98.2–101.5%	Industrial Wastewater, Lake Water	[59]
Furazolione	SPCE	rGM-P	^y^ DPV, ^z^ CV (^y,z^ 280 s)	^y^ 0.2–78.2 ^z^ 0.04–200	^y^ 0.000275 ^z^ 0.00122	PBS (7)	L, M, N, O	–	A, B, C, D, F, J	X, Y	^(1)^ 15 d. 98.13%, 25 cyc. 90.5%, ^(3)^ 3.65% RSD, ^(4)^ 3.86% RSD	Tap Water, River Water	[125]
Gatifloxacin	GCE	Ag_2_S/RGO	LSV (52 s)	0.2−20; 20−250	0.0667	PBS (4)	L, M, N, O	–	A, B, C, D, F, J	X	^(1)^ 1 w. 97.5%, ^(2)^ 1.007 mA. mM^−1^.cm^−2^, ^(3)^ 1.9% RSD, ^(4)^ 3.5% RSD, ^(5)^91.8–102.4%	Stern, Crucian Carp, Chicken Meat	[48]
Kasugamycin	GCE	fMWCNTs/rGO/poly(SER)	DPV (120 s)	7.22–255	0.96	BR (6)	L, M, N, O	–	A, B, C, D, J	X, Y	^(1)^ 45 c. stability, ^(3)^ 2.49% RSD, ^(4)^ 2.22% RSD, ^(5)^ 95.5–100.1%	Cucumber, Zucchini, Carrot	[126]
Levofloxacin	3D-CB/PLA	rGO	DPV (16 s)	10–50	2.17	BR (6)	L	S, Q	A, C, D, F, J	U, X	^(5)^ 95–105%	Pharmaceuticals, Synthetic Urine, Tap Water	[49]
Linezolid	PtE	NiO/rGO	DPV, CV (200 s)	0.001–90	0.0031	PBS (6)	L, M, N, O	P, Q	A, B, C, D, F	X, Y, Z	^(5)^ 98.1−103.9%	Urine, Tablet	[70]
Metronidazole	GCE	FeMn_2_O_4_-rGO	DPV, CV (192 s)	0.01–512.2	0.0129	PBS (7)	L, M, N, O	–	A, B, C, D, E, F, J	X	^(1)^ 30 d. at 5 °C 4.81% and 2.81%, ^(2)^ 71.4 μA. mM^−1^. cm^−2^, ^(4)^ 98.94%	Urine, Serum, Tablet	[57]
	GCE	TA-Au-Ag-ANpM/r-GO/poly(glycine)	SWV (165 s)	0.000002–410	3.12 × 10^−8^	H_2_SO_4_ (2)	L, M, N, O	–	A, B, C, D, F	X, Z	^(1)^ 2 w. at 4 °C 95.6%, ^(3)^ 3.2% RSD, ^(4)^ 2.43% RSD, ^(5)^ 96.9–101.4%, ^(6)^ RSD < 5%	Milk Powder, Pork, Chicken Meat	[127]
	SPE	C_60_/rGO/NF	SWV (140 s)	0.25–34	0.21	PBS (7)	L, M, N, O	–	A, B, C, D, J	X, Y	^(3)^ 4.9% RSD, ^(4)^ 3.6% RSD, ^(5)^ 94–100%; 3.3% RSD	Synthetic Serum, Urine	[128]
Nitrofurantoin	GCE	rGO/β-CD	DPV	0.5–120	0.048	PBS (7)	L, M, N, O	–	A, B, C, D, F	X	^(1)^ 20 d. 93%, ^(2)^ 12.1 µA. µM^−1^.cm^−2^, ^(3,4)^ RSD < 4%, ^(5)^ 97.70-101.42%	Wastewater	[51]
Ofloxacin	GCE	AgNPs/MnO_2_/ErGO	DP-AdASV (500 s)	0.5−170	30	BR (4)	L, M, N, O	–	A, B, D, F. J	X, Y	^(1)^ 7 d. 4.7% RSD; 15 cyc., ^(2)^ 0.876 individual; 0.890 simultaneous, ^(3)^ 2.4% RSD, ^(4)^ 0.7% RSD, ^(5)^ 99.6–106%,	Tablet, River/Tap Water	[69]
	GCE	Zn_2_SnO_4_/rGO/NF	SWV (36 s)	0.0999–6.62	0.0828	H_2_SO_4_	L, M, N, O	–	A, B, C, D	X, Y	^(2)^ 8.21 A.mol^−1^.L, ^(3)^ 4.64% RSD, ^(4)^ 3.20% RSD, ^(5)^ 98.0–104.9%	Ophthalmic Solution	[129]
Penicillin-G	crGO	crGO/Aptamer A2	DPV, CV (30 s)	0.000001–1	0.00000124	PBS (6.5)	L, M, N, O	H	A, B, C, D, F	X, Z	^(1)^ 4 w., 6 cyc., ^(5)^ 1.13–85.42%	Milk, Meat, Eggs	[114]
Rifampicin	GCE	MoSe_2_/rGO/β-CD	DPV (360 s)	0.019–374.5	0.0028	PBS (7)	L, M, N	–	A, B, C, F	X, Z	^(2)^ 11.64 μA.μM^−1^.cm^−2^, ^(5)^ 99.26–98.9%, ^(6)^ RSD < 5%	Human Serum, Urine, Tilapia Fish, River Water	[130]
Streptomycin	PGE	RGO/AuNPs/Apt.	EIS, CV (12 min)	10^−10^–0.01	8 × 10^−13^	KCl (7)	L, M, N, O	–	A, B, C, D, F, J	X	^(1)^ 20 d. 4 °C 91%, ^(3)^ 6.1% RSD, ^(4)^ 5.1% RSD, ^(5)^ 89–103%,	Milk	[63]
Sulfamethoxazole	SPE	AA-RGO, CTAB	DPV (50 s)	0.5–50	0.04	PBS (6)	L, M, N, O	–	A, B, C, D, E, F, J	X	^(1)^ 14 d. 30 cyc. 92%, ^(5)^ 101.9–108.4%, ^(6)^ 10-fold	Lake Water, Tap Water, Synthetic Urine	[54]
Tetracycline	PGE	AuNPs/RGO/Aptamer	EIS (90 min)	1 × 10^−10^–1	3 × 10^−11^	– (7)	L, M, N, O	–	A, B, C, D, F, J	X	^(1)^ 21 d. 4 °C, ^(3)^ 5.6% RSD, ^(4)^ 4.2% RSD, ^(5)^ 92.8–102.1%	Milk	[132]
	PI	LIG/AuNPs/MIP (oPD+TC)	DPV (240 s)	0.01–0.30	0.00066	ABS (5.0)	L, M, N, O	–	A, B, C, D, E, F, J	–	^(1)^ 22 d. at 4 °C, ^(3,4)^ RSD 4.26–6.2%	Buffer, Milk, Meat Extract	[131]
Tetracyclines	CSPE	ERGO	AdTDPV (350 s)	20–80	12	PBS (6)	N, O	P, Q, S	J	X, Y	^(5)^ 97–104%	River Water, Skim Milk	[50]
Tobramycin	AuE	AuNWs/PDA-rGO	SWV (240 s)	0.000005–0.5	1.12 × 10^−7^	Tris-HCL (7.6)	L, M, N, O	–	A, B, C, D, F	X, Z	^(1)^ 9 d. at 4 °C, 90.1%, ^(3)^ 7.95% RSD, ^(5)^ 93.5–106.1%	Milk, Goat Milk	[133]
	GCE	rGO/PIM	SWV	0.0001–0.005	0.0001	HCL (5)	L, M, N, O	Q	A, B, C, D, F, J	X, Y	^(1)^ 10 d. transport, ^(5)^ 80.08%	Milk	[134]
^c^ Vancomycin ^d^ Ceftriaxone	GCE	Au-Ag-ANCCs/r-GO/poly(L-histidine)	SWV (205 s)	^c^ 0.000001–120 ^d^ 0.0001–290	^c^ 1.1 × 10^−7^ ^d^ 1.7 × 10^−8^	PBS (7)	L, M, N, O	–	A, B, C, D, F	X, Z	^(1)^ 8 w. at 4 °C ^c^ 94.45% and ^d^ 95.32, ^(3) c^ 4.23%; ^d^ 4.02% RSD, ^(4) c,d^ 1.72% RSD, ^(6)^ RSD < 5% for all interferents	Chicken, Meat, Fish, Milk	[58]

^a^—Amoxicillin; ^b^—Tetracycline; ^c^—Piroxicam; ^d^—Ofloxacin; ^y^—DPV; ^z^—CV. Analytical Characteristics—Advantages: (L) low LOD; (M) wide LDR; (N) selective electrode; (O) high reproducibility. Disadvantages: (P) high LOD; (Q) narrow LDR; (R) non-selective electrode; (S) low reproducibility of sensor; (H) sensor repeatability. Sensor Characteristics—Advantages: (A) low LOD; (B) wide LDR; (C) selective electrode; (D) high reproducibility; (E) easy modifier reparation; (F) long-term stability; (J) low cost. Disadvantages: (T) high LOD; (U) narrow LDR; (V) non-selective electrode; (W) low reproducibility of sensor; (X) complex modifier preparation; (Y) short-term stability; (Z) high cost. Analytical Performance Metrics: ^(1)^—Stability; ^(2)^—Sensitivity; ^(3)^—Reproducibility; ^(4)^—Repeatability; ^(5)^—Recoveries; ^(6)^—Selectivity/Specificity. d./w./m./cyc.—day(s)/week(s)/month(s)/cycle(s); RSD—Relative Standard Deviation.

**Table 4:** A meta-analysis on selected groups of electrochemical detection of antibiotics on graphene derivatives-based sensors and biosensors.

Group	Representative Sensors and Biosensors	Detection Matrix	Stability	Sensitivity	Reproducibility (RSD)	Recoveries (Avg.)	LDR Avg. (µM Range)	LOD Avg. (µM)	Ref.
LOD Lower End	GO/ZnO NRs, ERGO, AgNPs/MnO_2_/ErGO, MACGS, LEG	Water, Milk, Pharmaceutical and Meat Samples	7–90 days	0.2693 μA·μM^−1^–0.883 μA·μM^−1^·cm^−2^	Up to 2.4%	Up to 102.8%	6.3–91	12.5	[50,68,69,79,83]
LOD Middle-Performing	rGO@PDA@AuNPs, NiAl-LDH/rGO, Cu-Ag/GO, rGO/β-CD, GO/MWCNT	Milk, Wastewater, Milk, Tap Water, Eye Drops, Capsules, Serum	7–21 days	137.6 nA·µM^−1^·cm^−2^–12.1 µA µM^−1^ cm^−2^	Up to 4%	Up to 100.3%	5.244–365.62	0.155	[51,62,96,105,112]
LOD Upper End	GQD/CFX-BSA Abs, RGO/AuNPs/Apt., TA-Au-Ag-ANpM/r-GO/poly(glycine), Au-Ag-ANCCs/r-GO/poly(L-histidine), AuNPs/RGO/Apt.	Milk, Egg, Meat, Buffer, Fish	14–28 days	–	Up to 5.6	Up to 99.2%	2.06 × 10^−5^–140.402	9.75 × 10^−9^	[46,58,63,127,132]
Highly Sensitive	GR/Fe_3_O_4_NPs, FeMn_2_O_4_-rGO, Zn_2_SnO_4_/rGO/NF, N-prGO, rGO/MoS_2_	Antibiotic Plant Effluent, Urine, Serum, Tablet, Ophthalmic Solution	14–30 days	7.34 μA·μM^−1^·cm^−1^–9.645 × 10^4^ µA M^−1^	Up to 4.64%	Up to 101.5%	0.252–149.764	0.083	[57,89,115,121]
Highly Stable	MACGS, Ti_3_C_2_T_x_@Au-AS-GQD aerogel, Graphene, GO/MnFe_2_O_4_, Cl-RGO, rGO/Au/Co_2_CuS_4_	Buffer, Milk, Honey, Water, Calf plasma, Eye drops, Urine, Serum	30–90 days	0.1991 µA·µM–15.16 µA µM^−1^ cm^−2^	Up to 5.41%	Up to 102.05%	0.835–206.7	0.288	[45,79,84,118]
POC (ASSURED)	rGO, rGNR, AA-RGO/CTAB	Pharmaceuticals, Water, Synthetic Urine	Up to 14 days	–	Up to 4.47%	Up to 105.2%	3.125–31	0.585	[49,54,94]

**Table 5:** Real-sample validation performance of graphene-based sensors and biosensors for antibiotic detection.

Antibiotic	Matrix	Pretreatment Required	Recovery (%)	LOD in Real Sample	Validation Method	Ref.
Chloramphenicol	Milk	Defatting, centrifugation	96–102	0.0012 µM	Spike-recovery, HPLC cross-validation	[84]
Ciprofloxacin	River water	Filtration, pH adjustment	95–101	0.0018 µM	Standard addition, DPV	[86]
Tetracycline	Honey	Dilution (1:10) in PBS	98–104	0.08 µM	ELISA comparison	[95]
Sulfamethoxazole	Tap water	None	7.8–102	0.04 µM	LC-MS/MS confirmation	[94]
Metronidazole	Synthetic urine	Buffer dilution	94–100	0.21 µM	HPLC, SWV	[128]
Kanamycin	Milk	Protein precipitation	91–103	2.47 nM	Aptamer binding assay	[43]

**Table 6:** Cost–benefit comparison of traditional and electrochemical detection of antibiotics on graphene-based sensors and biosensors.

Method	Cost per Test	Time to Result	Infrastructure Required	Suitability for Low Recourse Settings
HPLC-MS/MS	$45–$120	4–8 h	Centralized lab, skilled operator	Low
ELISA	15-40	2–4 h	Basic lab equipment	Moderate
Graphene-based POC sensor	2-8	<10 min	Smartphone/portable reader	High

## Data Availability

The original contributions presented in this study are included in the article. Further inquiries can be directed to the corresponding author.

## Supplementary Materials

The following supporting information can be downloaded at: https://www.mdpi.com/article/10.3390/bios16050234/s1, Section S1: Materials and Methods; S1.1: Search Strategy and Information Sources; S1.2: Eligibility Criteria and Selection Process; S1.3: Study Risk of Bias Assessment; S1.4: Data Collection Process; S1.5: Quality Assessment Framework for the Examined Studies; S1.6: Meta-analysis; S1.6.1: Descriptive Meta-analysis; S1.6.2: Quantitative meta-analysis implementation details; Figure S1: A. Number of publications by year from database search results. B. PRISMA 2020 flow diagram for the included studies; Figure S2: Ontological classification of antibiotics illustrated through “paste SMILES” feature in ChemDraw (12.0.2); Figure S3: Comparative analytical performance of graphene-based (bio)sensors. Forest plots comparing the limit of detection (LOD) and absolute linear dynamic range (LDR) across all included studies, stratified by (A) antibiotic class, (B) (bio)sensor category; Table S1: Physicochemical properties of major antibiotic classes for electrochemical sensing on graphene-based platforms; Table S2: Data extraction protocol and coding scheme for systematic synthesis and meta-analysis.

## Abbreviations

The following abbreviations are used in this manuscript:

3D	Three Dimensional
4-NP	4-nitrophenol
β-CD	β-cyclodextrin
A2	35-base pen-aptamer
AA	Ascorbic Acid
ABS	Acetate Buffer Solution
Abs.	Antibodies
AFM	Atomic Force Microscopy
AMN	Azithromycin
AMOT	Amoxicillin trihydrate
AMP	Ampicillin trihydrate
AMR	Antimicrobial resistance
AMX	Amoxicillin
APS	Ammonium persulphate
Apt.	Aptamer
Apt-T1	Broad-spectrum aptamer
AUR	Aureomycin
BR	Britton–Robinson buffer
BSA	Bovine Serum Albumin
BTC	1,3,5-benzenetricarboxylicacid
C60	Fullerene
CA	Chronoamperometry
CA-CNTs	Copper alginate-carbon nanotube
CAP	Chloramphenicol
CB	Carbon Black
CEF	Ceftriaxone
CeW	Cerium (Ce)-doped tungsten oxide
CFM	Cefixime
CFX	Cephalexin
CHA	Catalytic hairpin assembly
CHIT	Chitosan
CIP	Ciprofloxacin
CLOX	Cloxacillin
CNNS	Graphitic carbon nitride nanosheet
CPE	Carbon Paste Electrode
crGO	Chemically Reduced Graphene Oxide
crl	Bayesian Credibility Intervals
CSPE	Carbon Screen Printed Electrode
CTAB	Cetyltrimethylammonium bromide
CuAAC	Copper-catalyzed azide-alkyne cycloaddition
CuPc	Copper Phthalocyanine Nanocomposites
CVD	Chemical Vapour Deposition
cyc.	Cycles (Stability)
d.	Number of days (Stability/Response Time)
DFT	Density Functional Theory
DOX	Doxycycline
DP-AdASV	Differential Pulse-Adsorptive Anodic Stripping Voltammetry
DPV	Differential Pulse Voltammetry
EA	Ethyl Acetate
EMIP	Electropolymerized molecular imprinted polymer
ErGO	Electro-reduced graphene oxide
Eu_2_O_3_	Europium oxide
Exo III	Exonuclease III
FAO	Food and Agriculture Organization
Fe/Zn-NaMMT	Fe/Zn-doped montmorillonite
FE-SEM	Field Emission Scanning Electron Microscopy
FEVO	Iron vanadium oxide nanoparticles (Fe0.11V2O5.16)
FF	Florfenicol
FLD	Furaltadone
fMWCNTs	Functionalized multiwalled carbon nanotubes
FTIR	Fourier-Transform Infrared Spectroscopy
FUZ	Furazolidone
GA-NH-YN	Alkyne-terminated graphene derivative
GARDP	Global Antibiotic Research & Development Partnership
GAT	Gatifloxacin
GCE	Glassy Carbon Electrode
GC-MS	Gas Chromatography-Mass Spectrometry
GdSnO	Gadolinium Stannate
GEN	Gentamicin
GM-P	GO and MXene (poly(dienyldimethylammonium chloride)) composite
GN	Graphene Composite
GNR	Graphene Nanoribbons
GNU	Gold Nanowires
GO	Graphene Oxide
GO-ZnO	Graphene Oxide-Zinc Oxide
GQD	Graphene Quantum Dots
GR	Graphene
h.	Number of hours (Response Time)
HKSJ	Hartung-Knapp-Sidik-Jonkman adjustment
HMIM PF_6_	1-hexyl-3 methylimidazolium hexafluorophosphate
HPLC	High-Performance Liquid Chromatography
HQ	Hydroquinone molecules
HRTEM	High-ResolutionTransmission Electron Microscopy
IL	Ionic Liquid
i-t AMP	Amperometry
KAN	Kanamycin
KAS	Kasugamycin
LCS	Lignocellulosic Carbon Sheets
LDH	Layered Double Hydroxide
LEG	Laser Enabled Graphene
LEV	Levofloxacin
LOQ	Limit of quantification
LSV	Linear Scan Voltammetry
LZD	Linezolid
m.	Number of months (Stability)
MACGS	Ca^2+^-MgAl_2_O_3_-G-SiO_2_
MAP	Mesaminophenol
MIO@NG	Magnetic iron oxide embed nitrogen-doped graphene
MIP	Molecularly Imprinted Polymer
MNZ/MTZ	Metronidazole
MOF	Metal Organic Framework
MoSe2	Molybdenum diselenide
MPM	Meropenem
MSPE	Magnetic screen-printed electrode
MWCNT	Multiwalled Carbon Nanotubes
NCs	Nanocomposites
NEO	Neomycin
NF	Nafion
NFT	Nitrofurantoin
NH_2_–UIO	2-amino-terephthalate-functionalized zirconium (IV)-based MOF
NPs	Nanoparticles
NRs	Nanorods
NSs	Nanosheets
OFX	Ofloxacin
OMC	Ordered mesoporous carbon
oPD	o-phenylenediamine
ORN	Ornidazole
OXCL	Oxacillin sodium salt
OXY	Oxytetracycline
P-11-1	Truncated 47 nucleotides aptamer
PBS	Phosphate Buffer Solution
PDDA-Gr	Poly (diallyldimethylammonium chloride)-functionalized graphene (PDDA-Gr)
PEI	Polyethyleneimine
Pen-G	Penicillin-G
PET	Polyethylene terephthalate
PI	Polyimide (material type)
PI	Prediction Intervals (quantitative meta-analysis)
PIM	Polymer Inclusion Membrane
PLA	Polylactic Acid-based filament
POC	Point-of-care
poly(SER)	Polyserine film
PRX	Piroxicam
PSB	Graphene-like biochar from waste peanut shells
PSS	Polystyrene Sulfonate
PtE	Platinum Electrode
QDs	Quantum Dots
RE	Random Effects
rGNR	Reduced graphene nanoribbons
rGO/RGO	Reduced Graphene Oxide
RIF	Rifampicin
RRDE	Rotating ring disk electrode
RSD	Relative Standard Deviation
SDG(s)	Sustainable Developmental Goal(s)
SDZ	Sulfadiazine
SELEX	Systematic evolution of ligands by exponential enrichment
SEM	Scanning Electron Microscopy
SMILES	Simplified Molecular Input Line Entry System
SmMoSe2	Samarium-Substituted Molybdenum Diselenide
SMX	Sulfamethoxazole
SMZ	Sulfamethazine
SP	Screen Printed
SPE	Screen Printed Electrode
STZ	Sulfathiazole
SWV	Square Wave Voltammetry
TC	Tetracycline
TC-MIP	Tetracycline Molecularly Imprinted Polymer
TDM	Therapeutic Drug Monitoring
TE	Tris-EDTA
TEM	Transmission Electron Microscopy
TMP	Trimethoprim
TOB	Tobramycin
UHC	Universal Health Coverage
UN	United Nations
UNEP	United Nations Environment Program
VAN	Vancomycin
w.	Number of weeks (Stability)
WOAH	World Organization for Animal Health
XPS	X-ray Photoelectron Spectroscopy
