# Supplementary material for: Recent Advances in Electrochemical Detection of Antibiotics on Graphene-Based Sensors and Biosensors, Impact and Sustainable Development Challenges: A Systematic Review and Meta-Analysis
Source: Biosensors (Basel). 2026 Apr 23;16(5):234. doi: 10.3390/bios16050234 (PMC13204706; doi:10.3390/bios16050234)
Supplement: Supplementary file 1 [file biosensors-16-00234-s001.zip › biosensors-4221339-supplementary.pdf]

Supplementary Materials

Recent Advances in Electrochemical Detection of Antibiotics on Graphene-Based Sensors and Biosensors, Impact and Sustainable Development Challenges: A Systematic Review and Meta-Analyses

Muhammad Saqib [1,2,*], Mrinal Vashisth [3], Elena I. Korotkova [1], Amrit L. Hui [4], Stephen O. Aremu [5], Souvik Das [6], Aniruddha Deb [7], Nirmal K. Hazra [8], Rachita Saha [9], Subrata Saha [9] and Pradip Kumar Kar [9]

[1] Chemical Engineering Division, School of Earth Sciences and Engineering, National Research Tomsk Polytechnic University, 30 Lenin Avenue, 634050 Tomsk, Russia
[2] UNESCO Laboratory of Environmental Electrochemistry, Department of Analytical Chemistry, Faculty of Science, Charles University, Hlavova 8/2030, CZ 128 43 Prague 2, Czech Republic
[3] Higher Engineering School of Agrobiotech, National Research Tomsk State University, 36 Lenin Avenue, 634050 Tomsk, Russia
[4] National Research Tomsk State University, 36 Lenin Avenue, 634050 Tomsk, Russia
[5] Faculty of General Medicine, Siberian State Medical University, 634050 Tomsk, Russia
[6] Department of Industrial Design, National Institute of Technology Rourkela, Sundargarh 769008, India
[7] Department of Chemical Engineering, Birla Institute of Technology Mesra, Ranchi 835215, India
[8] Department of Chemistry, Coastal Environmental Studies Research Centre, Egra Sarada Shashi Bhusan College, Vidyasagar University, Egra 721429, India
[9] Parasitology Laboratory, Department of Zoology, Cooch Behar Panchanan Barma University, Vivekananda Street, Cooch Behar 736101, India
* Correspondence: saqibm@natur.cuni.cz

S1. Materials and Methods

S1.1. Search Strategy and Information Sources

The systematic literature search encompassed two bibliometric databases, PubMed and Scopus between January 2016 until December, 2025 (10 years). To ensure coverage, a dual search strategy was perfomed in PubMed: (i) a controlled vocabulary using Medical Subjects Headings (MeSH) combining terms for: *("graphene"[Title/Abstract] OR "graphene oxide"[Title/Abstract]) AND ("Electrochemical Techniques"[Mesh]) AND ("Biosensing Techniques"[Mesh]) AND ("Anti-Bacterial Agents"[Mesh]).* After applying filters for publication type (*Review, Scoping Review, Systematic Review*) and restricting results to 10 years); and (ii) a non-MeSH query keyword approach utilizing strings for *("sensor*" OR "biosensor*") AND ("graphene" OR "graphene oxide" OR "reduced graphene oxide") AND (("electrochemical") AND ("detection" OR "determination" OR "sens*" OR "biosens*") AND ("antibiotic*").*

Scopus database was queried using keyword combinations used as follows: *("electrochemical biosensor" OR "electrochemical sensor") AND ("graphene" OR "graphene oxide" OR "reduced graphene oxide" OR "graphene derivatives") AND ("Antibiotics" OR "antibiotic agents").* Each database was initially queried without applying filters to maximize bibliometric coverage. The review protocol was prospectively registered in the Open Science Framework (OSF) registry to ensure methodological transparency and facilitate replication.

S1.2. Eligibility Criteria and Selection Process

The inclusion criteria were defined to ensure a focused selection of studies investigating electrochemical graphene-based sensors and biosensors for antibiotic identification. Eligible publications were constrained to original research articles and conference proceedings published within the last decade that reported empirical electrochemical detection data. The target object was included only if it was identified as an antibiotic (antimicrobial agent) with either Chemical Entities of Biological Interest (ChEBI) or CARD's Antibiotic Resistance Ontology (ARO) datasets. The latest versions of these datasets were downloaded from NCBO BioPortal. Publications were excluded if they were narrative/systematic reviews, off-topic, duplicated, lacked necessary data-fields described in subsequent sections, and focused on non-antibiotic compounds (failed to meet established ontology criteria). Additionally, pre-prints were also excluded from critical appraisal.

The systematic review adhered to PRISMA 2020 guidelines for reporting standards. Retrieved records were systematically imported and duplicates were resolved through DOIs using drop_duplicates() and Zotero PDF import steps (feature to identify duplicates). Titles and abstracts were screened proactively for eligibility criteria, resulting in the exclusion of additional 60 records. The remaining articles underwent full-text evaluation, leading to the removal of 13 additional studies. After critical appraisal step, 94 records were selected for inclusion. The complete selection process, including yearly publication trends, is detailed in the PRISMA flow diagram (Figure S1).

S1.3. Study Risk of Bias Assessment

Given the analytical and materials-focused nature of the included literature, risk of bias was assessed proactively using established electrochemical sensor validation and ontological compliance frameworks rather than clinical diagnostic tools. Each study was evaluated for methodological rigor, focusing on sensor reproducibility, electrochemical parameter reporting, calibration transparency, and accurate antibiotic identification via ChEBI and ARO ontologies (Figure S2). Studies were systematically appraised and categorized based on adherence to analytical reporting standards and chemical annotation completeness. This approach ensured that only methodologically sound and chemically well-annotated studies advanced to data synthesis. Furthermore, the details regarding Physicochemical properties of major antibiotic classes from the examined studies are provided in Table S1.

S1.4. Data Collection Process

A systematic data extraction protocol was applied to all 94 eligible publications. These variables, along with corresponding processing steps are described in Table S2. Studies were categorized based on graphene derivative type, electrochemical detection modality (amperometric, impedimetric, voltammetric), target antibiotic class, biosensing architecture and nanomaterials used in the study. Key data points extracted included limit of detection (LOD), linear dynamic range (LDR), sensing matrix, sample type, and analytical performance metrics which also functioned as a custom quality control framework. Additionally, antibiotic classifications and standardized three/four-letter abbreviations were mapped from the ARO and ChEBI databases to ensure uniform nomenclature across the review. In absence of a standard two/three/four letter abbreviation, a uniform defined

abbreviation was employed throughout the analysis. All extracted data were compiled into structured tables for comparative evaluation, with comprehensive details on antibiotic group information for ease of grouping. Additionally, the sensing materials were also grouped (described in later sections). Each data-field variable type, specifics, collection purpose and processing criteria are described in Table S2. The absence of any data-field was registered with a '–' symbol in the coding scheme.

S1.5. Quality Assessment Framework for the Examined Studies

Each study was evaluated using adapted criteria: (1) experimental design rigor (replicate measurements, control experiments); (2) potential bias sources (sample selection, measurement bias); (3) reproducibility indicators (RSD values, inter-day/intra-day variability); (4) LOD/LDR validation (signal-to-noise ratio, calibration curve linearity R^2>0.99); (5) real-sample validation (recovery studies, matrix effects). Studies were categorized as high, moderate, or low quality based on cumulative scores across five reporting dimensions: sensitivity, stability, reproducibility, repeatability and recoveries in the quantitative meta-analysis.

S1.6. Meta-analysis

As this review emphasizes qualitative synthesis and analytical performance trends rather than clinical meta-analysis, descriptive statistics and comparative subgroup analyses were primarily utilized. However, additional quantitative meta-analyses were performed for three reporting metrices: LOD, absolute LDR width, and response time. Yearly publication trends and bibliographic summaries were graphically summarized. Where quantitative aggregation was feasible, ranges and central tendency measures for analytical figures of merit (e.g., LOD, sensitivity) were calculated from 109 rows (n=94 studies) however additional statistical group-wise comparisons with significance estimates were not performed. Additionally, Comparative LOD and LDR metrices were plotted for all studies, individual antibiotics, antibiotic groups and nanomaterial (sensor type) groupings. Additional, group-wise summaries, sensor and biosensor representative examples were summarised. Moreover, point-of-care readiness was assessed for studies scoring high on ASSURED criteria. Finally, quantitative meta-analysis were performed using random effects model for the qualifying studies. Heterogeneity in sensor performance was addressed through stratification by graphene material class, nanomaterial type, electrochemical technique, and antibiotic target family and confidence, prediction and Bayesian credibility intervals were computed wherever applicable.

S1.6.1. Descriptive Meta-analysis

The examined graphene derivatives-based studies were compared for their performance metrics LOD and LDR in a single forest plot. We performed the analysis in the Python programming language (3.9.20) with data preprocessing and cleaning steps using NumPy (1.26.4) and Pandas (2.2.3) libraries. The figures were plotted using plotly (6.1.1). For this purpose, the raw table were organized by flattening each entry into multiple fields including: type of material, electrode substrate, sensing martials, antibiotic, two/three/four-letter abbreviation of the antibiotic, matrix of detection, advantage and disadvantages of analytical and sensor characteristics (four columns), electrochemical method, accumulation time, and additional columns that registered reporting metrics: sensitivity, stability, reproducibility, repeatability, recoveries, portability, role of electrode

regeneration, validation of matrix effect, multiplexing, cost of scalability, and challenges for calibration (uniform or non-uniform). The post-processing steps were performed to ensure the readability of the metanalysis figures. All plots were exported as PNG with minimal margins and axis labels at scale 2 with A4 paper dimensions using Plotly's kaleido engine. The global and facet means were computed during plot generation but adjusted in post-processing steps in GIMP 3.0 to improve readability such that they were displayed without overlaps. For the smaller doughnuts, the labels were converted to larger font without altering the figure dimensions to improve the readability.

For all reviewed sensors and biosensors presented in main manuscript Tables 1–3 we converted the presence or absence of the following analytical performance metrics: (i.) reported stability, (ii.) declaration of sensitivity measure, (iii.) indication of RSD values for reproducibility and (iv.) repeatability, and (v.) reported measure for recoveries. Additionally, binary coding was performed for indications of the following capabilities: (vi.) requirement for sample preparation, (vii.) real-time analysis; from the dataset columns; real-time capability was assigned if the overall detection time was under 60s., (viii.) portability. Finally, common challenges were coded such as: (ix.) matrix effects, (x.) multiplexing capability for multiple target analytes, (xi.) issues with calibration, (xii.) issue with scalability due to cost of modifier used for (bio)sensor preparation. Together, items (i)–(v) indicate the objective quality of findings, (vi)–(viii) along with additional codes to represent the POC-readiness of the fabricated bio(sensor) was evaluated objectively according to the ASSURED criteria from collected data-fields described in Table S2 and (ix)–(xii). indicate common scalability and performance issues.

S1.6.2. Quantitative meta-analysis implementation details

The pandas and numpy libraries were used for data handling, while matplotlib 3.9.4 (pyplot, patches, gridspec and lines) were used for plotting, with seaborn (0.13.2) used for uniform styling. The forest plots were exported at 600 dpi resolution and additional post-processing was perfomed in GIMP 3.0 software. The LOD (μM) and LDR (absolute width in μM) and response time (s) metrices were evaluated for all examined studies. A custom framework was employed consisting of five reporting dimensions for quality control (reports of stability, sensitivity, reproducibility, repeatability and recoveries). At first LDR absolute width was computed by substracting the upper and lower end of the LDR. A filter was applied for quality score ≥ 3 and atleast 3 studies within each group. The nanomaterial groupings included: Additives/surfactants/binders (CTAB, IL, NF, SDS, etc.), Bimetallic/multimetallic nanostructures (Au-Ag, PtPd, etc.), Bimetallic/multimetallic nanostructures (Au-Ag, PtPd, etc.), Biological recognition elements (aptamers, antibodies, DNA, SELEX), Carbon-based nanomaterials (pristine graphene, CNTs, GQDs, biochar, rGO, etc.), Cyclodextrins/host-guest molecules (β-CD, etc.), Layered Double Hydroxides (NiAl-LDH, etc.) Metal oxides (NiO, ZnO, ferrites, vanadates, etc.), Metal sulfides/chalcogenides (CdS, Ag_2S, Ag_2Se, CuS, etc.), Metal-Organic Frameworks (Cu-BTC, NiCo MOF, UIO-66, etc.), Noble metals/metallic nanoparticles (Au, Pt, Pd, Ag, etc.), Polymers/Molecularly Imprinted Polymers (PANI, PIM, MIP, PLA, etc.), Transition Metal Dichalcogenides (MoS_2, $MoSe_2$, etc.).

The LOD and response time metrics were log transformed to stabilize

the variance and prevent negative CI bounds. Within-group sample variance was computed under an equal-variance assumption due to missing study-level standard errors. Equal inverse-variance weigths were assigned, and pooled emans were computed along with SE estimates. A 95% CI was then computed according to t-distribution (DF=k-1) with HKSJ adjustment for small-sample accuract. The PIs display expected effect ranges in future studies. The PIs were calculated as follows:

$$PI = \mu \pm t(k-2) \times \sqrt{SE^2 + \tau^2}$$

Additionally, for groups with n ≤ 4 studies, a Bayesian random-effects model with weakly informative priors were used for estimate stabelisation. The convergence was assessed via $\hat{R}$ statistic. While reporting the log-transformed values were back-transformed to original units with pooled means reported as geometric means. The Cochrane recommendations were used for classification of I^2 (<25%: Low, 25–50%: Moderate, 50–75%: Substantial, ≥75%: Considerable). Based on this criteria, random-effects model with HKSJ adjustment was selected as the primary analysis method. The fixed-effect model was implemented only for sensitivity analysis. Due to high heterogeneity, a DerSimonian-Laird estimator for τ^2 with HKSJ adjustment was implemented. The first log-transformation for all except for LDR absolute width variable. Subsequently, Cochran's Q statistic, τ^2 and RE weights were computed. The RE pooled means and SE with 95% CI were computed as well. The Bayesian credible intervals were computed for all qualifying groupings via Gibbs sampling (5000 iteration, 20% burn-in). Weakly informative priors were used for μ approximating Normal (0, 10^2) and σ and τ approximating HalfNormal(2) to allow plausible variability. Finally, GRADE certainty was assessed by downgrading a maximum score of 4 (++++) for high uncertainty, small sample sizes, wide CIs and undetermined publication bias to 1 (+).

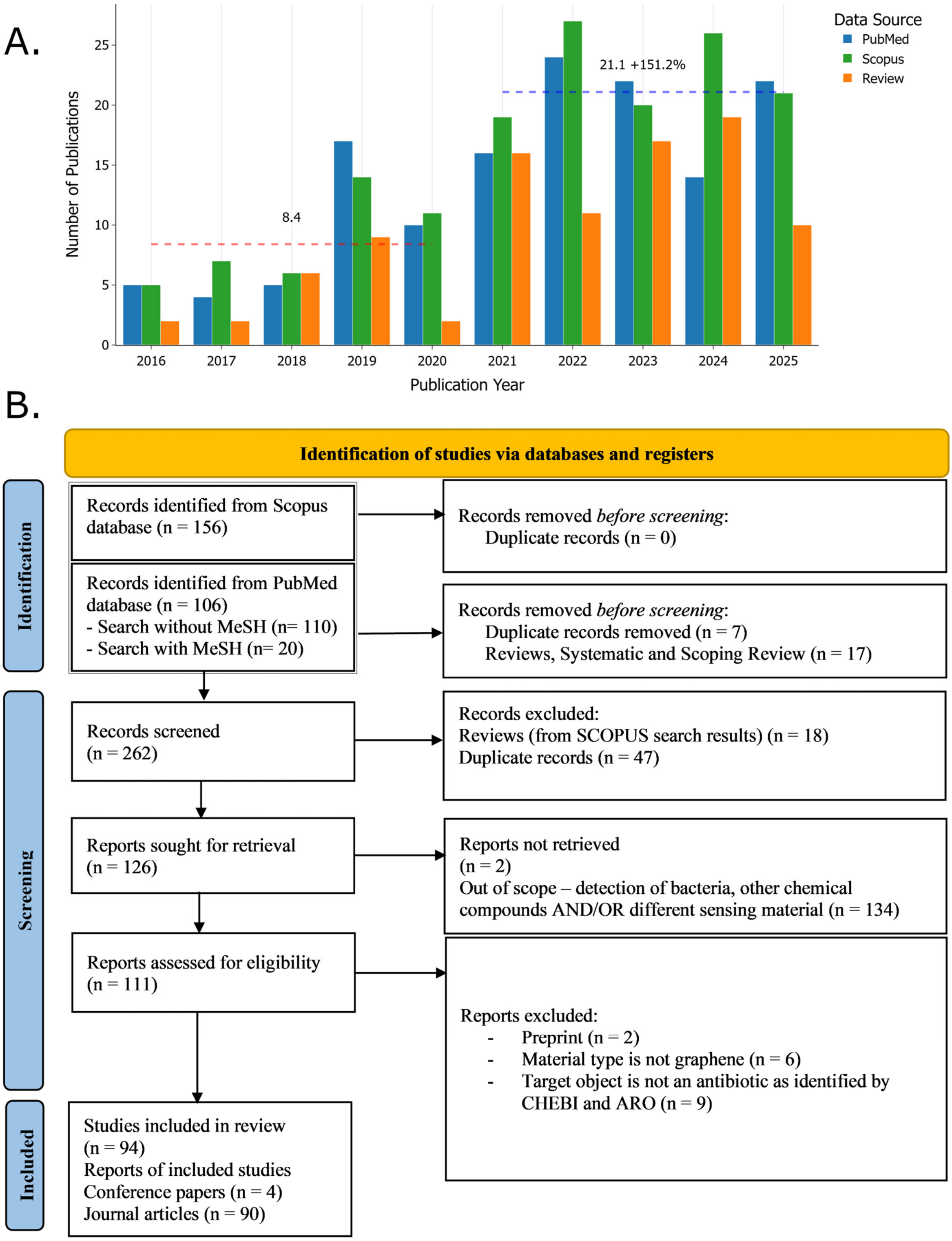

ARO – Antibiotic Resistance Ontology, CHEBI – Chemical Entities of Biological Interest Ontologies, NCBO – National Centre for Biomedical Ontology, MeSH – Medical Subject Headings

Figure S1. A. Number of publications counts by year from database search results. B. PRISMA 2020 flow diagram for the included studies

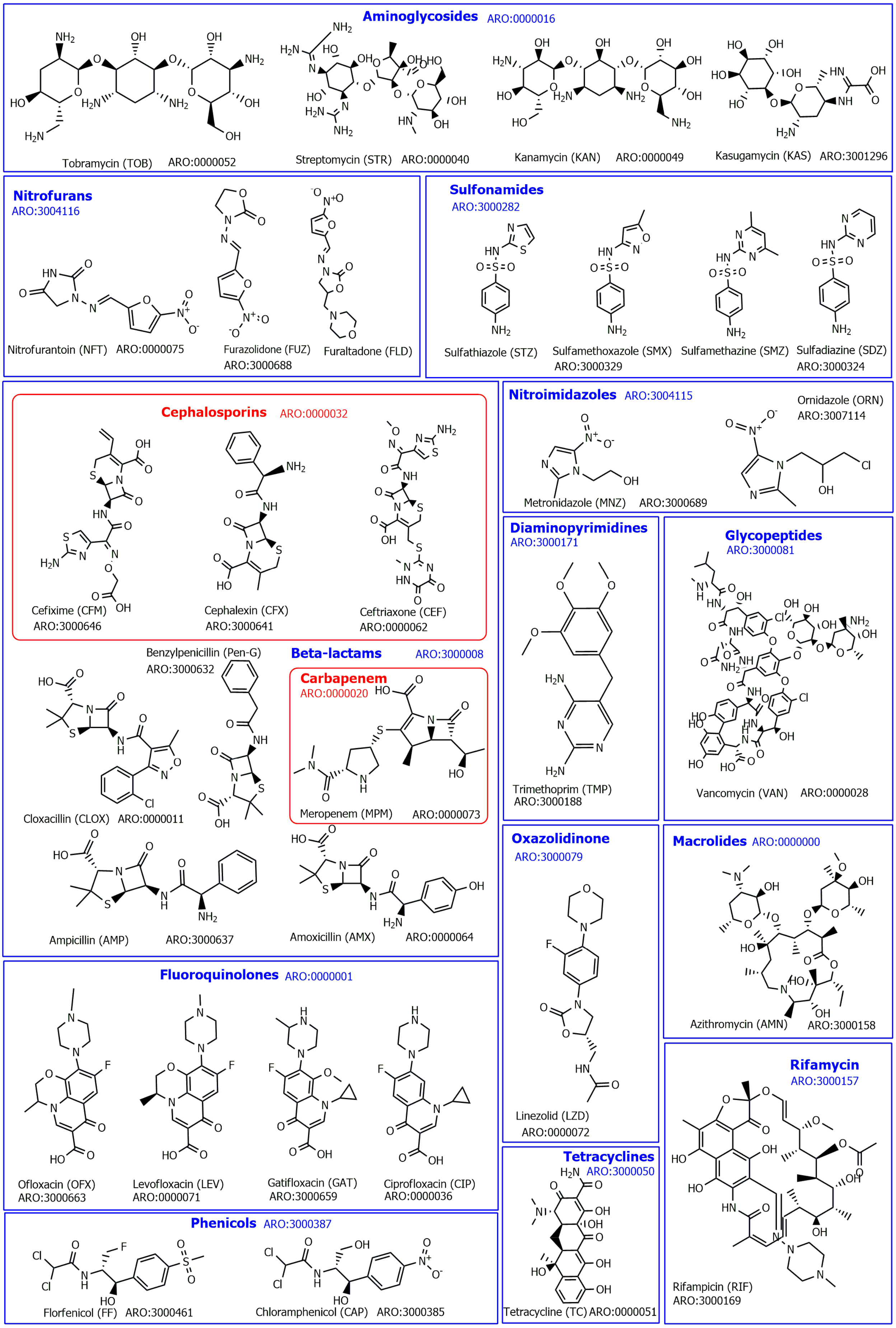

Figure S2. Ontological classification of antibiotics illustrated through 'paste SMILES' feature in ChemDraw (12.0.2)

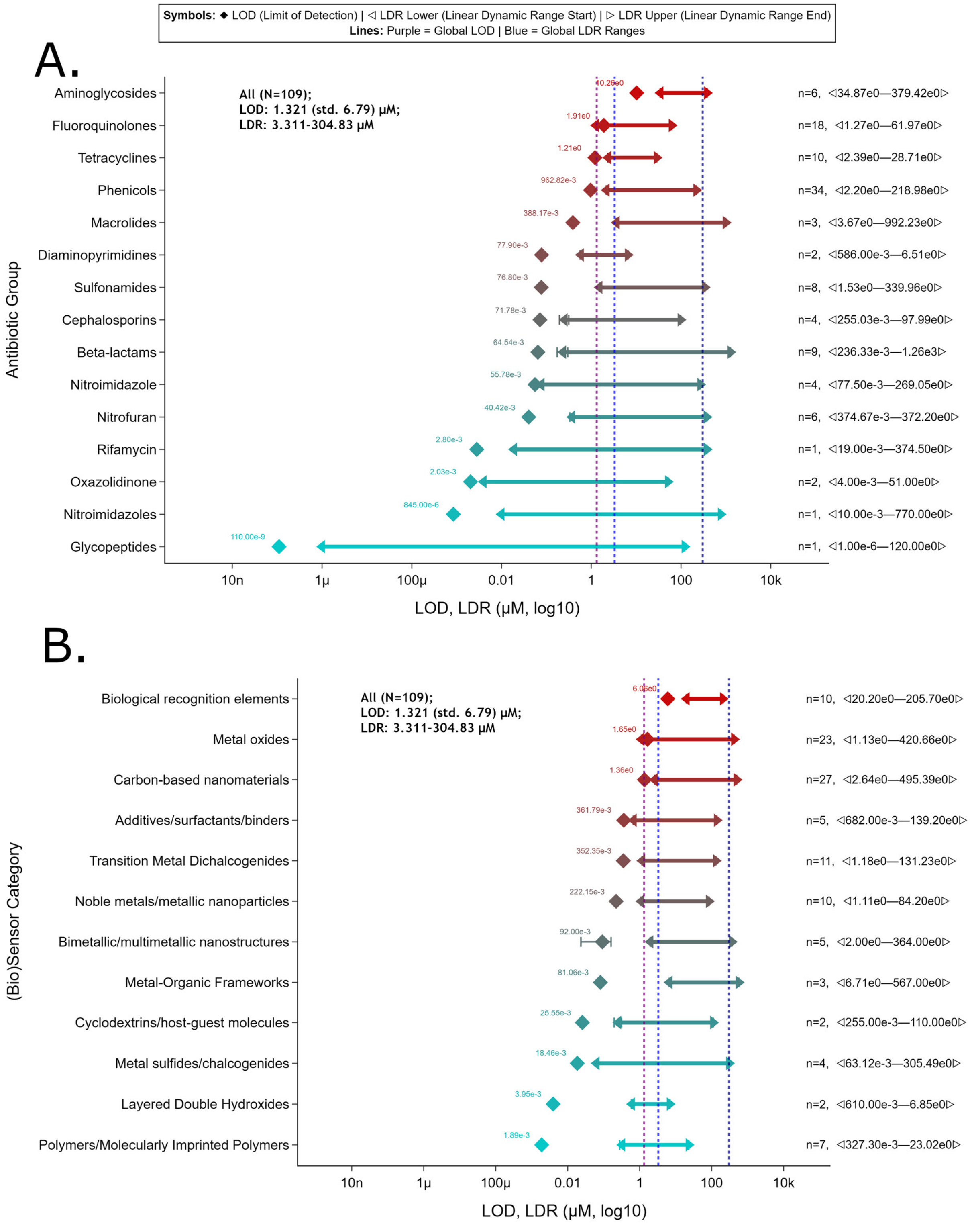

Figure S3. Comparative analytical performance of graphene-based (bio)sensors. Forest plots comparing the limit of detection (LOD) and absolute linear dynamic range (LDR) across all included studies, stratified by: (A) antibiotic class. (B) (bio)sensor category/architecture

Table S1. Physicochemical properties of major antibiotic classes for electrochemical sensing on graphene-based platforms

Antibiotic Class	Representative Compounds (Abbrev.)	Approx. MW (g/mol)	Key pKa Values*	Log P / Hydrophobicity	Aqueous Solubility	Redox-Active Functional Groups	Primary Electrochemical Detection Mechanism on Graphene	Preferred Graphene Derivative & Rationale
**β-Lactams (Penicillins, Cephalosporins)**	Amoxicillin (AMX), Ampicillin (AMP), Penicillin-G (Pen-G), Cephalexin (CFX), Cefixime (CFM), Ceftriaxone (CEF)	350–550	2.5–3.0 (COOH); 7.0–7.5 (NH_2)	−1.5 to 0.5 (hydrophilic)	High (polar, ionizable)	Phenolic –OH (AMX); β-lactam carbonyl; aromatic rings	Enzyme-mediated oxidation (laccase/AuNP); indirect detection via biorecognition (aptamer/antibody); steric hindrance "signal-off"	GO: abundant –COOH/–OH for H-bonding with β-lactam carbonyls; biocompatible surface for antibody/aptamer immobilization
**Fluoroquinolones**	Ciprofloxacin (CIP), Levofloxacin (LEV), Gatifloxacin (GAT), Ofloxacin (OFX)	330–400	~6.0 (carboxyl); ~8.8 (piperazinyl N)	0.3–1.5 (amphiphilic/zwitterionic)	Moderate (pH-dependent)	Piperazinyl secondary amine (oxidizable); conjugated quinolone core (π-system); carboxylate	Irreversible $2e^-/2H^+$ oxidation of piperazinyl ring; π–π stacking preconcentration on sp^2 carbon	rGO/GR: restored sp^2 network enables strong π–π stacking with planar quinolone core; optimal at pH 3–5 for protonation-enhanced adsorption
**Tetracyclines**	Tetracycline (TC), Doxycycline (DOX), Oxytetracycline (OXY)	440–480	3.3 (tricarbonyl); 7.7 (dimethylamino); 9.7 (phenolic)	−1.0 to −0.3 (hydrophilic)	Moderate–High (pH-dependent)	Phenolic –OH; tricarbonyl system; dimethylamino group	Direct oxidation of phenolic/dimethylamino moieties; cation-π interactions with Cu^{2+}-doped surfaces; MIP/aptamer binding	GO/rGO hybrid: GO for H-bonding/electrostatic capture of zwitterionic forms; rGO for conductivity; pH 6–8 optimal for cation-π
**Aminoglycosides**	Kanamycin (KAN), Streptomycin (STR), Gentamicin (GEN), Tobramycin (TOB)	450–580	7.0–9.5 (multiple amino groups)	−3.0 to −1.0 (highly hydrophilic, polycationic)	Very High (water-soluble salts)	Primary/secondary amines (oxidizable); glycosidic hydroxyls	Indirect detection via aptamer conformational change ("signal-off"); electrostatic preconcentration on anionic surfaces	GO: high density of anionic $–COO^-$ at neutral pH enables strong electrostatic attraction to polycationic aminoglycosides; rGO less effective due to charge repulsion
**Sulfonamides**	Sulfamethoxazole (SMX), Sulfamethazine (SMZ), Sulfadiazine	250–280	~2.0 (sulfonamide NH); ~5–7 (aniline NH_2)	0.5–1.2 (moderately	Low–Moderate (pH-	Aromatic primary amine ($–NH_2$, oxidizable); sulfonamide (–	Irreversible $2e^-/2H^+$ oxidation of aromatic amine; electrostatic	rGO + CTAB/surfactant: cationic modifier attracts anionic sulfonamides at neutral pH; rGO provides

	(SDZ), Sulfathiazole (STZ)			hydrophobic)	dependent)	SO_2NH–); heterocyclic N	preconcentration via cationic surfactants	conductive π-platform for oxidation
**Nitrofurans / Nitroimidazoles**	Chloramphenicol (CAP), Florfenicol (FF), Metronidazole (MNZ), Nitrofurantoin (NFT), Furazolidone (FUR), Furaltadone (FLD)	250–350	CAP: ~5.5 (amide); MNZ: ~2.6 (imidazole N)	CAP: 1.1 (moderate); MNZ: −0.1 (hydrophilic)	CAP: Low; MNZ/NFT: Moderate	Nitro group (–NO_2, reducible); amide (CAP); imidazole ring (MNZ)	Irreversible $4e^-/4H^+$ cathodic reduction of –NO_2 to –NHOH; catalytic enhancement by metal/metal-oxide NPs	rGO + catalytic NPs (Fe_3O_4, MoS_2, Eu_2O_3): rGO enhances electron transfer kinetics; NPs lower overpotential for nitro reduction; GO useful for CAP H-bonding via –OH/–COOH

Table S2. Data extraction protocol and coding scheme for systematic synthesis and meta-analysis

Variable	Variable Type	Specifics	Purpose	Processing
**Type**	String	Graphene, Graphene Oxide, Reduced Graphene Oxide	Grouping, Meta-analyses	Raw
**Electrode substrate**	String	Various: Reported electrode substrate(s)	Reporting	Raw
**Sensing materials**	String	Various: Reported sensing material(s)	Reporting	Raw
**Antibiotic**	String	Various: Antibiotic name (ARO, ChEBI); unique entries separated within studies	Plotting	Normalised
**Ab. Abbreviation**	String	Three letter abbreviations	Plotting	Normalised
**Matrix**	String	Various	Reporting	Raw
**Parent Class Label**	String	Antibiotic group (ARO, ChEBI)	Grouping, Meta-analyses	Raw
**Category**	String	Sensor category (custom)	Grouping, Meta-analyses	Normalised
**Category Member Examples**	String	Sensor category assignment logic for coder's reference	Reporting	Normalised
**LDR Lower**	Numerical	Lower end of the LDR	Analyses	Normalised to μM
**LDR Upper**	Numerical	Upper end of the LDR	Analyses	Normalised to μM
**LOD**	Numerical	Reported LOD	Analyses	Normalised to μM
**Elec. Method**	String	Reported electrochemical method	Analyses	Normalised
**Acc. (s)**	Numerical	Reported response time	Analyses	Normalised to seconds
**Medium**	String	Various: Reported medium of detection	Reporting	Raw
**pH**	Numerical	Reported pH of detection	Reporting	Raw
**AC Adv.**	Object	L, M, N, O	Analyses, ASSURED	Coded
**AC Disadv.**	Object	P, Q, R, S, H	Analyses, ASSURED	Coded
**EC Adv.**	Object	A, B, C, D, E, F, J	Analyses, ASSURED	Coded
**EC Disadv.**	Object	T, U, V, W, X, Y, Z	Analyses, ASSURED	Coded
**Stability**	String	Reporting/ Quality Control	Quality Control, Meta-analyses	Coded
**Sensitivity**	String	Reporting	Quality Control, Meta-analyses	Coded
**Reproducibility**	String	Reporting	Quality Control, Meta-analyses	Coded
**Repeatability**	String	Reporting	Quality Control, Meta-analyses	Coded
**Recoveries**	String	Reporting	Quality Control, Meta-analyses	Coded

**Portability**	String	Binary: Portable/ Not Portable	Analyses, ASSURED	Coded
**Multiplexity**	String	Binary: Multi/ Single	Analyses, ASSURED	Coded
**Matrix Effects**	String	Binary: Validated/ Unvalidated	Analyses, ASSURED	Coded
**RT Analysis**	String	Binary: Real Time/ Not-Real Time	Analyses, ASSURED	Coded
**Sample preparation**	String	Binary: Required/ Not Required	Analyses, ASSURED	Coded
**Calibration**	String	Binary: Linear/ Non-linear	Analyses, ASSURED	Coded
**Scalability**	String	Binary: Scalable/ Not Scalable	Analyses, ASSURED	Coded
**Reference**	String	Reporting: Author, Year	Reporting	Raw
